# Composition differences between organic and conventional meat: a systematic literature review and meta-analysis

**DOI:** 10.1017/S0007114515005073

**Published:** 2016-03-28

**Authors:** Dominika Średnicka-Tober, Marcin Barański, Chris Seal, Roy Sanderson, Charles Benbrook, Håvard Steinshamn, Joanna Gromadzka-Ostrowska, Ewa Rembiałkowska, Krystyna Skwarło-Sońta, Mick Eyre, Giulio Cozzi, Mette Krogh Larsen, Teresa Jordon, Urs Niggli, Tomasz Sakowski, Philip C. Calder, Graham C. Burdge, Smaragda Sotiraki, Alexandros Stefanakis, Halil Yolcu, Sokratis Stergiadis, Eleni Chatzidimitriou, Gillian Butler, Gavin Stewart, Carlo Leifert

**Affiliations:** 1 Nafferton Ecological Farming Group (NEFG), School of Agriculture, Food and Rural Development, Newcastle University, Nafferton Farm, Stocksfield, Northumberland NE43 7XD, UK; 2 School of Agriculture, Food and Rural Development, Human Nutrition Research Centre, Newcastle University, Agriculture Building, Kings Road, Newcastle upon Tyne NE1 7RU, UK; 3 School of Biology, Newcastle University, Ridley Building, Newcastle upon Tyne NE1 7RU, UK; 4 Benbrook Consulting Services, 90063 Troy Road, Enterprise, OR 97828, USA; 5 Food and Agriculture Division – Grassland and Forage, Norwegian Institute of Bioeconomy Research (NIBIO), Gunnars veg 6, N-6630 Tingvoll, Norway; 6 Department of Dietetics, Faculty of Human Nutrition and Consumer Sciences, Warsaw University of Life Sciences, Nowoursynowska 159c, 02-776 Warsaw, Poland; 7 Department of Functional and Organic Food and Commodities, Faculty of Human Nutrition and Consumer Sciences, Warsaw University of Life Sciences, Nowoursynowska 159c, 02-776 Warsaw, Poland; 8 Department of Animal Physiology, Faculty of Biology, University of Warsaw, Miecznikowa 1, 02-096 Warsaw, Poland; 9 Department of Animal Medicine, Production and Health, University of Padua, Viale dell’ Università 19, 35020 Legnaro, Italy; 10 Department of Food Science – Food Chemistry & Technology, Aarhus University, Blichers Allé 20, Building F20/8845, 8830 Tjele, Denmark; 11 Research Institute for Organic Agriculture (FiBL), Ackerstrasse 113, CH-5070 Frick, Switzerland; 12 Institute of Genetics and Animal Breeding, Polish Academy of Science, Jastrzębiec, Postępu 36, 05-552 Magdalenka, Poland; 13 Human Development and Health Academic Unit, Faculty of Medicine, University of Southampton, Southampton SO16 6YD, UK; 14 National Agricultural Research Foundation (NAGREF), Veterinary Research Institute of Thessaloniki, 57001 Thermi, Thessaloniki, Greece; 15 Kelkit Aydin Vocational Training School, Gumushane University, 29600 Kelkit, Gumushane, Turkey; 16 Food Production and Quality Division, School of Agriculture, Policy and Development, Centre for Dairy Research, University of Reading, PO Box 237, Earley Gate, Reading RG6 6AR, UK

**Keywords:** Organic foods, Animal products, Meat, Iron, Meat fat composition, *n*-3 PUFA, *n*-6 PUFA

## Abstract

Demand for organic meat is partially driven by consumer perceptions that organic foods are more nutritious than non-organic foods. However, there have been no systematic reviews comparing specifically the nutrient content of organic and conventionally produced meat. In this study, we report results of a meta-analysis based on sixty-seven published studies comparing the composition of organic and non-organic meat products. For many nutritionally relevant compounds (e.g. minerals, antioxidants and most individual fatty acids (FA)), the evidence base was too weak for meaningful meta-analyses. However, significant differences in FA profiles were detected when data from all livestock species were pooled. Concentrations of SFA and MUFA were similar or slightly lower, respectively, in organic compared with conventional meat. Larger differences were detected for total PUFA and *n*-3 PUFA, which were an estimated 23 (95 % CI 11, 35) % and 47 (95 % CI 10, 84) % higher in organic meat, respectively. However, for these and many other composition parameters, for which meta-analyses found significant differences, heterogeneity was high, and this could be explained by differences between animal species/meat types. Evidence from controlled experimental studies indicates that the high grazing/forage-based diets prescribed under organic farming standards may be the main reason for differences in FA profiles. Further studies are required to enable meta-analyses for a wider range of parameters (e.g. antioxidant, vitamin and mineral concentrations) and to improve both precision and consistency of results for FA profiles for all species. Potential impacts of composition differences on human health are discussed.

The demand for organic meat products has increased steadily over the last 20 years^(^ ^1^ ^)^. A major driver for this increase has been consumer perception that organic livestock products typically contain higher concentrations of nutritionally desirable compounds, therefore making them ‘healthier’^(^ ^2^ ^,^ ^3^ ^)^. However, there is still considerable scientific uncertainty over whether, and to what extent, organic production standards result in significant and nutritionally relevant changes in food quality^(^ ^3^ ^–^ ^6^ ^)^.

In Western European diets, meat is an important source of protein, essential fatty acids (FA), minerals (e.g. Fe, Zn, Se, Cu) and vitamins (e.g. vitamin A, vitamin B_1_, B_6_ and B_12_, riboflavin, folate, niacin, pantothenic acid)^(^ ^7^ ^)^. Over the last 20 years, an increasing number of scientific studies have compared concentrations of nutritionally relevant compounds in meat from organic and conventional livestock production systems. Most comparative studies have reported data on meat fat composition, whereas there are limited published data on mineral and vitamin concentrations^(^ ^4^ ^,^ ^8^ ^,^ ^9^ ^)^.

The SFA in meat, in particular lauric (12 : 0), myristic (14 : 0) and palmitic (16 : 0) acids, are widely considered to have negative effects on human health, as they are linked to an increased risk of CVD in humans^(^ ^10^ ^)^, although this is not universally accepted^(^ ^11^ ^–^ ^13^ ^)^.

In contrast, a range of PUFA found in meat are thought to reduce the risk of CVD^(^ ^14^ ^)^. This includes linoleic acid (LA; the main *n*-6 PUFA found in meat), *α*-linolenic acid (ALA, the main *n*-3 PUFA found in meat) and, in particular, the very long-chain (VLC, ≥C20) *n*-3 PUFA EPA, docosapentaenoic acid (DPA) and DHA. Both LA and ALA are known to reduce LDL production and to enhance its clearance^(^ ^14^ ^)^, whereas VLC *n*-3 PUFA are also shown to reduce arrhythmias, blood pressure, platelet sensitivity, inflammation and serum TAG concentrations^(^ ^15^ ^,^ ^16^ ^)^. There is also evidence of other health benefits from increasing VLC *n*-3 PUFA (especially DHA) intakes, including improved fetal brain development, delayed decline in cognitive function in elderly men and reduced risk of dementia (especially Alzheimer’s disease)^(^ ^17^ ^)^.

Although LA may reduce CVD risk, intakes associated with typical Western diets are thought to be too high^(^ ^18^ ^)^. This is mainly because LA is the precursor of the pro-inflammatory *n*-6 PUFA arachidonic acid (AA). In contrast, *n*-3 FA are considered to have an anti-inflammatory effect^(^ ^15^ ^,^ ^16^ ^,^ ^19^ ^,^ ^20^ ^)^. In addition, high dietary *n*-6 PUFA intakes have been linked to an increased risk of other chronic diseases including certain cancers, inflammatory, autoimmune and CVD^(^ ^16^ ^,^ ^21^ ^)^ as well as shown to stimulate adipogenesis (and thereby the risk of obesity) to a greater extent compared with *n*-3 FA^(^ ^22^ ^)^. Excessive LA intakes during pregnancy and in the first few years of life have been linked to a range of neurodevelopmental deficits and abnormalities in children^(^ ^23^ ^)^. LA may also reduce the rate of conversion of ALA to VLC *n*-3 PUFA in humans, because ALA and LA compete for Δ6 desaturase enzyme activity^(^ ^24^ ^)^.

Systematic literature reviews and meta-analyses of comparative composition data for (1) crops, (2) milk and (3) milk, eggs and meat together have been published^(^ ^4^ ^,^ ^5^ ^,^ ^8^ ^,^ ^9^ ^,^ ^25^ ^)^, but there are no published meta-analyses in which the composition of organic and non-organic meat is compared. In this study, we report the results of a systematic review of the literature published before March 2014 and meta-analyses of data designed to quantify nutritionally relevant composition parameters in organic and conventional meat products.

For meta-analyses and interpreting the overall strength of evidence, total PUFA and *n*-3 PUFA concentrations were considered the primary outcome, because they are considered to be most closely linked to potential human health outcomes (see above). A range of other nutritionally relevant meat fat parameters were considered secondary outcomes.

Where possible, additional meta-analyses were carried out; these included some individual FA, the thrombogenicity and atherogenicity indices (which might be used to compare the overall CVD risk associated with different meat FA profiles^(^ ^19^ ^,^ ^26^ ^,^ ^27^ ^)^) and a range of other composition parameters (e.g. total protein, minerals, toxic metals), but for many of these only a small number of data pairs (*n* 3–5) were available. We were therefore unable to carry out meaningful meta-analyses for nutritionally relevant minerals, antioxidants and vitamins found in meat.

Previous meta-analyses of composition differences between organic and conventional foods (i.e. for crops, and milk and dairy products) have used variable inclusion criteria, data extraction and synthesis methods^(^ ^4^ ^,^ ^5^ ^,^ ^8^ ^,^ ^9^ ^,^ ^25^ ^)^. In the present study, sensitivity analyses designed to identify the effect of using different inclusion criteria, extraction and analysis methods were therefore performed to assess the consistency of findings. Results are discussed in the context of known information on (1) the effects of livestock management practices (especially feeding regimens) and breed choice on meat composition and (2) potential health impacts of composition differences between organic and non-organic meat.

## Methods

### Data acquisition: literature search strategy and inclusion criteria

The systematic review methods are described in a previously published meta-analysis by Barański *et al.* ^(^ ^25^ ^)^ focused on identifying composition differences between organic and conventional crops. The methods were based on a more detailed protocol for systematic reviews of composition differences published by Brandt *et al*.^(^ ^28^ ^)^. However, the protocols used in this study and by Barański *et al.* ^(^ ^25^ ^)^ differed from the detailed protocol published by Brandt *et al.* ^(^ ^28^ ^)^, notably in the emphasis on weighted meta-analysis (WM) rather than unweighted meta-analysis (UM), which had previously been recommended by Brandt *et al*.^(^ ^5^ ^,^ ^28^ ^)^ and Dangour *et al.* ^(^ ^4^ ^)^.

**Fig. 1:**
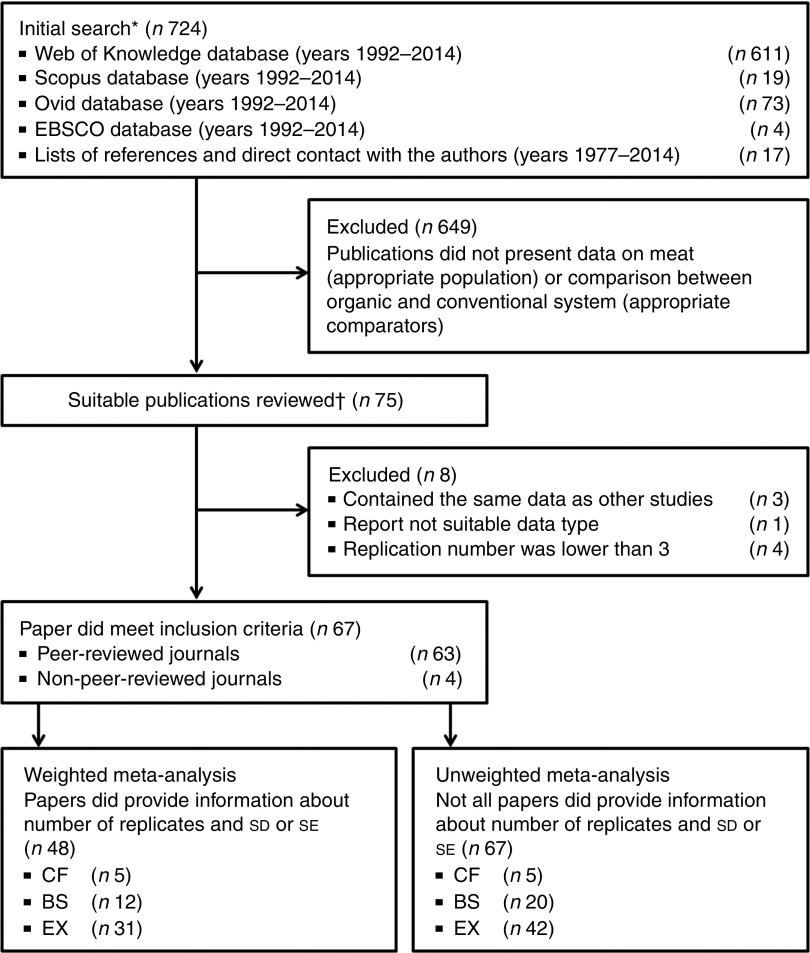
Summary of the search and selection protocols used to identify papers included in the meta-analyses. EBSCO, Elton B. Stephens Company; CF, comparison of matched farms; BS, basket studies; EX, controlled experiments. * Review carried out by one reviewer. † Data extraction carried out by two reviewers.

Relevant publications were identified through an initial search of the literature in the Web of Knowledge, Scopus, Ovid and EBSCO, Elton B. Stephens Company (EBSCO) databases using the following search terms: ‘organic* or ecologic* or biodynamic*’, ‘conventional* or integrated’ and ‘livestock or meat or pork or beef or poultry or chicken or turkey or lamb or goat or rabbit’ (Fig. 1).

Papers in all languages, published in peer-reviewed and non-peer-reviewed journals, and reporting data on both desirable and undesirable compositional parameters, were considered relevant for inclusion in the meta-analyses. The search was restricted to the period between 1992 (the year when legally binding organic farming regulations were first introduced in the European Union (EU)) and the end of the project in March 2014 and provided 707 references. An additional seventeen publications were found by studying lists of references or directly contacting authors of published papers and reviews identified in the initial literature search (Fig. 1).

The abstracts of all publications were then examined to determine whether they contained original data obtained by comparing composition parameters in organic and conventional beef, lamb or goat meat, pork, poultry or rabbit meat. This identified seventy-five suitable publications; of these, eight were subsequently rejected, because they did not report suitable data sets or contained the same data as other papers.

Data sets were deemed suitable if data for at least one meat composition parameter were reported. As a result, sixty-seven publications (sixty-three peer-reviewed) were selected for data extraction (sixteen on beef, sixteen on lamb and goat meat, fourteen on pork, seventeen on chicken meat, three on rabbit meat and one on non-specified meats).

Data from forty-eight publications (forty-seven peer-reviewed) fulfilled the criteria for inclusion in the random effects WM and UM. The additional nineteen publications (sixteen peer-reviewed) fulfilled the criteria for inclusion in the UM only.

This represents a significantly greater evidence base compared with a previous systematic review of comparative studies by Dangour *et al.* ^(^ ^4^ ^)^ that (1) was based on eleven publications reporting meat composition data, (2) pooled meat, egg and milk/dairy product composition data and (3) used unweighted, under-powered analytical methods only. All publications included in this previous review were also used in the random effects WM reported in this study.

A Preferred Reporting Items for Systematic Reviews and Meta-Analyses flow diagram illustrates the search and study inclusion strategies (Fig. 1). Eligibility assessment was performed by two independent reviewers, with discrepancies resolved by consensus and reference to a third reviewer when necessary.

### Data extraction

Data were extracted from three types of studies: (1) comparisons of matched farms (CF), farm surveys in which meat was obtained from organic and conventional farms in the same country or region; (2) basket studies (BS), retail product surveys in which organic and conventional meats were obtained from retail outlets; and (3) controlled experiments (EX) in which meat was obtained from experimental animals reared according to organic or conventional farming standards/protocols. Data from the three study types were deemed relevant for meta-analysis if the authors stated that (1) organic farms included in farm surveys were using organic farming methods, (2) organic products collected in retail surveys were labelled as organic and (3) animals from organically reared herds used in controlled experiments were managed according to organic farming standards, even if animals and land used for ‘organic treatments’ in experiments were not organically certified.

Several studies compared more than one organic or conventional system or treatment – for example, additional conventional systems were described as ‘intensive’ or ‘free range’. In such cases, a pragmatic choice was made to compare the organic with the standard conventional (non-organic) comparator. Standard systems were identified as closest to the typical, contemporary organic/conventional farming system, as recommended by Brandt *et al.* ^(^ ^5^ ^)^. Full references of the publications and summary descriptions of studies included in the meta-analyses are given in the online Supplementary Tables S1–S3.

Information and data were extracted from all selected publications and compiled in a Microsoft Access database. The database is freely available on the Newcastle University website (http://research.ncl.ac.uk/nefg/QOF) for use and scrutiny by others. A list of the information extracted from publications and recorded in the database is given in the online Supplementary Table S4.

Data reported as numerical values in the text or tables were copied directly into the database. Results only published in graphical form were enlarged, printed, measured (using a ruler) and then entered into the database as previously described^(^ ^5^ ^)^.

Data reported in the same publication for different animal species, products, study types, countries and outcomes were treated as independent effects. However, data extracted from the same publication for (1) different years, (2) different regions, retail outlets or brands in the same country or (3) multiple time points within the same sampling year were averaged before use in the meta-analysis.

Two independent reviewers assessed publications for eligibility and extracted data. Discrepancies were detected for approximately 4 % of the data, and in these cases extraction was repeated following discussion.

Study characteristics, summaries of methods used for sensitivity analyses and ancillary information are given in the online Supplementary Table S2–S7. They include information on (1) the number of papers from different countries and publication years used in the meta-analyses (see online Supplementary Fig. S1 and S2), (2) study type, location, meat product, animal group and information regarding FA analysis methods used in different studies (online Supplementary Table S2), (3) production system information for studies with more than two systems (online Supplementary Table S3), (4) the type of information extracted from papers (online Supplementary Table S4), (5) data handling and inclusion criteria and meta-analysis methods used in sensitivity analyses (online Supplementary Table S5), (6) the list of composition parameters included in the meta-analyses (online Supplementary Table S6) and (7) the list of composition parameters for which meta-analyses were not possible (*n*<3) (online Supplementary Table S7).

**Fig. 2:**
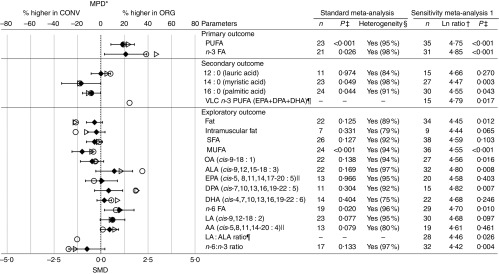
Results of the standard weighted meta-analysis and sensitivity analysis 1 for fat composition of meat (data for all animal groups included in the same analysis). * Numerical values for mean percentage difference (MPD) and 95 % CI are given in the online Supplementary Table S9. † Ln ratio=ln (ORG/CONV×100 %). ‡ *P* value<0·05 indicates a significant difference between organic samples (ORG) and conventional samples (CONV). § Heterogeneity and the *I* ^2^ statistic. || Outlying data points (where the MPD between ORG and CONV was more than fifty times greater than the mean value including the outliers) were removed. ¶ Calculated based on published fatty acids (FA) composition data. *n*, number of data points included in meta-analyses; VLC *n*-3 PUFA, very long-chain *n*-3 PUFA; DPA, docosapentaenoic acid; OA, oleic acid; ALA, *α*-linolenic acid; LA, linoleic acid; AA, arachidonic acid; SMD, standardised mean difference; ○, MPD calculated using data included in standard unweighted meta-analyses; ▷, MPD calculated using data include in standard weighted meta-analysis; ◆, SMD with 95 % CI represented by horizontal bars.

**Fig. 3:**
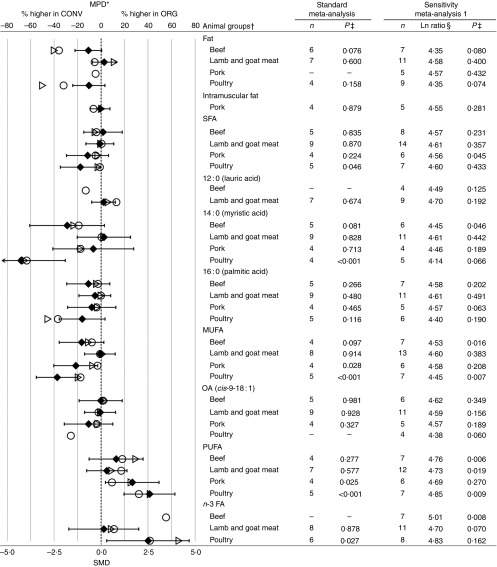
Results of the standard weighted meta-analysis and sensitivity analysis 1 for different animal groups for fat composition in meat. * Numerical values for mean percentage difference (MPD) and 95 % CI are given in the online Supplementary Table S10. † For parameters for which *n*≤3 for specific animal group, results obtained in the meta-analyses are not shown. ‡ Ln ratio=ln (ORG/CONV×100 %). § *P* value <0·05 indicates a significant difference between organic samples (ORG) and conventional samples (CONV). *n*, number of data points included in the meta-analyses; OA, oleic acid; FA, fatty acids; SMD, standardised mean difference; ○, MPD calculated using data included in standard unweighted meta-analyses; ▷, MPD calculated using data include in standard weighted meta-analysis; ◆, SMD with 95 % CI represented by horizontal bars.

**Fig. 4:**
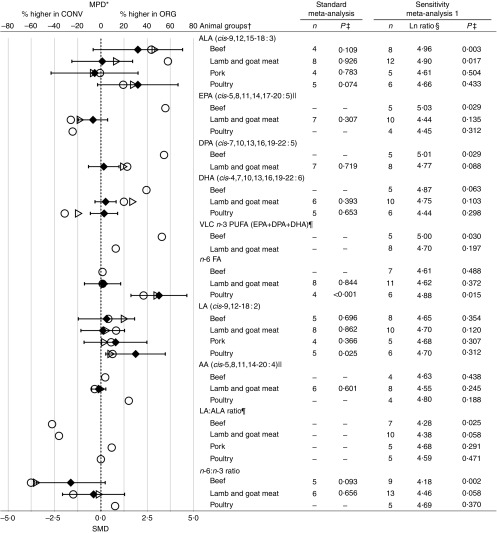
Results of the standard weighted meta-analysis and sensitivity analysis 1 for different animal groups for fat composition in meat. * Numerical values for mean percentage difference (MPD) and 95 % CI are given in the online Supplementary Table S10. † For parameters for which *n*≤3 for specific animal group, results obtained in the meta-analyses are not shown. ‡ Ln ratio=ln (ORG/CONV×100 %). § *P* value<0·05 indicates a significant difference between organic samples (ORG) and conventional samples (CONV). || Outlying data points (where the MPD between ORG and CONV was more than fifty times greater than the mean value including the outliers) were removed. ¶ Calculated based on published FA composition data. *n*, number of data points included in the meta-analyses; ALA, *α*-linolenic acid; DPA, docosapentaenoic acid; VLC *n*-3 PUFA, very long-chain *n*-3 PUFA; FA, fatty acids; LA, linoleic acid; AA, arachidonic acid; SMD, standardised mean difference; ○, MPD calculated using data included in standard unweighted meta-analyses; ▷, MPD calculated using data include in standard weighted meta-analysis; ◆, SMD with 95 % CI represented by horizontal bars.

The online Supplementary Table S8 summarises the basic statistics on the number of studies, individual comparisons, organic and conventional samples sizes and comparisons showing statistically or numerically higher concentrations in organic or conventional meat for the composition parameters included in Fig. 2–4.

### Meta-analyses

In total, six analyses were undertaken (online Supplementary Table S5). The standard WM and UM sensitivity analysis 1 compared data only from pragmatically chosen standard organic and conventional systems. Fig. 2–4 show the pooled effects obtained using standard random-effects meta-analysis weighted by inverse variance and a common random-effects variance component and UM of differences in means. The standard WM protocol is the primary analysis, but it is useful to augment the results with UM (particularly to explore the impact of including data from the studies that do not report measures of variance, and thus a wider range of studies).

Four additional sensitivity analyses were carried out. Two analyses (sensitivity analysis 2 and 3) were designed to identify whether exclusion of data for comparisons with non-standard organic or conventional systems would affect the results of the meta-analyses; in these analyses, comparative data for all organic and conventional production systems reported by authors were included (see online Supplementary Table S3). In sensitivity analysis 4, we explored the effect of excluding the 20 % of studies with the least precise treatment effects from the WM.

The suitability of analytical methods used in studies contributing data for WM and UM of FA profiles was assessed, and for most studies it was considered to be scientifically sound for comparison of relative differences between organic and conventional meat samples. Most studies used established GC-based protocols and described methods in sufficient detail. Seven studies may be classified as being of lower quality, which included two studies that used an near IR-spectroscopy method calibrated with GC data (ID209 and ID355) and five studies that provided only brief descriptions of the methods used (ID159, ID407, ID560, ID570 and ID606). When these studies were excluded from the meta-analyses (sensitivity analysis 5), broadly similar results were obtained. However, as the laboratories that carried out these five studies were reputable institutions and to minimise publication bias, we included data from all studies in the standard WM reported here. The results of sensitivity analyses 2–5 are available in the Appendix on the Newcastle University website (http://research.ncl.ac.uk/nefg/QOF).

Effect sizes for all WM were based on standardised mean differences (SMD) as recommended for studies that include data obtained by measuring the same parameters on different scales^(^ ^29^ ^,^ ^30^ ^)^.

Both WM and UM were carried out using the R statistical programming environment^(^ ^31^ ^)^. WM, with the SMD as the basic response variable, were carried out using standard methods and the open-source ‘metafor’ statistical package^(^ ^32^ ^–^ ^35^ ^)^. A detailed description of the methods and calculations is provided in the ‘Additional Methods and Results’ in the Supplementary Information available online.

A positive SMD value indicates that mean concentrations of the observed compound were greater in the organic meat samples, whereas a negative SMD indicates that mean concentrations were higher in conventional (non-organic) samples. The statistical significance of a reported effect size (i.e. SMD_tot_) and CI were estimated based on standard methods^(^ ^36^ ^)^ using ‘metafor’^(^ ^32^ ^)^. The influence of potential moderators, particularly (1) meat type (beef, lamb and goat, pork, rabbit or chicken meat) and (2) study type (CF, EX, BS), were additionally tested using mixed-effect models^(^ ^37^ ^)^ and subgroup analyses (Fig. 3 and 4, and online Supplementary Fig. S3–S5).

We carried out tests of homogeneity (*Q* statistics and *I* ^2^ statistics) on all summary effect sizes. Homogeneity was indicated when *I* ^2^ was <25 % and the *P* value for the *Q* statistics was >0·010. Funnel plots, Egger’s tests of funnel plot asymmetry and fail-safe number tests were used to assess publication bias^(^ ^38^ ^)^ (see online Supplementary Table S13 for further information).

In the UM, the significance and magnitude of differences in contents of the compounds were calculated using a resampling method, where the ratio of organic means/conventional means (*X̅*_O_/*X̅*_C_) expressed as a percentage was ln-transformed and values used to determine if the arithmetic average of the ln-transformed ratios was significantly greater than ln(100)^(^ ^39^ ^)^. Reported *P* values were derived from Fisher’s one-sample randomisation test^(^ ^40^ ^)^, and a *P*<0·05 was considered to be statistically significant.

There are currently very few publications that report comparative data for thrombogenicity and/or atherogenicity indices, and all provide information on lamb and goat meat only. However, a much larger number of publications covering a range of meat types reported sufficient data for individual FA/groups of FA to calculate the two indices. On the basis of those reported data, we calculated values of the thrombogenicity and atherogenicity indices as follows:
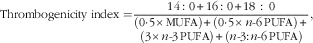


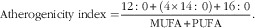



For the thrombogenicity index fifteen data points (three for beef, seven for lamb and goat meat, two for pork and three for chicken meat) and for the atherogenicity index thirteen data points (three for beef, eight for lamb and goat meat, one for pork and one for rabbit meat) were available. We carried out separate meta-analyses for the published and calculated estimates of the two indices (Fig. 2 and 4; online Supplementary Tables S9–S11 and Fig. S5). For all parameters (thrombogenicity index, atherogenicity index, total VLC *n*-3 PUFA, LA:ALA ratio) that were calculated based on published information it was only possible to carry out UM (Fig. 2–4), as measures of variance were not available.

Forest plots were constructed to show pooled SMD and corresponding 95 % CI for all compositional parameters investigated. Additional forest plots were presented for selected results to illustrate heterogeneity between subgroups based on types of meat (see online Supplementary Fig. S6–S35).

The mean percentage difference (MPD) was calculated for all parameters for which statistically significant effects were detected by either WM or UM. This was carried out to facilitate value judgements regarding the biological importance of the relative effect magnitudes using the calculations described by Barański *et al.* ^(^ ^25^ ^)^.

We calculated MPD for data pairs included in both the WM and the UM in order to estimate the impact of excluding data for which no measures of variance were reported on the magnitude of difference. As the MPD can be expressed as ‘% higher’ in conventional or organic meat, they provide estimates for the magnitude of composition differences that are easier to relate to existing information on potential health impacts of changing dietary intakes for individual or groups of compounds than the SMD values. The 95 % CI for MPD were estimated using a standard method^(^ ^36^ ^)^.

An overall assessment of the strength of evidence was made using an adaptation of the Grading of Recommendations Assessment, Development and Evaluation (GRADE)^(^ ^41^ ^)^ system (Table 1).

**Table 1:** Grading of Recommendations Assessments, Development and Evaluation (GRADE) assessment of the strength of evidence for standard weighted meta-analysis for parameters shown in Fig. 2 (Standardised mean difference (SMD) values and 95 % confidence intervals)

Parameters	SMD	95 % CI	Effect magnitude*	Inconsistency†	Precision‡	Publication bias§	Overall reliability||
Fat composition							
Fat	−0·35	−0·80, 0·10	Small	Low	Poor	Medium	Low
Intramuscular fat	−0·25	−0·74, 0·25	Small	Low	Moderate	Strong	Low
SFA	−0·35	−0·79, 0·10	Small	Medium	Poor	No	Moderate
12 : 0 (lauric acid)	−0·01	−0·55, 0·53	Small	Low	High	Medium	Moderate
14 : 0 (myristic acid)	−1·02	−2·09, 0·04	Moderate	High	Poor	Strong	Very low
16 : 0 (palmitic acid)	−0·47	−0·96, 0·02	Small	Low	Poor	Strong	Very low
MUFA	−1·01	−1·57, −0·45	Moderate	High	Moderate	Medium	Moderate
OA (*cis*-9-18 : 1)	−0·48	−1·12, 0·16	Small	Low	Poor	Medium	Low
PUFA	1·15	0·51, 1·80	Moderate	High	Moderate	Medium	Moderate
*n-*3 FA	1·31	0·16, 2·45	Moderate	Medium	Poor	Strong	Low
ALA (*cis*-9,12,15-18 : 3)	0·73	−0·27, 1·73	Small	High	Poor	Strong	Very low
EPA (*cis*-5,8,11,14,17-20 : 5)¶	0·02	−0·85, 0·90	Small	High	Moderate	Strong	Very low
DPA (*cis*-7,10,13,16,19-22 : 5)	0·40	−0·36, 1·17	Small	Low	Moderate	Strong	Low
DHA (*cis*-4,7,10,13,16,19-22 : 6)	0·22	−0·17, 0·61	Small	Medium	High	Strong	Low
VLC *n-*3 PUFA (EPA+DPA+DHA)**	–	–	–	–	–	–	–
*n-*6 FA	0·97	0·15, 1·78	Moderate	High	Moderate	Strong	Low
LA (*cis*-9,12-18 : 2)	0·65	−0·01, 1·30	Small	Medium	Poor	Medium	Low
AA (*cis*-5,8,11,14-20 : 4)¶	0·45	−0·05, 0·94	Small	Medium	Poor	Medium	Low
LA:ALA ratio**	–	–	–	–	–	–	–
*n-*6:*n-*3 ratio	−0·75	−1·72, 0·23	Moderate	High	Poor	Medium	Low

OA, oleic acid; FA, fatty acids; ALA, *α*-linolenic acid; DPA, docosapentaenoic acid; VLC *n-*3 PUFA, very long-chain *n-*3 PUFA; LA, linoleic acid; AA, arachidonic acid.

* Study quality was considered low because of high risks of bias and potential for confounding. However, we considered large effects to mitigate this *sensu* GRADE; large effects were defined as >20 %, moderate effects 10–20 and small <10 %.

† Inconsistency was based on the measure of heterogeneity and consistency of effect direction *sensu* GRADE.

‡ Precision was based on the width of the pooled effect CI and the extent of overlap in substantive interpretation of effect magnitude *sensu* GRADE.

§ Publication bias was assessed using visual inspection of funnel plots, the Egger’s test, two-tests of fail-safe *N* and trim and fill (see online Supplementary Table S13). Overall publication bias was considered high when indicated by two or more methods, moderate when indicated by one method and low when no methods suggested publication bias.

|| Overall quality of evidence was then assessed across domains as in standard GRADE appraisal; high when there was very high confidence that the true effect lies close to that of the estimate, moderate when there was moderately confidence in effect estimate and the true effect is likely to be close to the estimate but there is a possibility that it is substantially different, low when the confidence in the effect estimate was limited and the true effect may be substantially different from the estimate, very low when there was very little confidence in the effect estimate and the true effect is likely to be substantially different from the estimate.

¶ Outlying data pairs (where the mean percentage difference between organic and conventional meat samples was over fifty times greater than the mean value including outliers) were removed.

** Calculated based on published FA composition data.

### Estimation of fatty acid intakes

Intakes were estimated for FA parameters for which WM based on pooled data from all meat types had detected significant differences between organic and conventional meat. All FA data extracted from the original publications were converted into a common unit (g/100 g total FA esters). These values were then used to calculate mean FA concentrations in different meat types. These means were then used to calculate total FA intakes from organic and conventional meats using (1) published data on fat consumption from different meat types in the EU^(^ ^42^ ^)^ and (2) for mean concentrations of total FA esters in organic and conventional meats (Fig. 3 and 4). MPD in FA intakes between organic and conventional meats was then calculated (see Table 2). It should be pointed out that the European fat consumption data were based on means from all EU countries, whereas means for FA concentrations in organic and conventional meats were based on published data from eight EU countries (Germany, Denmark, Spain, France, UK, Italy, Poland, Sweden; contributing approximately 70 % of data) and seven countries from outside the EU (Switzerland, Brazil, Republic of Korea, Turkey, Taiwan, Province of China, USA, Uruguay). Estimates of FA intakes for specific countries were not possible owing to a lack of published data (comparative studies for all different meat types were not available for any one country).

**Table 2:** Estimated fatty acids (mg/person per d) intake from organic (ORG) and conventional (CONV) meat based on FAO’s fat supply quantity data^(^ ^42^ ^)^ for bovine meat, pig meat, sheep and goat meat and poultry meat in the European Union, calculated using the data included in the unweighted meta-analysis shown in Fig. 2

	Consumption associated with														
	Beef*			Lamb and goat meat†			Pork‡			Chicken meat§			Total meat		
Parameters	ORG	CONV	MPD	ORG	CONV	MPD	ORG	CONV	MPD	ORG	CONV	MPD	ORG	CONV	MPD
SFA	1518	1507	1	527	528	0	6648	6868	−3	1408	1419	−1	10 100	10 322	−2
14 : 0 (myristic acid)	59	66	−12	60	61	−2	217	252	−16	27	41	−50	363	420	−16
16 : 0 (palmitic acid)	709	715	−1	252	254	−1	4238	4368	−3	993	999	−1	6191	6337	−2
MUFA	1307	1395	−7	406	414	−2	8229	8417	−2	1587	1858	−17	11 528	12 083	−5
PUFA	525	455	15	142	132	8	2930	2561	14	1482	1200	24	5080	4348	17
* n-*3 PUFA	128	78	64	41	40	2	419	360	16	161	136	19	748	613	22
* n-*6 PUFA	290	277	5	94	95	−1	4400	3637	21	1396	1100	27	6180	5110	21

MPD, mean percentage difference.

* Calculated assuming an average fat consumption from bovine meat of 3·5 g/person per d.

† Calculated assuming an average fat consumption from sheep and goat meat of 1·2 g/person per d.

‡ Calculated assuming an average fat consumption from pig meat of 19·1 g/person per d.

§ Calculated assuming an average fat consumption from poultry meat of 4·7 g/person per d.

## Results

### Characteristics of studies and data included in the meta-analyses

The WM and UM were based on data from sixty-three peer-reviewed papers and four non-peer-reviewed studies, including publications reporting farm surveys (five papers), controlled experiments (forty-two papers) and BS (twenty papers).

Most of the eligible studies were from Europe, mainly from Spain, UK, Italy, Sweden, Poland and Germany, with most of the others coming from the USA and Brazil (online Supplementary Table S2 and Fig. S2). Publications reported data on 373 different composition parameters, but the majority of studies (thirty-nine papers) focused on meat fat composition parameters (online Supplementary Tables S6 and S7). In contrast, relatively few studies (thirteen papers) reported data on mineral nutrients, toxic metals and/or other composition parameters. Meta-analyses were carried out on 122 meat-quality parameters (online Supplementary Tables S6 and S7).

### Composition of organic and conventional meat products

#### Fat composition

When data for all meat types were analysed together, WM detected significant differences in FA profiles between organic and conventional meat (Fig. 2). Organic meat had similar SFA, lower MUFA and higher PUFA concentrations compared with conventional meat. The MPD (calculated based on data used for the WM) were −8 (95 % CI −13, −4) % for MUFA and 23 (95 % CI 11, 35) % for PUFA, respectively (Fig. 2 and online Supplementary Table S9).

When data for different meat types were analysed separately, no differences in SFA were detected for beef, lamb and goat meat and pork, but WM detected slightly but significantly lower SFA concentrations in organic chicken meat (Fig. 3 and online Supplementary Fig. S9). However, it should be noted that only five individual studies were available for WM of SFA contents in chicken meat and that results differed between studies and/or countries/regions. Three studies (from the UK and Italy) reported no significant difference, whereas two others (from the Republic of Korea and the USA) reported significantly lower SFA concentrations in organic chicken meat (online Supplementary Table S9).

For MUFA, WM detected significantly lower concentrations for pork and chicken only (Fig. 3 and online Supplementary Fig. S14). However, it should be noted that only three and five individual studies were available for WM of MUFA contents in pork and chicken meat, respectively. For pork, results differed between studies and/or countries/regions; one study (from Poland) reported no significant difference, and two (from the Republic of Korea and Sweden) studies reported significantly lower MUFA concentrations in organic meat (online Supplementary Table S14). For chicken meat, all five studies (from the UK, Italy, Republic of Korea and the USA) reported significantly lower MUFA concentrations in organic chicken meat (online Supplementary Table S14).

For PUFA, significantly higher concentrations were detected for pork and chicken meat, but not for beef and lamb and goat meat (Fig. 3 and online Supplementary Fig. S19). However, it should be noted that only four and five individual studies were available for WM of PUFA contents in pork and chicken meat, respectively, and for both pork and chicken meat results differed between studies and/or countries/regions (online Supplementary Table S19). For pork, one study (from Sweden) reported no significant differences and two studies (from the Republic of Korea and Poland) reported significantly higher PUFA concentrations in organic meat. For chicken meat, two studies (from the UK and Italy) reported no significant differences, whereas three studies (from the UK, Republic of Korea and the USA) reported significantly higher PUFA in organic chicken meat (online Supplementary Table S19).

When data for all meat types were analysed together, WM identified significantly lower concentrations of the SFA myristic acid (14 : 0) and palmitic acid (16 : 0) in organic compared with conventional meat. The MPD were −18 (95 % CI −32, −5) % for myristic acid and −11 (95 % CI −28, 5) % for palmitic acid (Fig. 2).

When data for different meat types were analysed separately, WM detected significantly lower 14 : 0 concentrations for organic chicken meat only (Fig. 3 and online Supplementary Fig. S11). However, it should be noted that only four studies were available for WM of PUFA in chicken meat and that results differed between studies and/or countries/regions; two studies (both from the UK) reported no significant difference, whereas two others studies (from the UK and Republic of Korea) reported significantly lower 14 : 0 concentrations in organic chicken meat (online Supplementary Fig. S11).

For 16 : 0, WM detected no significant difference for all individual meat types (Fig. 3 and online Supplementary Fig. S12).

When data for all meat types were analysed together, WM detected significantly higher *n*-3 and *n*-6 concentrations in organic compared with conventional meat (Fig. 2). The MPD (calculated based on the data used for the WM) were 47 (95 % CI 10, 84) % for *n*-3 PUFA and 16 (95 % CI 2, 31) % for *n*-6 PUFA, respectively.

When data for different meat types were analysed separately, WM detected significantly higher concentrations of total *n*-3 PUFA in organic chicken meat only (Fig. 3 and online Supplementary Fig. S20). However, it should be noted that only six studies were available for WM of *n*-3 PUFA in chicken meat and that results differed between studies and/or countries/regions; two studies (both from the UK) reported no significant difference, whereas four other studies (from the UK, Italy, Republic of Korea and the USA) reported significantly higher *n*-3 PUFA in organic chicken meat (online Supplementary Fig. S11).

WM detected no significant differences for CLA, EPA, DPA and DHA, a range of other SFA, MUFA and PUFA and the *n*-6:*n*-3 ratio (Fig. 2 and online Supplementary Table S12).

UM were carried out as ‘sensitivity analyses’ to estimate the extent to which an increase in the evidence base (inclusion of publications in which no measures of variance were reported) would identify additional composition differences. When data for different meat types were pooled, UM results were similar to those obtained by WM for total SFA, MUFA and PUFA and for *n*-3 PUFA, *n*-6 PUFA, 14 : 0 and 16 : 0 (Fig. 2). However, different to the WM, the UM-based sensitivity analyses also detected significant differences for a range of other fat composition parameters. Specifically, UM detected (1) lower total fat and oleic acid concentrations, (2) higher ALA, DPA and total VLC *n*-3 PUFA (EPA+DPA+DHA) concentrations, (3) a lower *n*-6:*n*-3 PUFA ratio and (4) a lower thrombogenicity index in organic meat (Fig. 2; online Supplementary Table S9).

For individual meat types, UM (sensitivity analysis 1) allowed comparisons for a wider range of composition parameters for all meat types and detected additional differences between organic and conventional meats (Fig. 3). This included (1) lower 14 : 0 and MUFA but higher PUFA, *n*-3 PUFA, EPA, DPA and total VLC *n*-3 PUFA concentrations in beef, (2) higher PUFA and ALA concentrations in lamb and goat meat and (3) lower SFA concentrations in organic pork (Fig. 3).

#### Estimation of fatty acid intakes from organic and conventional meats

Accurate comparisons of FA intakes between organic and conventional meats are currently not possible, due to (a) the contrasting pattern of total meat and types of meat (e.g. beef, lamb, pork, chicken meat) consumed in different countries and (b) lack of sufficient comparative data sets to estimate FA composition difference for specific countries. This makes it impossible to carry out country-specific intake estimates. Estimates of FA intakes were therefore calculated using published meat fat consumption data for the EU and mean FA composition data obtained from the systematic literature review. Moreover, intake estimates were only carried out for FA parameters for which relatively large data sets (*n*>20) were available and for which the WM had detected significant differences between organic and conventional meat (Table 2).

Intakes of total SFA and palmitic acid had similar numerical values, whereas values for myristic acid (14 : 0) were lower with organic meat consumption (Table 2). Larger differences in numerical values were found for beef (−12 %), pork (−16 %) and chicken (−50 %), and overall the intake of myristic acid was estimated to be 16 % lower based on average meat consumption pattern in the EU (Table 2).

Intakes of total MUFA with meat were estimated to be similar (−5 %) based on average meat consumption pattern in the EU (Table 2).

Larger numerical differences in intakes were calculated for total PUFA, *n*-3 PUFA and *n*-6 PUFA, which were all higher (by 17, 22 and 21 %, respectively) with organic meat consumption based on average meat consumption pattern in the EU (Table 2). However, there was considerable variation in the MPD calculated for intakes for different meat types (Table 2). Owing to the more limited data available, comparisons of intakes with organic and conventional meat are currently not possible for other FA parameters including VLC FA (EPA+DPA+DHA).

#### Minerals, toxic metals and other composition parameters

Compared with fat composition parameters, relatively few comparative data sets were available for meta-analyses of minerals (e.g. Fe, Se, Zn), toxic metals (e.g. As, Pb, Cd) and other composition parameters (including protein, vitamins and pesticides) in meat (online Supplementary Tables S6, S7 and S12). The meta-analyses detected some significant effects (e.g. for Cu), but these are not presented in detail in this study, because of the high level of uncertainty associated with meta-analysis results based on data from a very few studies.

### Effects of livestock species, study type and other sources of variation

Heterogeneity was high (*I* ^2^>75 %) for nearly all composition parameters, with *I* ^2^ ranging from 79 % for fat content to 98 % for 14 : 0 and *n*-3 PUFA concentrations (Fig. 2).

When meta-analysis results obtained from different study types (BS, CF and EX) were compared, broadly similar results were obtained for most of the composition parameters included in Fig. 2 (see online Supplementary Fig. S3–S5). However, there was considerable variation between results for different meat types or studies carried out in different countries (see Fig. 3 and online Supplementary Fig. S6–S35).

Non-weighted MPD were calculated to aid the biological interpretation of effect size magnitude where either the weighted or UM had identified statistically significant differences. For many parameters, MPD based on all the available data produced values very similar to those calculated using only data for which measures of variance were reported (those used for the WM; Fig. 2). However, for some parameters (*n*-3 PUFA, ALA), inclusion criteria had a moderate effect on the MPD.

In addition, when the calculated MPD were superimposed onto SMD results (with 95 % CI) at an appropriate scale (−80 to +80 for MPD and −3 to +3 for SMD), a reasonable match was observed, with MPD for most compounds being present within the 95 % CI for SMD (Fig. 2). However, for some parameters (fat, intramuscular fat, PUFA, *n*-3 PUFA, DPA and DHA), MPD were outside the 95 % CI of SMD, and therefore these should be seen as less reliable.

Sensitivity analyses designed to identify the effect of using different inclusion criteria and data-handling methods yielded results broadly similar to those of the standard weighted and UM for the composition parameters included in Fig. 2. The sensitivity analyses, designed to identify the effect of removing data from the 20 % of studies with least precise treatment effects also yielded broadly similar results, except for 14 : 0 and 16 : 0 and total *n*-3, for which non-significant differences were detected in some of the sensitivity analyses (see http://research.ncl.ac.uk/nefg/QOF for detailed results of the sensitivity analyses).

### Strength of evidence

The overall assessment of the strength of evidence based on WM using an adapted GRADE^(^ ^41^ ^)^ approach highlighted strong uncertainties, with the overall strength of evidence being very low or low for most composition parameters, and moderate overall reliability was found only for 12 : 0, SFA, MUFA and PUFA concentrations (Table 1).

In general, there were substantial issues with study quality and reporting measures of variance, which were not generally mitigated by large effects. Inconsistency was high and precision was low. Strong or medium funnel plot asymmetry consistent with publication biases was also apparent for many parameters (see online Supplementary Table S13). However, it is not possible to definitely attribute discrepancies between large precise studies and small imprecise studies to publication bias, which remains strongly suspected rather than detected where asymmetry is severe.

## Discussion

Results of the meta-analyses reported in this study indicate for the first time that there are significant and nutritionally meaningful composition differences between organic and non-organic meat. This contradicts the results of a previous literature review by Dangour *et al.* ^(^ ^4^ ^)^, which pooled comparative data for meat, eggs, milk and dairy products in their analyses and concluded that overall there are no significant composition differences between organic and conventional livestock products (meat, dairy products and eggs). However, results for specific parameters reported in this study were variable, and both previous reviews^(^ ^4^ ^,^ ^9^ ^)^ covering livestock products and the present study acknowledge serious deficiencies in the evidence, which result in considerable uncertainty. Plausible mechanistic explanations for the findings in this study are discussed below.

Meta-analysis results suggesting that certain organic meats (beef, lamb and pork) have higher concentrations of PUFA and *n*-3 PUFA are broadly consistent with results from controlled animal experiments that studied the effect of grazing or high-forage diets and the use of legume-rich forages (both of which are typically used in organic production) on meat quality^(^ ^43^ ^–^ ^45^ ^)^. However, it should be pointed out that (a) the evidence base for individual meat/types/livestock species was very small (usually between two and seven studies), (2) the meta-analyses did not detect significant differences for all meat types/livestock species and (3) that results for PUFA and *n*-3 PUFA varied between individual studies and studies carried out in different countries/regions. Other composition differences (e.g. the lower concentrations of 14 : 0 and 16 : 0 and higher concentrations of total *n*-6 PUFA in organic chicken meat) detected by meta-analyses may also be explained by differences in management practices between organic and conventional production systems^(^ ^46^ ^–^ ^48^ ^)^.

We therefore discuss below (1) current knowledge about the effects of management practices (especially feeding regimens) that may explain composition differences between organic and conventional meat, (2) the strength of evidence and potential reasons for the heterogeneity of the available data/evidence, (3) potential nutritional/health impacts of meat from organic and other grazing or high-forage livestock production systems, (4) the need for expanding the current evidence base available for meta-analysis and (5) the requirement for dietary intervention and/or cohort studies to quantify potential health impacts of organic meat consumption.

### Links between livestock management and meat composition/quality

Organic livestock production standards prescribe that livestock are to be reared outdoors for a part of the year, although the length of outdoor periods differs among regions and livestock species^(^ ^49^ ^–^ ^51^ ^)^. EU organic standards prescribe that (1) ruminants receive at least 60 % of total DM intake (DMI) from forage (from grazing, cut fresh forage or conserved forage such as silage or hay) and (2) pigs and poultry are provided with access to forage but intake levels are not specified^(^ ^49^ ^–^ ^51^ ^)^. For ruminants, organic regulations also prescribe that fresh forage intake is from grazing ‘when conditions allow’, and as a result the duration of grazing and the ratio of fresh:conserved forage in organic diets vary significantly between European regions, mainly due to differences in pedo-climatic and agronomic conditions^(^ ^48^ ^,^ ^52^ ^)^. Where organic pigs and poultry have access to grassland, this may also result in significant fresh forage intake, but in many regions organic pigs and poultry are fed conserved forage only^(^ ^46^ ^,^ ^47^ ^)^.

In contrast, in conventional beef, pork and poultry (and in some regions also lamb and goat) production, there has been a trend towards (1) reduced outdoor grazing or all-year-round housing and (2) reductions in both fresh and conserved forage intakes, but (3) increased use of concentrate feeds based on maize, other cereals, soya, other grain legumes and by-products from the food processing industry^(^ ^53^ ^–^ ^55^ ^)^.

#### Feeding regimens

A range of controlled animal experiments showed that high grazing/forage-based diets (similar to those prescribed under organic farming standards) reduce the total fat and/or nutritionally undesirable SFA (12 : 0, 14 : 0 and/or 16 : 0) content, while increasing concentrations of total PUFA, *n*-3 PUFA and VLC *n*-3 PUFA in meat, compared with concentrate-based diets (typical for intensive conventional farming systems)^(^ ^43^ ^–^ ^45^ ^)^. These results suggest that the relative divergence in feeding practices between the organic and conventional livestock sectors is a major driver for both the differences in meat FA composition between systems and the variability of results between countries/regions and individual studies detected in this study by meta-analyses.

Differences in meat composition (e.g. for *n*-3 PUFA) reported by controlled experimental studies are greater than the differences detected in this study between organic and conventional meat by meta-analysis, especially for ruminant livestock – for example, in beef production, a switch from grain- to grass-based finishing diets produced significant increases in total PUFA (45 %), total *n*-3 PUFA (>3-fold), ALA (>3-fold), EPA (>5-fold), DPA (>2-fold) and DHA (129 %) in the intramuscular fat in the *longissimus* muscle of beef, although it had no significant effect on total *n*-6 PUFA or LA concentrations^(^ ^44^ ^)^. In lamb production, a switch from grain- to grass-based finishing diets significant increased ALA (>2-fold), EPA (>2-fold), DPA (88 %) and DHA (100 %) in the intramuscular fat of pelvic limb muscle meat and decreased concentrations of LA (30 %) and AA (21 %)^(^ ^43^ ^)^. Although forage intakes in monogastric livestock are much lower than that in ruminants, free-range rearing of pigs with access to pasture grazing had significantly increased concentrations of PUFA, *n*-3 PUFA and ALA in the intramuscular fat when compared with meat from pigs reared indoors on standard concentrate-based diets^(^ ^45^ ^)^. However, the relative differences were smaller (<50 %) than those detected in studies with beef and lamb^(^ ^43^ ^,^ ^44^ ^)^. This suggests that there is considerable potential for both conventional and organic production to increase *n*-3 PUFA (including VLC *n*-3 concentrations) concentrations in beef, lamb and pork meat by further increasing grazing and the proportion of forage in livestock diets.

For poultry, there are limited data from controlled experimental studies that could potentially explain impacts of feeding regimens used in organic farming systems on meat quality, but access to forage may also at least partially explain the differences detected.

For pigs and poultry, differences in the type of concentrate (and in particular protein supplements) may also contribute to composition differences between organic and conventional meat, especially FA profiles – for example, although conventional pig and poultry production relies on chemically extracted soya meal (which has low levels of residual fat) to supply high-quality protein, organic standards only allow cold-pressed soya and other oil seed meals (which have a higher oil content). Moreover, on-farm-produced grain legumes (peas and beans) are more widely used as protein supplements in organic production, mainly because there is a need for a proportion of feed to be produced on farm because of the limited availability, high cost and ethical concerns about imported feeds^(^ ^46^ ^,^ ^47^ ^,^ ^55^ ^)^. The higher intake of soya oil (which has a high LA content) with cold-pressed soya meal may therefore explain the higher LA and *n*-6 concentrations detected by meta-analyses for organic chicken meat^(^ ^46^ ^,^ ^47^ ^)^.

#### Breed choice

The use of traditional and robust breeds/genotypes is often recommended by organic sector bodies and advisors. However, there is limited information on the relative differences in breed choice/breeding regimens between organic and conventional beef cattle, lamb, goat, pig and poultry production systems, and the papers used for meta-analyses provided no or insufficient data on the breeds used in the organic and conventional systems they compared.

It was therefore not possible to determine whether breed choice contributed significantly to the composition differences reported in this study. However, controlled experimental studies have demonstrated that breed choice does affect FA profiles of meat^(^ ^43^ ^–^ ^45^ ^)^.

#### Grassland/forage composition

The composition of grazing swards and conserved forages may also partially explain the differences between organic and conventional meat. Most importantly, forage-legume (e.g. clover, lucerne) or grass-legume mixtures are typically used in organic farming systems (where standards demand a specific proportion of fertility-building legume crops in the rotation). In contrast, pure grass or swards with a high proportion of grasses are more widely used in conventional/non-organic production systems, because the permitted use of mineral NPK fertilisers allows for higher DM yields per hectare compared with legume-grass mixtures. Evidence from studies comparing milk fat composition in extensive (grazing only) organic and non-organic dairy production systems (which use similar cross-breeds and grazing DMI) showed that organic milk (from cows grazing swards with a higher clover content) had significantly more *n*-3 PUFA, but lower CLA concentrations compared with milk from non-organic farms^(^ ^56^ ^,^ ^57^ ^)^. Similar impacts of legumes have also been reported for meat quality^(^ ^58^ ^)^: *longissimus dorsi* muscle from lambs grazing lucerne or red clover swards (more widely used in organic production systems) had significantly greater PUFA:SFA ratios and higher concentrations of both LA and ALA compared with lambs grazing grass swards.

#### Mineral supply and supplementation

Although some trends towards differences in mineral composition were detected by meta-analyses, these were based on a very limited evidence base and cannot be used to draw conclusions. However, they demonstrate the importance to carry out additional well-designed comparative studies, as organic and conventional livestock systems differ in a range of management practices that may affect the mineral composition of meat – for example, (1) conventional forage and grain crops often receive high inputs of mineral P fertilisers, a practice that has been linked to higher Cd concentrations in crops^(^ ^25^ ^,^ ^59^ ^)^, and (2) conventional livestock feeding regimens often use higher levels of mineral supplementation (e.g. more widespread use of Cu supplements in conventional pig production). In addition, Fe concentrations in meat may be increased by access to the outside or higher proportions of forage in the diet (as recommended by organic farming standards), as forages contain higher Fe concentrations than concentrate feeds, and it is well recognised that piglets with access to soil in their environment do not need Fe injections, routinely used in housed production systems^(^ ^60^ ^)^. In contrast, Cu deficiency in organically reared calves was linked to high forage and low concentrate intakes in one recent study^(^ ^61^ ^)^, and this may have been due to low Cu contents in soils used for forage production and/or the mineral supplements in the concentrate feed used for rearing calves in conventional systems.

### Strength of evidence and potential reasons for the heterogeneity of the available data/evidence

The high inconsistency and low precision of meta-analyses for many meat composition parameters may reflect both the paucity of information and variability associated with agri-production systems and especially livestock diets (see detailed description below). This highlights the need for (1) further well-designed studies delivering substantial additional primary data sets, (2) reporting of measures of variance in publications to facilitate inclusion in WM and (3) the establishment of registers of primary research^(^ ^29^ ^)^.

However, despite these uncertainties, there is a substantial body of evidence indicating that overall organic meat may have a more desirable FA profile than non-organic comparators. The consistency of association directions across the multiple outcomes and analyses mitigates some of the uncertainty associated with individual parameters from a decision-analytical perspective, but the currently available evidence requires cautious interpretation.

A major reason for the heterogeneity of the available data is likely to be the considerable variation in the intensity of both conventional and organic meat production systems. Non-organic production may range from intensive indoor production systems with high concentrate-based diets (>90 % of total DMI for pigs and poultry) to extensive outdoor grazing-based systems with high fresh and/or conserved forage (up to 100 % of total DMI) diets^(^ ^53^ ^–^ ^55^ ^)^. Although limited by the restrictions of organic farming regulations, there is also variation in production intensity within organic systems – for example, concentrate intakes may vary between 0 and 40 % of DMI for organic ruminant diets^(^ ^48^ ^,^ ^52^ ^)^. In addition, although organic ruminant diets are thought to be based on higher fresh forage from grazing and lower concentrate intakes in most European countries/regions, lower grazing-based DMI in organic, compared with extensive non-organic, have been documented for some ruminant livestock species in some regions of Europe – for example, dairy cattle in Southern Wales^(^ ^52^ ^,^ ^56^ ^)^ and dairy sheep and lamb meat production systems in Crete (Smaro Sotiraki, personal communication). This could explain why some studies showed a different trend (e.g. lower PUFA and *n*-3 FA in organic meat) to the overall results obtained by meta-analyses of pooled data or data for individual livestock species/meat types.

Other potential sources of heterogeneity are the range of different livestock species, meat types and countries and/or variable study designs and methodologies used in the studies from which data were extracted. In addition, data used in the meta-analyses were collected over a >20-year period and agronomic practices in both organic and conventional systems may have changed over time; this may also have contributed to heterogeneity.

As described in previous reviews focused on composition differences between organic and conventional crop-based foods^(^ ^5^ ^,^ ^25^ ^)^, pooling diverse information was necessary, because for most composition parameters the number of published studies available was insufficient to carry out separate meta-analyses for specific countries/regions, livestock species/meat types or study types. Consequently heterogeneity was high, although only PUFA appeared to be sensitive to variable inclusion criteria.

### Potential nutritional impacts of composition differences

#### Fat composition

The lower thrombogenicity index detected by UM for organic meat fat was due to both (1) lower concentrations of undesirable 14 : 0 and 16 : 0 (linked to an increased risk of CVD) and (2) higher concentrations of *n*-3 PUFA (linked to a decreased risk of CVD) found in organic meat. However, it should be pointed out that the thrombogenicity index as a predictor for CVD risk^(^ ^19^ ^)^ has not so far been validated in human dietary intervention or cohort studies. It is therefore currently not possible to estimate to what extent the changes in FA profiles and intakes may affect CVD risk (see also discussion below).

Increasing *n*-3 (especially VLC *n*-3) PUFA intake in human diets has been linked to a range of other health benefits in humans^(^ ^16^ ^,^ ^17^ ^,^ ^21^ ^–^ ^23^ ^)^. The 47 % higher total *n*-3 PUFA concentration detected by WM and estimated 17 % higher *n*-3 PUFA intake with organic meat could therefore be potentially beneficial, especially if intakes of VLC *n*-3 PUFA were increased. However, it is currently unclear whether there are systematic differences in VLC *n*-3 PUFA concentrations between organic and conventional meat, because there is currently insufficient data to carry out WM comparing VLC *n*-3 PUFA concentration in most individual meat types. UM were possible for a larger number of meat types and detected higher concentrations of VLC *n*-3 PUFA in beef, but not other meat types for which sufficient data were available.

Meat fat is an important source for VLC *n*-3 PUFA. Average consumption levels of meat have been estimated to be 240 and 340 g/d per person, with red meat at 184 and 270 g/d per person in Europe and the USA, respectively^(^ ^62^ ^)^. For the majority of North American and European consumers, meat is therefore the main dietary source for VLC *n*-3 PUFA, supplying up to an estimated 50 % of the recommended adequate intake. A priority for future studies should therefore be to substantially expand the evidence base for VLC *n*-3 PUFA for all meat types to allow accurate estimates of composition differences and dietary intakes with organic and conventional meat.

Although UM of pooled data for all meat types and beef indicated that organic production may reduce the LA:ALA and *n*-6:*n*-3 ratio, this cannot currently be confirmed by WM. These ratios may be nutritionally relevant, as additional VLC *n*-3 PUFA may be generated from dietary ALA, because humans can elongate ALA to produce longer-chain *n*-3 PUFA^(^ ^17^ ^,^ ^24^ ^,^ ^63^ ^–^ ^75^ ^)^. However, ALA to EPA conversion rates are thought to be low in humans and synthesis of DHA is very low, especially in men^(^ ^71^ ^)^. The proportion of ALA (the main *n*-3 in the human diet) converted to longer-chain *n*-3 FA in humans is thought to increase with decreasing LA:ALA ratios in the diet, as ALA and LA compete for Δ6 desaturase activity^(^ ^24^ ^)^. In addition, the nutritional impact of switching consumption from conventional to organic meat (or that from other high-forage systems) relating to higher *n*-3 PUFA intakes (and conversion of ALA to VLC *n*-3 PUFA) will depend on a range of other dietary factors including total fat intake, the proportion of dairy products, meat and vegetable fat in total fat intake, the type of vegetable fats in the diet and the relative capacity of individuals to convert/elongate ALA into longer-chain *n*-3 PUFA^(^ ^17^ ^,^ ^24^ ^,^ ^63^ ^–^ ^75^ ^)^.

A recent dietary intervention study showed that concentrations of VLC *n*-3 PUFA in both plasma and platelets were significantly higher in individuals consuming pasture-finished compared with concentrate-finished beef and lamb^(^ ^76^ ^)^. This indicates that consumption of meat from grazing/forage-based systems (such as organic meat) may raise VLC *n*-3 concentrations in the human body, although it is currently unclear to what extent this is due to (1) higher VLC *n*-3 intakes or (2) higher ALA to VLC *n*-3 conversion associated with the low LA:ALA ratio in meat from grazing-based systems.

Overall, results of the meta-analyses indicate that the relative impact of using organic production methods on meat FA profiles differs between livestock species. The impact of switching to organic meat consumption therefore not only depends on the amount but also on the type of meat consumed. However, there are large differences in the relative amounts of beef, lamb, pork and chicken meat fat consumed between countries/regions in the EU and elsewhere^(^ ^42^ ^)^. In addition, calculations of estimated FA intakes assumed that (1) fat concentrations in organic and conventional meats are similar and (2) there is no difference in the relative proportion of different types of meat consumed by organic and conventional consumers, whereas there is insufficient published information to confirm that these assumptions are correct. However, it is well documented that (1) meat intakes vary considerably between individuals, (2) the FA composition of intramuscular fat may differ significantly from that of subcutaneous/storage^(^ ^48^ ^)^ and (3) meat processing and consumption methods (e.g. amount of fat being removed) may greatly affect both total fat and FA intakes. Estimates of total daily FA intakes calculated using data on current average EU meat fat consumption therefore have to be interpreted with caution.

The currently very high level of meat, particularly red meat, consumption is thought to be nutritionally undesirable, as it has been linked to obesity, CVD, type 2 diabetes and a range of cancers^(^ ^77^ ^)^. Current dietary recommendations in the USA and Europe are to reduce red meat intakes to <70 g/d^(^ ^78^ ^,^ ^79^ ^)^. Compliance with these guidelines will substantially reduce total fat and VLC *n*-3 intakes. The need to identify alternative approaches to increase VLC *n*-3 PUFA intake is discussed in the supplementary data (see online additional discussion section).

#### Minerals

Owing to the very limited evidence base, it is not currently possible to estimate differences in mineral composition and potential impacts on human health. The need to investigate the potential effects of organic and conventional production protocols on the mineral composition of meat is discussed in the supplementary data (see online additional discussion section).

### Deficiencies in the evidence base

#### Meat composition data

Compared with the large amount of comparative composition data now available for crop-based foods^(^ ^25^ ^)^, the data sets available for the meta-analyses of meat composition parameters reported in this study were limited. Results showed low statistical power for many parameters and limited ability to understand between-study heterogeneity, and these are the major reasons for the very low or low overall reliability for many of the outcomes. However, for a range of composition parameters for which significant differences were detected, the method of synthesis did not have large effects, in terms of either statistical significance or effect magnitude. Additional data from further, well-designed studies would alleviate the current uncertainties in the evidence and may allow exploration of between-study covariates. Future studies should be registered to eliminate potential publication biases. Apart from FA profiles, a particular emphasis should be placed on comparing nutritionally important meat composition parameters for which there are currently no or too few studies to carry out meta-analyses, especially antioxidants/vitamins (e.g. vitamin A, vitamin B_1_, B_6_ and B_12_, riboflavin, folate, niacin, pantothenic acid) and minerals (e.g. Fe, Zn, Se) for which meat is a major dietary source.

#### Effect of specific agronomic practices

Current knowledge on the effect of feeding regimens on meat quality and the results of the meta-analyses reported in this study suggest that increasing the requirements for grazing and applying further restrictions on the use of concentrate feeds (especially during the finishing period) under organic and other extensive (e.g. pasture-reared) production standards will further improve the nutritional quality of meat and the differential in quality compared with meat products from intensive indoor meat production systems^(^ ^48^ ^)^. However, additional well-designed comparative studies are needed to increase the sensitivity of meta-analyses and to quantify more specifically which production system parameters (e.g. specific feed composition components, especially during the finishing period, breed choice/breeding systems, veterinary interventions) are the most significant drivers for nutritionally relevant composition differences for different livestock species.

#### Dietary intervention and cohort studies

Potential impacts of composition differences in meat composition on human health (e.g. risk of CVD) currently have to be extrapolated from existing information about the effects of compounds such as 12 : 0, 14 : 0 and 16 : 0 SFA, LA and *n*-3 (especially VLC *n*-3) PUFA on human health, as there are a few studies that have assessed impacts of organic food consumption on animal or human health or health-related biomarkers. If the significant differences in nutritionally relevant compounds identified in this study are confirmed, this would highlight the need to carry out human dietary intervention and cohort studies designed to quantify the potential health impacts of switching to organic food production. Experimental studies comparing meat from non-organic forage and concentrate-based production systems suggest that other grazing-based livestock production systems deliver similar improvements in FA profiles^(^ ^43^ ^–^ ^45^ ^)^ and potentially other meat-quality parameters. This should be considered in the design of future dietary intervention/cohort studies.

The potential of carrying out dietary intervention/cohort studies was demonstrated by a recent investigation into the effect of organic milk consumption on eczema in children younger than 2 years of age in the Netherlands (a country with relatively high milk consumption)^(^ ^64^ ^)^. It reported that eczema was significantly reduced in children from families consuming organic rather than non-organic milk. This may have been caused by the higher *n*-3 PUFA concentrations and lower *n*-6:*n*-3 PUFA ratio in organic milk, as there is increasing evidence for anti-allergic effects of *n*-3 FA^(^ ^65^ ^)^ – for example, a recent animal study showed that increasing dietary VLC *n*-3 PUFA intake prevents allergic sensitisation to cows’ milk protein in mice^(^ ^66^ ^)^. However, it is important to point out that there are so far no cohort studies showing a link between organic meat consumption and reduced incidence in eczema and other positive health outcomes.

Overall, the present study indicates that organic livestock production may change the FA profiles, and possibly other composition parameters, and that some of these changes (e.g. higher *n*-3 PUFA) may be nutritionally desirable. It is therefore important to carry out additional studies to address the limitations in the current evidence base. If nutritionally relevant composition differences can be confirmed and/or linked to specific agronomic practices (e.g. high forage diets), this would then justify dietary intervention or cohort studies designed to identify the impact of consuming meat with contrasting composition generated by switching to organic production or specific agronomic practices.

## Supplementary material

For supplementary material accompanying this paper visit http://dx.doi.org/10.1017/S0007114515005073. click here to view supplementary material
