# Supplementary material for: Composition differences between organic and conventional meat: a systematic literature review and meta-analysis
Source: Br J Nutr. 2016 Mar 28;115(6):994–1011. doi: 10.1017/S0007114515005073 (PMC4838835; doi:10.1017/S0007114515005073)
Supplement: Supplementary file 1 [file S0007114515005073sup.zip › S0007114515005073sup001.docx]

Composition differences between organic and conventional meat;
a systematic literature review and meta-analysis

APPENDIX

Table A1 below provides results of the standard and sensitivity 1 meta-analyses, and sensitivity analysis 2 and 3 to allow the effect of using different data handling and inclusion criteria, and meta-analysis methods (weighted vs unweighted) on the results of meta-analyses to be assessed (see online supplementary Table S5 for summary of inclusion criteria).

Table A2 below provides results of the weighted sensitivity meta-analysis (sensitivity analysis 4) to allow the effect of exclusion of 20% of studies with the least precise treatment effects on the results of meta-analyses to be assessed (see online supplementary Table S5 for summary of inclusion criteria).

Table A3 below provides results of weighted sensitivity meta-analysis (sensitivity analysis 5) after exclusion of studies considered to have less scientifically sound methods of fatty acids assessments (see online supplementary Table S5 for inclusion criteria).

| **Table A1.** Results of meta-analysis comparing composition of organic (ORG) vs conventional (CONV) meat using two different protocols. | | | | | | | | | | | | | | |
| --- | --- | --- | --- | --- | --- | --- | --- | --- | --- | --- | --- | --- | --- | --- |
|  |  | **Weighted meta-analyses** | | | | | | |  | **Unweighted meta-analyses** | | | | |
| **Parameter** | **an*** | ***n*** | **SMD** | **95% CI** | ***P***† | **Heterog.**‡ | **MPD**§ | **95% CI** |  | ***n*** | **Ln ratio**\|\| | ***P***† | **MPD**§ | **95% CI** |
| *Major components* | |  |  |  |  |  |  |  |  |  |  |  |  |  |
| Fat | S,1 | 22 | -0.35 | -0.80, 0.10 | 0.125 | Yes (89%) | -22.21 | -43.92, -0.51 |  | 34 | 4.45 | 0.012 | -21.46 | -40.34, -2.59 |
|  | 2,3 | 22 | -0.35 | -0.80, 0.10 | 0.125 | Yes (89%) | -22.21 | -43.92, -0.51 |  | 38 | 4.46 | 0.028 | -18.93 | -38.90, 1.04 |
| Intramuscular fat | S,1 | 7 | -0.25 | -0.74, 0.25 | 0.331 | Yes (79%) | -12.40 | -37.76, 12.97 |  | 9 | 4.44 | 0.065 | -21.73 | -45.58, 2.12 |
|  | 2,3 | 8 | -0.16 | -0.62, 0.31 | 0.509 | Yes (79%) | -8.82 | -31.87, 14.24 |  | 12 | 4.44 | 0.029 | -20.84 | -39.97, -1.71 |
| Protein | S,1 | 17 | 0.19 | -0.17, 0.54 | 0.307 | Yes (78%) | 1.02 | -0.51, 2.55 |  | 23 | 4.62 | 0.059 | 1.07 | -0.20, 2.34 |
|  | 2,3 | 17 | 0.19 | -0.17, 0.54 | 0.307 | Yes (78%) | 1.02 | -0.51, 2.55 |  | 25 | 4.62 | 0.044 | 1.08 | -0.13, 2.29 |
| *Fatty acids* |  |  |  |  |  |  |  |  |  |  |  |  |  |  |
| ALA (cis-9,12,15-18:3) | S,1 | 22 | 0.70 | -0.30, 1.71 | 0.169 | Yes (97%) | 17.00 | -11.49, 45.49 |  | 32 | 4.80 | 0.008 | 35.08 | 1.34, 68.82 |
|  | 2,3 | 24 | 0.90 | -0.16, 1.97 | 0.097 | Yes (97%) | 32.01 | -16.06, 80.08 |  | 35 | 4.87 | 0.005 | 56.06 | 9.40, 102.72 |
| SFA | S,1 | 26 | -0.35 | -0.79, 0.10 | 0.127 | Yes (92%) | -2.37 | -5.69, 0.94 |  | 38 | 4.59 | 0.103 | -1.67 | -4.17, 0.83 |
|  | 2,3 | 27 | -0.36 | -0.79, 0.07 | 0.101 | Yes (91%) | -3.57 | -7.54, 0.39 |  | 40 | 4.59 | 0.084 | -2.22 | -5.16, 0.72 |
| 12:0  (lauric acid) | S,1 | 11 | -0.01 | -0.55, 0.53 | 0.974 | Yes (84%) | 4.94 | -21.44, 31.31 |  | 15 | 4.66 | 0.270 | 7.94 | -12.61, 28.48 |
|  | 2,3 | 11 | -0.01 | -0.55, 0.53 | 0.974 | Yes (84%) | 4.94 | -21.44, 31.31 |  | 16 | 4.66 | 0.228 | 8.33 | -10.90, 27.57 |
| 14:0  (myristic acid) | S,1 | 23 | -1.06 | -2.12, -0.01 | 0.049 | Yes (98%) | -18.35 | -31.97, -4.72 |  | 27 | 4.47 | 0.003 | -18.11 | -30.25, -5.97 |
|  | 2,3 | 25 | -1.23 | -2.27, -0.20 | 0.020 | Yes (98%) | -20.87 | -34.16, -7.58 |  | 30 | 4.44 | <0.001 | -20.83 | -32.41, -9.25 |
| n, number of data points included in the comparison; MPD, mean percent difference; SMD, standardised mean difference of random-effect model; CI, 95% confidence intervals; SFA, saturated fatty acids; ALA, α-linolenic acid. *Analysis: S, standard meta-analysis, 1,2,3, sensitivity analysis 1, 2 and 3 (see main article and online supplementary Table S5 for details); †*P* value <0.05 indicates significance of the difference in composition between organic and conventional meat; ‡Heterogeneity and the I^2^ Statistic; §Magnitude of difference between organic and conventional samples (value <0 indicate higher concentration in CONV, value >0 indicate higher concentration in ORG); \|\|Ln ratio = Ln(ORG/CONV × 100%). | | | | | | | | | | | | | | |

| **Table A1 cont.** Results of meta-analysis comparing composition of organic (ORG) vs conventional (CONV) meat using two different protocols. | | | | | | | | | | | | | | |
| --- | --- | --- | --- | --- | --- | --- | --- | --- | --- | --- | --- | --- | --- | --- |
|  |  | **Weighted meta-analyses** | | | | | | |  | **Unweighted meta-analyses** | | | | |
| **Parameter** | **an*** | ***n*** | **SMD** | **95% CI** | ***P***† | **Heterog.**‡ | **MPD**§ | **95% CI** |  | ***n*** | **Ln ratio**\|\| | ***P***† | **MPD**§ | **95% CI** |
| 16:0  (palmitic acid) | S,1 | 24 | -0.51 | -1.01, -0.01 | 0.044 | Yes (91%) | -10.85 | -27.67, 5.98 |  | 30 | 4.55 | 0.043 | -8.50 | -22.03, 5.02 |
|  | 2,3 | 26 | -0.44 | -0.94, 0.05 | 0.080 | Yes (91%) | -14.02 | -31.44, 3.40 |  | 33 | 4.54 | 0.031 | -10.98 | -24.81, 2.86 |
| 20:0  (arachidic acid) | S,1 | 9 | 0.33 | -0.15, 0.81 | 0.177 | Yes (81%) | 53.61 | -39.93, 147.16 |  | 12 | 4.91 | 0.020 | 66.98 | -14.18, 148.14 |
|  | 2,3 | 9 | 0.33 | -0.15, 0.81 | 0.177 | Yes (81%) | 53.61 | -39.93, 147.16 |  | 13 | 4.98 | 0.008 | 79.77 | 1.02, 158.53 |
| MUFA | S,1 | 24 | -1.01 | -1.57, -0.45 | <0.001 | Yes (94%) | -7.97 | -12.47, -3.48 |  | 36 | 4.55 | <0.001 | -6.55 | -10.01, -3.09 |
|  | 2,3 | 24 | -1.01 | -1.57, -0.45 | <0.001 | Yes (94%) | -7.97 | -12.47, -3.48 |  | 37 | 4.54 | <0.001 | -6.77 | -10.16, -3.38 |
| 14:1 | S,1 | 4 | -0.02 | -0.43, 0.39 | 0.909 | No (0%) | -1.85 | -15.46, 11.77 |  | 6 | 4.42 | 0.141 | -27.93 | -76.62, 20.77 |
|  | 2,3 | 5 | -0.24 | -0.74, 0.26 | 0.346 | Yes (33%) | -18.04 | -51.47, 15.4 |  | 8 | 4.25 | 0.035 | -57.86 | -115.49, -0.22 |
| 16:1  (palmitoleic acid) | S,1 | 18 | -0.10 | -0.36, 0.16 | 0.443 | Yes (53%) | -9.10 | -30.33, 12.13 |  | 23 | 4.55 | 0.182 | -10.04 | -27.65, 7.57 |
|  | 2,3 | 20 | -0.14 | -0.39, 0.11 | 0.272 | Yes (49%) | -15.81 | -38.15, 6.53 |  | 26 | 4.49 | 0.041 | -18.19 | -36.95, 0.57 |
| OA (cis-9-18:1) | S,1 | 22 | -0.48 | -1.12, 0.16 | 0.138 | Yes (94%) | -3.71 | -8.43, 1.01 |  | 27 | 4.56 | 0.016 | -4.91 | -9.16, -0.66 |
|  | 2,3 | 23 | -0.49 | -1.09, 0.12 | 0.113 | Yes (94%) | -4.00 | -8.55, 0.54 |  | 29 | 4.56 | 0.006 | -5.35 | -9.35, -1.35 |
| PUFA | S,1 | 23 | 1.15 | 0.51, 1.80 | <0.001 | Yes (95%) | 23.29 | 11.27, 35.31 |  | 35 | 4.75 | <0.001 | 18.90 | 7.28, 30.51 |
|  | 2,3 | 23 | 1.15 | 0.51, 1.80 | <0.001 | Yes (95%) | 23.29 | 11.27, 35.31 |  | 36 | 4.77 | <0.001 | 21.05 | 9.00, 33.1 |
| n-3 FA | S,1 | 21 | 1.31 | 0.16, 2.45 | 0.026 | Yes (98%) | 46.99 | 10.08, 83.89 |  | 31 | 4.85 | <0.001 | 38.38 | 12.16, 64.61 |
|  | 2,3 | 22 | 1.30 | 0.21, 2.38 | 0.019 | Yes (98%) | 52.27 | 15.59, 88.94 |  | 32 | 4.87 | <0.001 | 42.28 | 15.77, 68.8 |
| n, number of data points included in the comparison; MPD, mean percent difference; SMD, standardised mean difference of random-effect model; CI, 95% confidence intervals; MUFA, monounsaturated fatty acids; OA, oleic acid; PUFA, polyunsaturated fatty acids; FA, fatty acids. *Analysis: S, standard meta-analysis, 1,2,3, sensitivity analysis 1, 2 and 3 (see main article and online supplementary Table S5 for details); †*P* value <0.05 indicates significance of the difference in composition between organic and conventional meat; ‡Heterogeneity and the I^2^ Statistic; §Magnitude of difference between organic and conventional samples (value <0 indicate higher concentration in CONV, value >0 indicate higher concentration in ORG); \|\|Ln ratio = Ln(ORG/CONV × 100%). | | | | | | | | | | | | | | |

| **Table A1 cont.** Results of meta-analysis comparing composition of organic (ORG) vs conventional (CONV) meat using two different protocols. | | | | | | | | | | | | | | | |
| --- | --- | --- | --- | --- | --- | --- | --- | --- | --- | --- | --- | --- | --- | --- | --- |
|  | |  | **Weighted meta-analyses** | | | | | | |  | **Unweighted meta-analyses** | | | | |
| **Parameter** | | **an*** | ***n*** | **SMD** | **95% CI** | ***P***† | **Heterog.**‡ | **MPD**§ | **95% CI** |  | ***n*** | **Ln ratio**\|\| | ***P***† | **MPD**§ | **95% CI** |
| EPA (cis-5,8,11,14,17-20:5) | S,1¶ | | 13 | 0.02 | -0.85, 0.89 | 0.966 | Yes (95%) | 0.93 | -37.51, 39.37 |  | 20 | 4.58 | 0.403 | -6.11 | -35.79, 23.56 |
|  | 2,3 | | 16 | -0.24 | -1.03, 0.55 | 0.547 | Yes (94%) | -52.82 | -122.6, 16.9 |  | 25 | 4.55 | 0.369 | -9.18 | -74.52, 56.17 |
| DPA (cis-7,10,13,16,19-22:5) | | S,1 | 11 | 0.40 | -0.36, 1.17 | 0.304 | Yes (92%) | 30.45 | -0.18, 61.07 |  | 15 | 4.82 | 0.007 | 29.49 | 7.07, 51.91 |
|  |  | 2,3 | 11 | 0.40 | -0.36, 1.17 | 0.304 | Yes (92%) | 30.45 | -0.18, 61.07 |  | 15 | 4.82 | 0.010 | 29.49 | 7.07, 51.91 |
| DHA (cis-4,7,10,13,16,19-22:6) | | S,1 | 14 | 0.17 | -0.24, 0.59 | 0.404 | Yes (75%) | 13.84 | -35.39, 63.07 |  | 22 | 4.68 | 0.246 | 8.63 | -23.91, 41.18 |
|  |  | 2,3 | 15 | 0.10 | -0.33, 0.53 | 0.651 | Yes (77%) | -13.19 | -83.24, 56.86 |  | 23 | 4.61 | 0.492 | -8.77 | -54.93, 37.39 |
| VLC n-3 FA (EPA+DPA +DHA) | | S,1 | - | - | - | - | - | - | - |  | 15 | 4.79 | 0.017 | 24.20 | 3.57, 44.83 |
|  |  | 2,3 | - | - | - | - | - | - | - |  | 15 | 4.79 | 0.020 | 24.20 | 3.57, 44.83 |
| n-6 FA | | S,1 | 19 | 0.97 | 0.15, 1.78 | 0.020 | Yes (96%) | 16.34 | 1.73, 30.94 |  | 29 | 4.70 | 0.010 | 12.57 | 1.92, 23.22 |
|  |  | 2,3 | 19 | 0.97 | 0.15, 1.78 | 0.020 | Yes (96%) | 16.34 | 1.73, 30.94 |  | 29 | 4.70 | 0.008 | 12.57 | 1.92, 23.22 |
| LA (cis-9,12-18:2) | | S,1 | 23 | 0.61 | -0.07, 1.29 | 0.077 | Yes (95%) | 8.53 | -11.48, 28.55 |  | 30 | 4.68 | 0.097 | 9.69 | -7.07, 26.44 |
|  |  | 2,3 | 25 | 0.47 | -0.19, 1.13 | 0.166 | Yes (95%) | -2.51 | -26.27, 21.24 |  | 33 | 4.64 | 0.286 | 3.36 | -16.29, 23.01 |
| AA (cis-5,8,11,14-20:4) | S,1¶ | | 13 | 0.45 | -0.05, 0.94 | 0.079 | Yes (80%) | 11.67 | -8.16, 31.50 |  | 19 | 4.61 | 0.461 | 1.40 | -14.68, 17.47 |
|  | 2,3 | | 16 | 0.19 | -0.32, 0.69 | 0.471 | Yes (84%) | -6.96 | -32.28, 18.36 |  | 23 | 4.56 | 0.286 | -6.08 | -26.44, 14.29 |
| n-6/n-3 ratio | | S,1 | 17 | -0.75 | -1.72, 0.23 | 0.133 | Yes (97%) | -21.98 | -46.56, 2.60 |  | 32 | 4.42 | 0.004 | -27.71 | -48.05, -7.38 |
|  |  | 2,3 | 18 | -0.95 | -1.96, 0.06 | 0.066 | Yes (97%) | -66.39 | -156.5, 23.7 |  | 34 | 4.33 | 0.001 | -56.99 | -107.6, -6.4 |
| n, number of data points included in the comparison; MPD, mean percent difference; SMD, standardised mean difference of random-effect model; CI, 95% confidence intervals; EPA, eicosapentaenoic acid; DPA, docosapentaenoic acid; DHA, docosahexaenoic acid; FA, fatty acids; LA, linoleic acid; AA, arachidonic acid. *Analysis: S, standard meta-analysis, 1,2,3, sensitivity analysis 1, 2 and 3 (see main article and online supplementary Table S5 for details); †*P* value <0.05 indicates significance of the difference in composition between organic and conventional meat; ‡Heterogeneity and the I^2^ Statistic; §Magnitude of difference between organic and conventional samples (value <0 indicate higher concentration in CONV, value >0 indicate higher concentration in ORG); \|\|Ln ratio = Ln(ORG/CONV × 100%); ¶Outlying data-pairs for which the % difference between ORG and CONV was over 50 times higher than the mean value were removed. | | | | | | | | | | | | | | | |

| **Table A1 cont.** Results of meta-analysis comparing composition of organic (ORG) vs conventional (CONV) meat using two different protocols. | | | | | | | | | | | | | | |
| --- | --- | --- | --- | --- | --- | --- | --- | --- | --- | --- | --- | --- | --- | --- |
|  |  | **Weighted meta-analyses** | | | | | | |  | **Unweighted meta-analyses** | | | | |
| **Parameter** | **an*** | ***n*** | **SMD** | **95% CI** | ***P***† | **Heterog.**‡ | **MPD**§ | **95% CI** |  | ***n*** | **Ln ratio**\|\| | ***P***† | **MPD**§ | **95% CI** |
| PUFA/SFA ratio | S,1 | 4 | 2.75 | -2.05, 7.55 | 0.261 | Yes (100%) | 50.44 | -33.29, 134.16 |  | 10 | 4.85 | 0.015 | 36.28 | -1.26, 73.82 |
|  | 2,3 | 4 | 2.75 | -2.05, 7.55 | 0.261 | Yes (100%) | 50.44 | -33.29, 134.16 |  | 11 | 4.88 | 0.006 | 41.06 | 5.84, 76.29 |
| *Minerals and undesirable metals* | | | |  |  |  |  |  |  |  |  |  |  |  |
| Copper (Cu) | S,1 | 3 | -4.77 | -8.92, -0.63 | 0.024 | Yes (98%) | -25.96 | -42.61, -9.30 |  | 4 | 4.36 | 0.064 | -27.80 | -40.12, -15.48 |
|  | 2,3 | 5 | -5.24 | -7.58, -2.9 | <0.001 | Yes (96%) | -30.13 | -41.66, -18.6 |  | 6 | 4.34 | 0.015 | -30.67 | -40.14, -21.19 |
| Iron (Fe) | S,1 | 4 | 1.00 | -0.65, 2.66 | 0.236 | Yes (96%) | 13.79 | 2.14, 25.43 |  | 5 | 4.77 | 0.068 | 18.86 | 5.44, 32.27 |
|  | 2,3 | 6 | 1.24 | -0.15, 2.64 | 0.081 | Yes (97%) | 13.11 | 3.60, 22.61 |  | 7 | 4.75 | 0.028 | 16.83 | 5.98, 27.67 |
| Selenium (Se) | S,1 | - | - | - | - | - | - | - |  | 3 | 4.53 | 0.256 | -8.12 | -27.34, 11.10 |
|  | 2,3 | 4 | -2.56 | -3.11, -2.01 | <0.001 | Yes (74%) | -17.99 | -25.73, -10.25 |  | 5 | 4.50 | 0.097 | -12.17 | -25.06, 0.72 |
| *Other* |  |  |  |  |  |  |  |  |  |  |  |  |  |  |
| Cholesterol | S,1 | - | - | - | - | - | - | - |  | 5 | 4.58 | 0.189 | -3.01 | -10.60, 4.59 |
|  | 2,3 | - | - | - | - | - | - | - |  | 6 | 4.57 | 0.145 | -3.48 | -9.75, 2.79 |
| Atherogenicity index¶ | S,1 | 4 | 0.47 | -0.17, 1.11 | 0.148 | Yes (79%) | 6.64 | -0.66, 13.94 |  | 5 | 4.67 | 0.062 | 7.02 | 1.32, 12.72 |
|  | 2,3 | 4 | 0.47 | -0.17, 1.11 | 0.148 | Yes (79%) | 6.64 | -0.66, 13.94 |  | 5 | 4.67 | 0.058 | 7.02 | 1.32, 12.72 |
| Thrombogenicity index¶ | S,1 | 4 | -0.35 | -0.64, -0.06 | 0.018 | No (0%) | -4.40 | -6.73, -2.08 |  | 5 | 4.57 | 0.028 | -3.99 | -5.97, -2.02 |
|  | 2,3 | 4 | -0.35 | -0.64, -0.06 | 0.018 | No (0%) | -4.40 | -6.73, -2.08 |  | 5 | 4.57 | 0.029 | -3.99 | -5.97, -2.02 |
| n, number of data points included in the comparison; MPD, mean percent difference; SMD, standardised mean difference of random-effect model; CI, 95% confidence intervals; PUFA, polyunsaturated fatty acids; SFA, saturated fatty acids. *Analysis: S, standard meta-analysis, 1,2,3, sensitivity analysis 1, 2 and 3 (see main article and online supplementary Table S5 for details); †*P* value <0.05 indicates significance of the difference in composition between organic and conventional meat; ‡Heterogeneity and the I^2^ Statistic; §Magnitude of difference between organic and conventional samples (value <0 indicate higher concentration in CONV, value >0 indicate higher concentration in ORG); \|\|Ln ratio = Ln(ORG/CONV × 100%); ¶Reported in publications. | | | | | | | | | | | | | | |

| **Table A2.** Weighted meta-analysis results after exclusion of 20% of studies with the least precise treatment effects (sensitivity anlaysis 4, see main article and online supplementary Table S5 for details) for parameters shown in Fig. 3 and 4 of the main paper. | | | | | | | |
| --- | --- | --- | --- | --- | --- | --- | --- |
| **Parameter** | ***n*** | **SMD** | **95% CI** | ***P**** | **Heterogeneity**† | **MPD**‡ | **95% CI** |
| *Primary outcome* |  |  |  |  |  |  |  |
| PUFA | 18 | 0.89 | 0.46, 1.33 | <0.001 | Yes (89%) | 18.93 | 10.03, 27.84 |
| n-3 FA | 17 | 0.56 | -0.12, 1.23 | 0.104 | Yes (94%) | 35.43 | -2.24, 73.11 |
| *Scondary outcome* |  |  |  |  |  |  |  |
| 12:0 (lauric acid) | 9 | -0.07 | -0.68, 0.55 | 0.835 | Yes (87%) | 0.17 | -25.6, 25.93 |
| 14:0 (myristic acid) | 18 | -0.09 | -0.41, 0.24 | 0.593 | Yes (76%) | -4.94 | -13.34, 3.47 |
| 16:0 (palmitic acid) | 19 | -0.25 | -0.71, 0.2 | 0.275 | Yes (89%) | -1.07 | -3.86, 1.72 |
| *Exploratory outcome* |  |  |  |  |  |  |  |
| Fat | 18 | -0.53 | -0.99, -0.08 | 0.021 | Yes (89%) | -31.78 | -55.21, -8.35 |
| Intramuscular fat | 6 | -0.06 | -0.46, 0.33 | 0.765 | Yes (64%) | -1.37 | -17.06, 14.33 |
| SFA | 21 | -0.27 | -0.58, 0.04 | 0.092 | Yes (82%) | -1.83 | -5.43, 1.77 |
| MUFA | 19 | -0.83 | -1.25, -0.42 | <0.001 | Yes (89%) | -7.87 | -12.5, -3.24 |
| OA (cis-9-18:1) | 18 | -0.24 | -0.61, 0.12 | 0.194 | Yes (81%) | -0.63 | -3.73, 2.47 |
| ALA (cis-9,12,15-18:3) | 18 | 0.17 | -0.28, 0.63 | 0.460 | Yes (84%) | 10.57 | -3.82, 24.96 |
| EPA (cis-5,8,11,14,17-20:5)§ | 10 | -0.38 | -1.14, 0.37 | 0.321 | Yes (93%) | -5.34 | -49.3, 38.63 |
| DPA (cis-7,10,13,16,19-22:5) | 9 | 0.17 | -0.47, 0.8 | 0.607 | Yes (89%) | 25.74 | -6.41, 57.89 |
| DHA (cis-4,7,10,13,16,19-22:6) | 11 | 0.19 | -0.27, 0.65 | 0.408 | Yes (80%) | 29.08 | -17.8, 75.97 |
| n-6 FA | 15 | 0.56 | 0.08, 1.04 | 0.022 | Yes (88%) | 8.34 | -2.27, 18.96 |
| LA (cis-9,12-18:2) | 18 | 0.43 | 0.09, 0.78 | 0.014 | Yes (78%) | 13.24 | 2.49, 23.99 |
| AA (cis-5,8,11,14-20:4)§ | 10 | 0.19 | -0.28, 0.66 | 0.427 | Yes (76%) | -0.65 | -19.25, 17.95 |
| n-6/n-3 ratio | 14 | -0.18 | -0.96, 0.6 | 0.651 | Yes (95%) | -9.87 | -31.28, 11.55 |
| *n*, number of data points included in the comparison; SMD, standardised mean difference of random-effect model; CI, 95% confidence intervals; MPD, mean percentage difference; PUFA, polyunsaturated fatty acids; FA, fatty acids; SFA, saturated fatty acids; MUFA, monounsaturated fatty acids; OA, oleic acid; ALA, α-linolenic acid; EPA, eicosapentaenoic acid; DPA, docosapentaenoic acid; DHA, docosahexaenoic acid; LA, linoleic acid; AA, arachidonic acid. **P* value <0.05 indicates significance of the difference in composition between organic and conventional meat; †Heterogeneity and the I^2^ Statistic; ‡Magnitude of difference between organic (ORG) and conventional (CONV) samples (value <0 indicate higher concentration in CONV, value >0 indicate higher concentration in ORG); §Outlying data pairs (where the MPD between ORG and CONV was over fifty times greater than the mean value including the outliers) were removed. | | | | | | | |

| **Table A3.** Weighted meta-analysis results after exclusion of studies considered to have less scientifically sound methods of fatty acids assessments (sensitivity analysis 5, see main article and online supplementary Table S5 for details) for parameters shown in Fig. 3 and 4 of the main paper. | | | | | | | |
| --- | --- | --- | --- | --- | --- | --- | --- |
| **Parameter** | ***n*** | **SMD** | **95% CI** | ***P**** | **Heterogeneity**† | **MPD**‡ | **95% CI** |
| *Primary outcome* |  |  |  |  |  |  |  |
| PUFA | 20 | 1.31 | 0.56, 2.07 | 0.001 | Yes (96%) | 25.49 | 11.96, 39.01 |
| n-3 FA | 18 | 1.36 | -0.01, 2.74 | 0.052 | Yes (98%) | 43.69 | 1.85, 85.53 |
| *Scondary outcome* |  |  |  |  |  |  |  |
| 12:0 (lauric acid) | 9 | -0.03 | -0.71, 0.64 | 0.927 | Yes (86%) | 0.58 | -30.54, 31.71 |
| 14:0 (myristic acid) | 21 | -1.21 | -2.40, -0.02 | 0.047 | Yes (98%) | -20.82 | -35.27, -6.36 |
| 16:0 (palmitic acid) | 22 | -0.57 | -1.13, -0.01 | 0.046 | Yes (92%) | -11.53 | -29.89, 6.83 |
| *Exploratory outcome* |  |  |  |  |  |  |  |
| Fat | 18 | -0.36 | -0.90, 0.17 | 0.183 | Yes (90%) | -13.70 | -33.32, 5.92 |
| Intramuscular fat | 7 | -0.25 | -0.74, 0.25 | 0.331 | Yes (79%) | -12.40 | -37.76, 12.97 |
| SFA | 23 | -0.45 | -0.94, 0.04 | 0.069 | Yes (92%) | -3.57 | -6.90, -0.24 |
| MUFA | 21 | -1.01 | -1.64, -0.38 | 0.002 | Yes (95%) | -6.82 | -11.20, -2.43 |
| OA (cis-9-18:1) | 20 | -0.53 | -1.25, 0.18 | 0.144 | Yes (95%) | -3.88 | -9.05, 1.28 |
| ALA (cis-9,12,15-18:3) | 21 | 0.74 | -0.33, 1.80 | 0.175 | Yes (97%) | 16.94 | -12.94, 46.82 |
| EPA (cis-5,8,11,14,17-20:5)§ | 13 | -0.21 | -1.10, 0.68 | 0.643 | Yes (94%) | -36.01 | -90.93, 18.92 |
| DPA (cis-7,10,13,16,19-22:5) | 10 | 0.45 | -0.41, 1.31 | 0.307 | Yes (93%) | 32.80 | -0.67, 66.27 |
| DHA (cis-4,7,10,13,16,19-22:6)§ | 8 | -0.04 | -0.49, 0.40 | 0.844 | Yes (62%) | 6.13 | -17.57, 29.83 |
| n-6 FA | 17 | 1.09 | 0.17, 2.01 | 0.020 | Yes (96%) | 16.57 | 0.43, 32.71 |
| LA (cis-9,12-18:2) | 22 | 0.64 | -0.08, 1.37 | 0.082 | Yes (95%) | 7.75 | -13.13, 28.64 |
| AA (cis-5,8,11,14-20:4)§ | 10 | 0.24 | -0.25, 0.73 | 0.335 | Yes (76%) | 0.83 | -18.31, 19.96 |
| n-6/n-3 ratio | 16 | -0.52 | -1.44, 0.40 | 0.265 | Yes (97%) | -13.70 | -33.34, 5.95 |
| *n*, number of data points included in the comparison; SMD, standardised mean difference of random-effect model; CI, 95% confidence intervals; MPD, mean percentage difference; PUFA, polyunsaturated fatty acids; FA, fatty acids; SFA, saturated fatty acids; MUFA, monounsaturated fatty acids; OA, oleic acid; ALA, α-linolenic acid; EPA, eicosapentaenoic acid; DPA, docosapentaenoic acid; DHA, docosahexaenoic acid; LA, linoleic acid; AA, arachidonic acid. **P* value <0.05 indicates significance of the difference in composition between organic and conventional meat; †Heterogeneity and the I^2^ Statistic; ‡Magnitude of difference between organic (ORG) and conventional (CONV) samples (value <0 indicate higher concentration in CONV, value >0 indicate higher concentration in ORG); §Outlying data pairs (where the MPD between ORG and CONV was over fifty times greater than the mean value including the outliers) were removed. | | | | | | | |
