# Supplementary material for: Composition differences between organic and conventional meat: a systematic literature review and meta-analysis
Source: Br J Nutr. 2016 Mar 28;115(6):994–1011. doi: 10.1017/S0007114515005073 (PMC4838835; doi:10.1017/S0007114515005073)
Supplement: Supplementary file 1 [file S0007114515005073sup.zip › S0007114515005073sup002.docx]

Composition differences between organic and conventional meat;
a systematic literature review and meta-analysis

SUPPLEMENTARY DATA

**TABLE OF CONTENTS**

[1. LITERATURE REVIEW 4](#_Toc435182352)

[**Table S1.** List of comparison studies included in the meta-analysis. 4](#_Toc435182353)

[**Figure S1.** Number of papers included in the meta-analysis by year of publication. 8](#_Toc435182354)

[**Figure S2.** Number of papers included in the meta-analysis by location of the experiment (country). 9](#_Toc435182355)

[**Table S2.** Study type, location, species/product, animal group and fatty acids analysis method information of the comparison studies included in the meta-analysis. 10](#_Toc435182356)

[**Table S3.** Production systems information for studies with more than two systems. 14](#_Toc435182357)

[**Table S4.** Information extracted from the papers and included in the database used for meta-analysis. 17](#_Toc435182358)

[**Table S5.** Summary of inclusion criteria used in the standard and the sensitivity analyses carried out. Detailed results of the sensitivity analysis are shown in the Appendix on the Newcastle University website (http://research.ncl.ac.uk/nefg/QOF) 17](#_Toc435182359)

[**Table S6.** List of composition parameters included in the statistical analyses. 19](#_Toc435182360)

[**Table S7.** List of composition parameters excluded from the statistical analyses. 20](#_Toc435182361)

[2. ADDITIONAL METHODS 22](#_Toc435182362)

[**2.1.** Calculations used for weighted meta-analyses 22](#_Toc435182363)

[**2.2.** Calculations used for percentage mean differences (MPDs) 22](#_Toc435182364)

[3. ADDITIONAL RESULTS 23](#_Toc435182365)

[**Table S8.** Basic information/statistics on the publications/data used for meta-analyses of composition parameters included in Fig. 2 in the main paper. 24](#_Toc435182366)

[**Table S9.** Mean percentage differences (MPD) and confidence intervals (CI) calculated using the data included in standard meta-analyses and sensitivity meta-analysis 1 of composition parameters shown in Fig. 2 of the main paper (MPDs are also shown as symbols in Fig. 2). 25](#_Toc435182367)

[**Table S10.** Mean percentage differences (MPD) and confidence intervals (CI) calculated using the data included in standard meta-analyses and sensitivity meta-analysis 1 of composition parameters shown in Fig. 3 and 4 of the main paper (MPDs are also shown as symbols in Fig. 3 and 4). 26](#_Toc435182368)

[**Table S11.** Meta-analysis results for additional composition parameters (protein, 20:0 (arachidic acid), 14:1, 16:1 (palmitoleic acid), 17:1 (heptadecenoic acid), CLA (cis-9-trans-11-18:2), PUFA/SFA ratio, n-3/n-6 ratio, EPA+DHA, atherogenicity index, thrombogenicity index, cholesterol, lipid oxidation (TBARS), Cu, Fe) for which significant differences were detected by the standard and sensitivity meta-analysis 1 protocols. 29](#_Toc435182369)

[**Figure S3.** Results of the standard meta-analyses and sensitivity meta-analysis 1 for different study types for fat composition in meat. 30](#_Toc435182370)

[**Figure S4.** Results of the standard meta-analyses and sensitivity meta-analysis 1 for different study types for fat composition in meat. 31](#_Toc435182371)

[**Figure S5.** Results of the standard meta-analyses and sensitivity meta-analysis 1 for different study types for fat composition in meat. 32](#_Toc435182372)

[**Figure S6.** Forest plot showing the results of the comparison of fat content 33](#_Toc435182373)

[**Figure S7.** Forest plot showing the results of the comparison of intramuscular fat content 34](#_Toc435182374)

[**Figure S8.** Forest plot showing the results of the comparison of protein content 35](#_Toc435182375)

[**Figure S9.** Forest plot showing the results of the comparison of saturated fatty acids (SFA) content 36](#_Toc435182376)

[**Figure S10.** Forest plot showing the results of the comparison of 12:0 (lauric acid) content 37](#_Toc435182377)

[**Figure S11.** Forest plot showing the results of the comparison of 14:0 (myristic acid) content 38](#_Toc435182378)

[**Figure S12.** Forest plot showing the results of the comparison of 16:0 (palmitic acid) content 39](#_Toc435182379)

[**Figure S13.** Forest plot showing the results of the comparison of 20:0 (arachidic acid) content 40](#_Toc435182380)

[**Figure S14.** Forest plot showing the results of the comparison of monounsaturated fatty acids (MUFA) content 41](#_Toc435182381)

[**Figure S15.** Forest plot showing the results of the comparison of 14:1 fatty acid content 42](#_Toc435182382)

[**Figure S16.** Forest plot showing the results of the comparison of 16:1 (palmitoleic acid) content 43](#_Toc435182383)

[**Figure S17.** Forest plot showing the results of the comparison of 17:1 (heptadecenoic acid) content 44](#_Toc435182384)

[**Figure S18.** Forest plot showing the results of the comparison of oleic acid (OA, cis-9-18:1) content 45](#_Toc435182385)

[**Figure S19.** Forest plot showing the results of the comparison of polyunsaturated fatty acids (PUFA) content 46](#_Toc435182386)

[**Figure S20.** Forest plot showing the results of the comparison of n-3 fatty acids content 47](#_Toc435182387)

[**Figure S21.** Forest plot showing the results of the comparison of α-linolenic acid (ALA, cis-9,12,15-18:3) content 48](#_Toc435182388)

[**Figure S22.** Forest plot showing the results of the comparison of eicosapentaenoic acid (EPA, cis-5,8,11,14,17-20:5) content 49](#_Toc435182389)

[**Figure S23.** Forest plot showing the results of the comparison of docosapentaenoic acid (DPA, cis-7,10,13,16,19-22:5) content 50](#_Toc435182390)

[**Figure S24.** Forest plot showing the results of the comparison of docosahexaenoic acid (DHA, cis-4,7,10,13,16,19-22:6) content 51](#_Toc435182391)

[**Figure S25.** Forest plot showing the results of the comparison of n-6 fatty acid content 52](#_Toc435182392)

[**Figure S26.** Forest plot showing the results of the comparison of linoleic acid (LA, cis-9,12-18:2) content 53](#_Toc435182393)

[**Figure S27.** Forest plot showing the results of the comparison of conjugated linoleic acid (CLA, cis-9-trans-11-18:2) content 54](#_Toc435182394)

[**Figure S28.** Forest plot showing the results of the comparison of arachidonic acid (AA, cis-5,8,11,14-20:4) content 55](#_Toc435182395)

[**Figure S29.** Forest plot showing the results of the comparison of n-6/-3 ratio 56](#_Toc435182396)

[**Figure S30.** Forest plot showing the results of the comparison of polyunsaturated to saturated fatty acids (PUFA/SFA) ratio 57](#_Toc435182397)

[**Figure S31.** Forest plot showing the results of the comparison of copper (Cu) content 57](#_Toc435182398)

[**Figure S32.** Forest plot showing the results of the comparison of iron (Fe) content 58](#_Toc435182399)

[**Figure S33.** Forest plot showing the results of the comparison of lipid oxidation (TBARS) 58](#_Toc435182400)

[**Figure S34.** Forest plot showing the results of the comparison of atherogenicity index 59](#_Toc435182401)

[**Figure S35.** Forest plot showing the results of the comparison of thrombogenicity index 59](#_Toc435182402)

[**Table S12.** Results of the standard meta-analysis and sensitivity meta-analysis 1 for parameters where none of the meta-analyses protocols detected significant differences between organic and conventional meat. 60](#_Toc435182403)

[**Table S13.** Results of the statistical test for publication bias reported in Table 1 of the main paper. 64](#_Toc435182404)

[4. ADDITIONAL DISCUSSION 65](#_Toc435182405)

[**4.1.** The need to identify alternative approaches to increase VLC n-3 PUFA intake 65](#_Toc435182406)

[**4.2.** The need to carry out additional studies comparing the mineral composition of meat from organic and conventional production 65](#_Toc435182407)

[5. ADDITIONAL REFERENCES 66](#_Toc435182408)

# 1. LITERATURE REVIEW

Supplementary Tables S1 to S7 and Supplementary Figures S1 to S2 provide detailed information on the comparison studies, types of data extracted, data sources and characteristics.

| **Table S1.** List of comparison studies included in the meta-analysis. | | |
| --- | --- | --- |
| **ID** | **Reference** | **SA*** |
| 105 | Angood KM, Wood JD, Nute GR et al. (2008) A comparison of organic and conventionally-produced lamb purchased from three major UK supermarkets: Price, eating quality and fatty acid composition. Meat Science 78, 176-184. | + |
| 578 | Barbieri G, Macchiavelli L & Rivaldi P (2008) Protein quality and content of nitrite, nitrate and metals in commercial samples of organic and conventional cold meats. In Proceedings of the 2nd Conference of the International Society of Organic Agriculture Research ISOFAR, Modena, Italy, June 18-20, 2008: Cultivating the Future Based on Science. |  |
| 658 | Bjorklund EA, Heins BJ, DiCostanzo A et al. (2014) Fatty acid profiles, meat quality, and sensory attributes of organic versus conventional dairy beef steers. Journal of Dairy Science 97, 1828-1834. | + |
| 485 | Blanco-Penedo I, Lopez-Alonso M, Miranda M et al. (2010) Non-essential and essential trace element concentrations in meat from cattle reared under organic, intensive or conventional production systems. Food Additives & Contaminants, Part A: Chemistry, Analysis, Control 27, 36-42. | + |
| 469 | Blanco-Penedo I, Shore RF, Miranda M et al. (2009) Factors affecting trace element status in calves in NW Spain. Livestock Science 123, 198-208. | + |
| 568 | Brown SN, Nute GR, Baker A et al. (2008) Aspects of meat and eating quality of broiler chickens reared under standard, maize-fed, free-range or organic systems. British Poultry Science 49, 118-124. |  |
| 159 | Castellini C, Mugnai C & Dal Bosco A (2002) Effect of organic production system on broiler carcass and meat quality. Meat Science 60, 219-225. | + |
| 646 | Cozzi G, Preciso SF, Gottardo F et al. (2001) Organic rearing as an alternative to intensive beef production systems. L'Informatore Agrario 57, 101-107. | + |
| 645 | de la Torre CA, Conte Junior CA, da Cruz Silva Canto ACV et al. (2012) Biochemical changes in alternative poultry meat during refrigerated storage. Revista Brasileira de Ciencia Veterinaria 19, 195-200. | + |
| 633 | de-la-Vega F, Guzman JL, Delgado-Pertinez M et al. (2013) Fatty acid composition of muscle and adipose tissues of organic and conventional Blanca Andaluza suckling kids. Spanish Journal of Agricultural Research 11, 770-779. | + |
| 634 | de-la-Vega F, Guzman JL, Delgado-Pertinez M et al. (2013) Fatty acid composition of muscle and internal fat depots of organic and conventional Payoya goat kids. Spanish Journal of Agricultural Research 11, 759-769. | + |
| 529 | dos Santos Pinho AP, Jardim Barcellos JO, Peripolli V et al. (2011) Lipid profile of intramuscular fat in meat cattle cuts of commercial brands. Revista Brasileira de Zootecnia 40, 1134-1142. | + |
| 466 | Esterhuizen J, Groenewald IB, Strydom PE et al. (2008) The performance and meat quality of Bonsmara steers raised in a feedlot, on conventional pastures or on organic pastures. South African Journal of Animal Science 38, 303-314. | + |
| 661 | Feng C, Yang S, Shiu J et al. (2011) Effects of organic ration on the carcass characteristics and meat quality of castrated Taiwan native black goat. Journal of Taiwan Livestock Research 44, 213-224. |  |
| 647 | Fischer K (2002) Does the feeding of an organic diet to pigs result in better pork quality? Forschungs-Report 1, 20-23. |  |
| ID, Paper unique identification number. *Papers included in standard meta-analysis: +. | | |

| **Table S1 cont.** List of comparison studies included in the meta-analysis. | | |
| --- | --- | --- |
| **ID** | **Reference** | **SA*** |
| 657 | Garcia-Torres S, Curbelo P, Osorio C et al. (2011) Effect of organic and conventional systems on lipid composition of Longissimus dorsi of beef cattle. In Proceedings of the XIV Jordanas Sobre Produccion Animal, Zaragoza, Spain, May 17-18, 2011., pp. 592-594. Spain. | + |
| 235 | Ghidini S, Zanardi E, Battaglia A et al. (2005) Comparison of contaminant and residue levels in organic and conventional milk and meat products from northern Italy. Food Additives & Contaminants, Part A: Chemistry, Analysis, Control 22, 9-14. |  |
| 635 | Gibbs RA, Rymer C & Givens DI (2013) Fatty acid composition of cooked chicken meat and chicken meat products as influenced by price range at retail. Food Chemistry 138, 1749-1756. | + |
| 563 | Grela ER & Kowalczuk E (2009) Content of nutrients and fatty acid composition in meat and pork-butcher's meat from organic pig production. Zywnosc. Nauka. Technologia. Jakosc. 4, 34-40. | + |
| 167 | Hansen LL, Claudi-Magnussen C, Jensen SK et al. (2006) Effect of organic pig production systems on performance and meat quality. Meat Science 74, 605-615. | + |
| 636 | Hardy B, Crilly N, Pendleton S et al. (2013) Impact of Rearing Conditions on the Microbiological Quality of Raw Retail Poultry Meat. Journal of Food Science 78, M1232-M1235. | + |
| 564 | Hoegberg A, Pickova J, Andersson K et al. (2003) Fatty acid composition and tocopherol content of muscle in pigs fed organic and conventional feed with different n6/n3 ratios, respectively. Food Chemistry 80, 177-186. | + |
| 199 | Husak RL, Sebranek JG & Bregendahl K (2008) A survey of commercially available broilers marketed as organic, free-range, and conventional broilers for cooked meat yields, meat composition, and relative value. Poultry Science 87, 2367-2376. | + |
| 135 | Jahan K & Paterson A (2007) Lipid composition of retailed organic, free-range and coventional chicken breasts. International Journal of Food Science and Technology 42, 251-262. | + |
| 138 | Jahan K, Paterson A & Spickett CM (2004) Fatty acid composition, antioxidants and lipid oxidation in chicken breasts from different production regimes. International Journal of Food Science and Technology 39, 443-453. |  |
| 659 | Jahan K, Paterson A, Piggott J et al. (2005) Chemometric modeling to relate antioxidants, neutral lipid fatty acids, and flavor components in chicken breasts. Poultry Science 84, 158-166. | + |
| 573 | Kamihiro S (2011) Meat quality and fatty acid composition of retail organic and non-organic beef in UK. MSc thesis, Newcastle University. |  |
| 637 | Karwowska M & Dolatowski ZJ (2013) Comparison of lipid and protein oxidation, total iron content and fatty acid profile of conventional and organic pork. International Journal of Food Science and Technology 48, 2200-2206. | + |
| 641 | Kim DH, Cho SH, Kim JH et al. (2009) Comparison of the quality of the chicken breasts from organically and conventionally reared chickens. Korean Journal for Food Science of Animal Resources 29, 409-414. | + |
| 200 | Kim DH, Seong PN, Cho SH et al. (2009) Fatty acid composition and meat quality traits of organically reared Korean native black pigs. Livestock Science 120, 96-102. | + |
| 648 | Kucukylmaz K, Bozkurt M, Catl AU et al. (2012) Chemical composition, fatty acid profile and colour of broiler meat as affected by organic and conventional rearing systems. South African Journal of Animal Science 42, 360-368. |  |
| 569 | Lawlor JB, Sheehan EM, Delahunty CM et al. (2003) Oxidative stability of cooked chicken breast burgers obtained from organic, free-range and conventionally reared animals. International Journal of Poultry Science 2, 398-403. | + |
| 349 | Linden A, Andersson K & Oskarsson A (2001) Cadmium in Organic and Conventional Pig Production. Archives of Environmental Contamination and Toxicology 40, 425-431. | + |
| 604 | Lozicki A, Dymnicka M, Arkuszewska E et al. (2012) Effect of pasture or maize silage feeding on the nutritional value of beef. Annals of Animal Science 12, 81-93. | + |
| ID, Paper unique identification number. *Papers included in standard meta-analysis: +. | | |

| **Table S1 cont.** List of comparison studies included in the meta-analysis. | | |
| --- | --- | --- |
| **ID** | **Reference** | **SA*** |
| 280 | Millet S, Hesta M, Seynaeve M et al. (2004) Performance, meat and carcass traits of fattening pigs with organic versus conventional housing and nutrition. Livestock Production Science 87, 109-119. | + |
| 561 | Millet S, Raes K, Van den Broeck W et al. (2005) Performance and meat quality of organically versus conventionally fed and housed pigs from weaning till slaughtering. Meat Science 69, 335-341. | + |
| 407 | Miotello S (2009) Meat quality of calves obtained from organic and conventional farming. Italian Journal of Animal Science 8, 213-215. | + |
| 560 | Morbidini L, Sarti DM, Pollidori P et al. (2001) Carcass, meat and fat quality in Italian Merino derived lambs obtained with 'organic' farming systems. In Proceedings of the Meeting of the Sub-Network on Production Systems of the FAO-CIHEAM Inter-Regional Cooperative Research and Development Network on Sheep and Goats (Rubino R. (ed.), Morand-Fehr P. (ed.)) pp. 29-34. | + |
| 649 | Morgante M, Piasentier E, Bonanno A et al. (2007) Effect of the dam's feeding regimen on the meat quality of light suckling lambs. Italian Journal of Animal Science 6, 570-572. |  |
| 642 | Nachman KE, Baron PA, Raber G et al. (2013) Roxarsone, inorganic arsenic, and other arsenic species in chicken: A U.S.-based market basket sample. Environmental Health Perspectives 121, 818-824. |  |
| 567 | Nurnberg K, Zupp W, Grumbach S et al. (2006) Does feeding under organic farming conditions affect the meat and fat quality of finishing lambs? Fleischwirtschaft 86, 103-107. | + |
| 397 | Olivan M, Sierra V, Castro P et al. (2009) Carcass and meat quality from yearling bulls managed under organic or conventional systems. In Proceedings of the 60th Annual Meeting of the European Federation of Animal Science (EAAP), August 24-27, 2009. | + |
| 197 | Olsson IM, Jonsson S & Oskarsson A (2001) Cadmium and zinc in kidney, liver, muscle and mammary tissue from dairy cows in conventional and organic farming. Journal of Environmental Monitoring 3, 531-538. | + |
| 188 | Olsson V, Andersson K, Hansson I et al. (2003) Differences in meat quality between organically and conventionally produced pigs. Meat Science 64, 287-297. | + |
| 644 | Olsson V, Solyakov A, Skog K et al. (2002) Natural variations of precursors in pig meat affect the yield of heterocyclic amines - Effects of RN genotype, feeding regime, and sex. Journal of Agricultural and Food Chemistry 50, 2962-2969. | + |
| 209 | Pla M (2008) A comparison of the carcass traits and meat quality of conventionally and organically produced rabbits. Livestock Science 115, 1-12. | + |
| 355 | Pla M, Hernandez P, Arino B et al. (2007) Prediction of fatty acid content in rabbit meat and discrimination between conventional and organic production systems by NIRS methodology. Food Chemistry 100, 165-170. | + |
| 650 | Polat U, Oruc HH, Hanoglu H et al. (2009) Comparative evaluation of biochemical components of blood serum and toxicological parameters of kivircik lambs fed on conventional and organic fodder. Pakistan Journal of Zoology 41, 109-115. | + |
| 574 | Prache S, Gatellier P, Thomas A et al. (2011) Comparison of meat and carcass quality in organically reared and conventionally reared pasture-fed lambs. Animal 5, 2001-2009. | + |
| 516 | Prevolnik M, Ocepek M, Candek-Potokar M et al. (2011) Growth, Carcass and Meat Quality Traits of Pigs Raised under Organic or Conventional Rearing Systems Using Commercially Available Feed Mixtures. Slovenian Veterinary Research 48, 15-26. | + |
| 651 | Razminowicz RH, Kreuzer M & Scheeder MRL (2006) Quality of retail beef from two grass-based production systems in comparison with conventional beef. Meat Science 73, 351-361. |  |
| 652 | Revilla I, Vivar-Quintana AM, Luruena-Martinez MA et al. (2008) Organic vs conventional suckling lamb production: product quality and consumer acceptance. In Proceedings of the 16th IFOAM Organic World Congress, Modena, Italy, June 16-20, 2008. |  |
| ID, Paper unique identification number. *Papers included in standard meta-analysis: +. | | |

| **Table S1 cont.** List of comparison studies included in the meta-analysis. | | |
| --- | --- | --- |
| **ID** | **Reference** | **SA*** |
| 566 | Revilla I, Vivar-Quintana AM, Luruena-Martinez MA et al. (2009) Volatile compounds analysis of suckling lamb meat of conventional and organic production systems. In Proceedings of the XXXIX Jornadas de Estudio, XIII Jornadas sobre Produccion Animal, Zaragoza, Spain, May 12-13, 2009, pp. 523-525. | + |
| 268 | Ristic M, Freudenreich P, Damme K et al. (2007) Meat quality of broilers: a comparison between conventional and organic production. Fleischwirtschaft 87, 114-116. |  |
| 638 | Rosenquist H, Boysen L, Krogh AL et al. (2013) Campylobacter contamination and the relative risk of illness from organic broiler meat in comparison with conventional broiler meat. International Journal of Food Microbiology 162, 226-230. | + |
| 654 | Sanchez Iglesias MJ, Vaquero Martin M, Rubio Hernando B et al. (2012) Study of the characteristics of conventional cooked hams and organic cooked hams. In Proceedings of the 7th International Symposium on the Mediterranean Pig, Cordoba, Spain, October 14-16, 2010., 101 ed., pp. 483-486 [EJ de Pedro and AB Cabezas, editors]. | + |
| 640 | Schiavone A, Peiretti PG, Angulo FMA et al. (2013) Effect of rearing system and genotype on performance, carcass characteristics and meat quality of slow growing rabbits. Large Animal Review 19, 83-87. | + |
| 660 | Sencic D, Kalic G, Steiner Z et al. (2012) Slaughterhouse quality of chicken from organic and conventional housing system. In Proceedings of the 22nd International Scientific-Expert Conference of Agriculture and Food Industry, Sarajevo, Bosnia and Herzegovina, September 28 - October 1, 2011., pp. 56-58. |  |
| 662 | Sencic, Samac D, Antunovic Z et al. (2009) Quality of chicken meat from organic and conventional fattening. Meso 11, 110-113. |  |
| 562 | Smith GC, Heaton KL, Sofos JN et al. (1997) Residues of antibiotics, hormones and pesticides in conventional, natural and organic beef. Journal of Muscle Foods 8, 157-172. |  |
| 521 | Soysal D, Cibik R, Aydin C et al. (2011) Comparison of conventional and organic management conditions on growth performance, carcass characteristics and haematological parameters in Karacabey Merino and Kivircik breeds. Tropical Animal Health and Production 43, 817-823. | + |
| 570 | Urbanczyk J, Hanczakowska E & Swiatkiewicz M (2005) The effect of organic feeding on carcass and meat quality of fattening pigs. Journal of Animal and Feed Sciences 14, 409-412. |  |
| 183 | Walshe BE, Sheehan EM, Delahunty CM et al. (2006) Composition, sensory and shelf life stability analyses of Longissimus dorsi muscle from steers reared under organic and conventional production systems. Meat Science 73, 319-325. | + |
| 603 | Wilches D, Rovira J, Jaime I et al. (2011) Evaluation of the effect of a maternal rearing system on the odour profile of meat from suckling lamb. Meat science 88, 415-423. | + |
| 655 | Zeola NMBL, da Silva Sobrinho AG & Manzi GM (2011) Qualitative parameters of lamb meat submitted to organic and conventional production models. Brazilian Journal of Veterinary Research and Animal Science 48, 107-115. |  |
| 606 | Zeola NMBL, da Silva Sobrinho AG & Manzi GM (2011) Regional and centesimal composition of carcass of lambs raised under conventional and organic production models. Revista Brasileira de Zootecnia 40, 2963-2970. | + |
| 605 | Zeola NMBL, da Silva Sobrinho AG, Borba H et al. (2012) Evaluation of the production model and fat inclusion in qualitative and sensorial parameters of the sheep hamburgers. Arquivo Brasileiro De Medicina Veterinaria E Zootecnia 64, 727-734. |  |
| ID, Paper unique identification number. *Papers included in standard meta-analysis: +. | | |

**
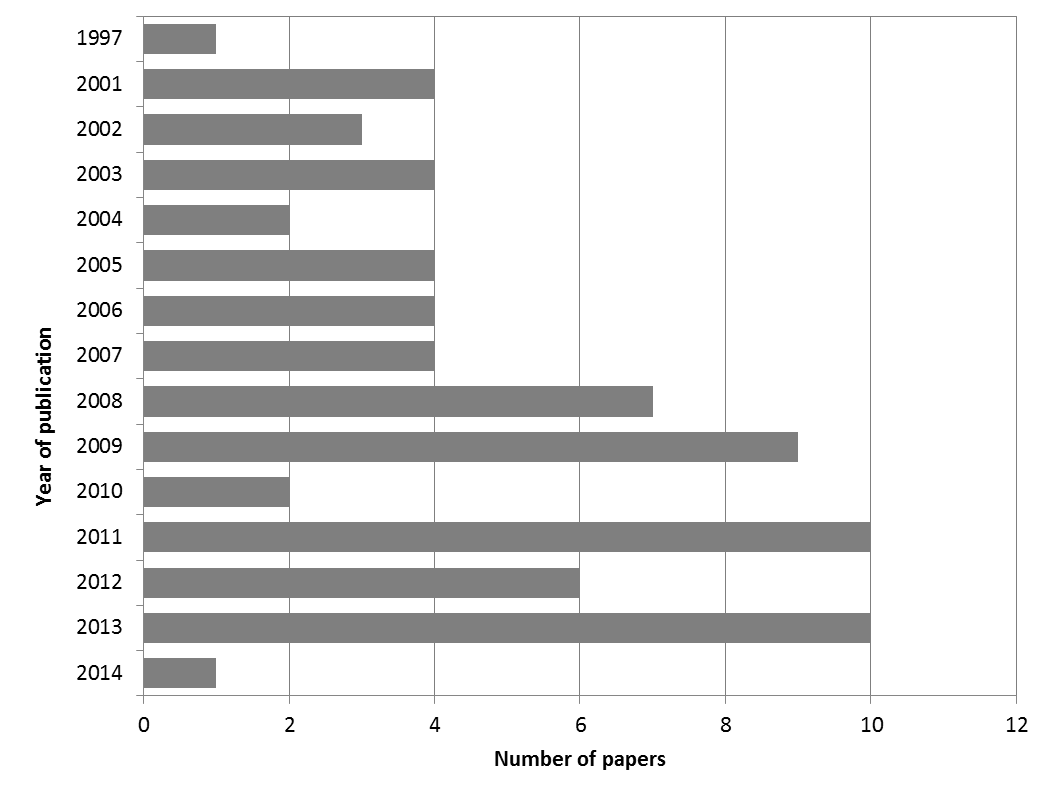
**

## **Figure S1.** Number of papers included in the meta-analysis by year of publication.

**
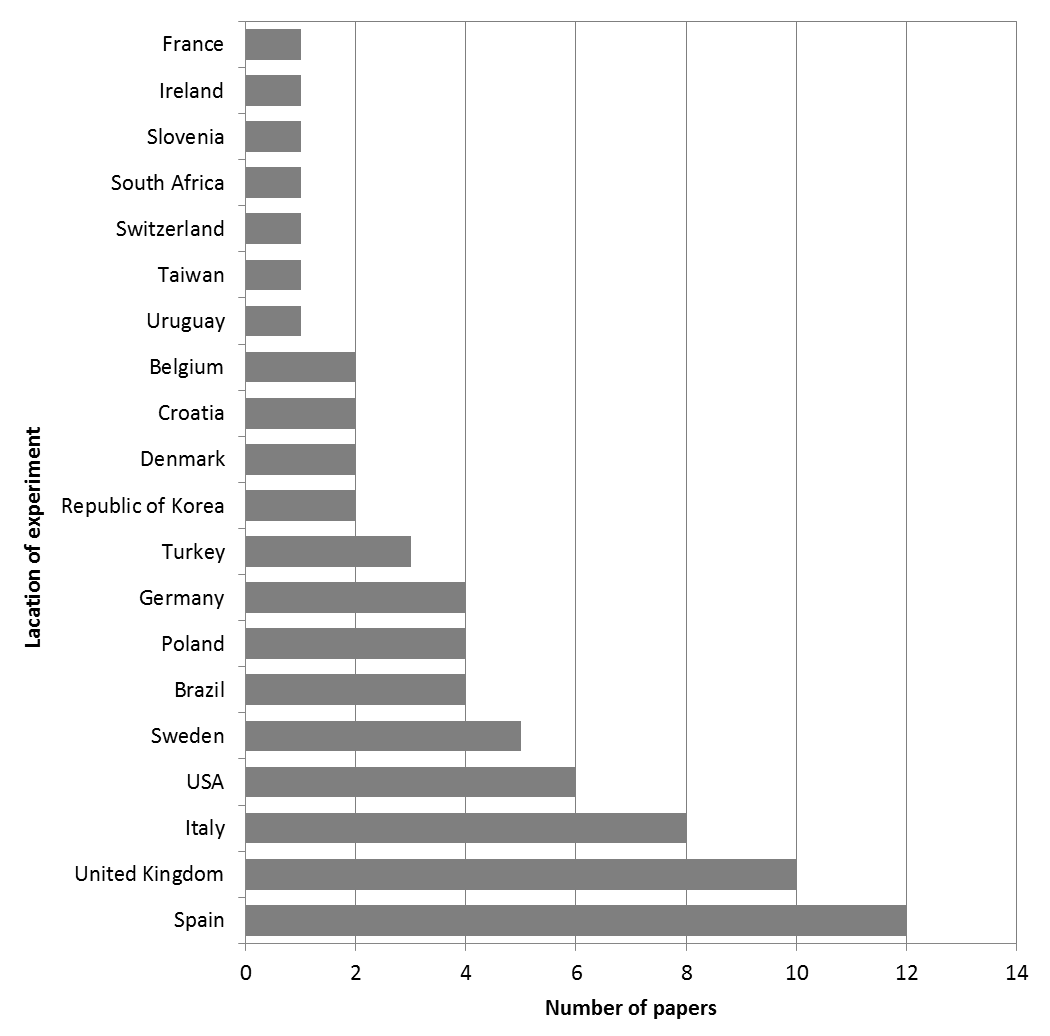
**

## **Figure S2.** Number of papers included in the meta-analysis by location of the experiment (country).

| **Table S2.** Study type, location, species/product, animal group and fatty acids analysis method information of the comparison studies included in the meta-analysis. | | | | | |
| --- | --- | --- | --- | --- | --- |
| **ID** | **ST** | **Location** | **Product*** | **Animal group** | **AM** |
| 105 | BS | United Kingdom | lamb (m. Longissimus lumborum) | Lamb and goat meat | GC |
| 135 | BS | United Kingdom | chicken (breast, m. Pectoralis major) | Chicken meat | GC |
| 138 | BS | United Kingdom | chicken (breast, m. Pectoralis major) | Chicken meat | GC |
| 159 | EX | Italy | chicken (breast, drumstick) | Chicken meat | GC† |
|  |  |  | chicken (carcass) | Chicken meat |  |
| 167 | EX | Denmark | pig (back fat) | Pork | GC |
|  |  |  | pig (liver) | Pork |  |
|  |  |  | pork (carcass) | Pork |  |
|  |  |  | pork (m. Longissimus dorsi) | Pork |  |
| 183 | BS | United Kingdom | beef (m. Longissimus dorsi) | Beef | GC |
| 188 | EX | Sweden | pork (m. Longissimus dorsi) | Pork |  |
| 197 | EX | Sweden | beef (forearm) | Beef |  |
|  |  |  | cattle (kidney, liver) | Beef |  |
| 199 | BS | USA | chicken (breast, thigh) | Chicken meat | GC |
|  |  |  | chicken (breast, thigh, skin) raw | Chicken meat |  |
| 200 | EX | Republic of Korea | pork (m. Longissimus dorsi) | Pork | GC |
| 209 | EX | Spain | rabbit meat (hind leg) | Rabbit meat | NIRS |
| 235 | BS | Italy | beef (m. Longissimus dorsi) | Beef |  |
| 268 | EX | Germany | chicken (breast, thigh) | Chicken meat |  |
|  |  |  | chicken (carcass) | Chicken meat |  |
| 280 | EX | Belgium | pork (m. Longissimus dorsi) | Pork |  |
| 349 | EX | Sweden | pig (kidney, liver) | Pork |  |
| 355 | EX | Spain | rabbit meat (hind leg) | Rabbit meat | NIRS |
| 397 | EX | Spain | beef (meat) | Beef |  |
| 407 | CF | Italy | beef (8th rib) | Beef | GC† |
|  |  |  | beef (m. Longissimus thoracis) | Beef |  |
| 466 | EX | South Africa | beef (m. Longissimus thoracis) | Beef |  |
| 469 | CF | Spain | cattle (kidney, liver) | Beef |  |
| 485 | CF | Spain | beef (diaphragm) | Beef |  |
| 516 | EX | Slovenia | pork (m. Longissimus dorsi) | Pork | GC |
| ID, Paper unique identification number (see Table S1 for references); ST, Study type (CF – Comparison of Farms, BS – Basket Study, EX – Controlled Experiment); AM, analytical method of fatty acids assessment (GC – gas chromatography based, NIRS – NIR-spectroscopy calibrated with GC-data). *Information provided by author, considered as separate datapoints; †Studies which provided only brief descriptions of the methods used to assess fatty acids composition. | | | | | |

| **Table S2 cont.** Study type, location, species/product, animal group and fatty acids analysis method information of the comparison studies included in the meta-analysis. | | | | | |
| --- | --- | --- | --- | --- | --- |
| **ID** | **ST** | **Location** | **Product*** | **Animal group** | **AM** |
| 521 | EX | Turkey | lamb (m. Longissimus dorsi) | Lamb and goat meat |  |
| 529 | BS | Uruguay | beef (short loin, rump loin, rib) | Beef | GC |
| 560 | EX | Italy | lamb (m. Longissimus dorsi) | Lamb and goat meat | GC† |
| 561 | EX | Belgium | pork (m. Longissimus thoracis) | Pork |  |
| 562 | BS | USA | beef (meat) | Beef |  |
|  |  |  | cattle (fat) | Beef |  |
|  |  |  | cattle (kidney, liver) | Beef |  |
| 563 | EX | Poland | pork (m. Adductor, m. Longissimus dorsi) | Pork | GC |
| 564 | EX | Sweden | pork (m. Longissimus dorsi) | Pork | GC |
| 566 | EX | Spain | lamb (m. Longissimus dorsi) | Lamb and goat meat |  |
| 567 | EX | Germany | lamb (m. Longissimus dorsi) | Lamb and goat meat | GC |
| 568 | BS | United Kingdom | chicken (breast, m. Pectoralis major) | Chicken meat |  |
| 569 | BS | United Kingdom | chicken (breast) raw | Chicken meat | GC |
| 570 | EX | Poland | pig (back fat) | Pork | GC† |
| 573 | BS | United Kingdom | beef (m. Longissimus dorsi) | Beef | GC |
| 574 | EX | France | lamb (m. Longissimus thoracis et lumborum) | Lamb and goat meat | GC |
| 578 | BS | Italy | cold meat (salami, dry ham, cooked ham) | Not specified |  |
| 603 | EX | Spain | lamb (m. Longissimus lumborum) | Lamb and goat meat | GC |
| 604 | CF | Poland | beef (m. Longissimus thoracis) | Beef | GC |
| 605 | EX | Brazil | lamb (hamburger) | Lamb and goat meat |  |
| 606 | EX | Brazil | lamb (m. Longissimus dorsi) | Lamb and goat meat | GC† |
| 633 | EX | Spain | goat meat (m. Longissimus thoracis) | Lamb and goat meat | GC |
|  |  |  | goat (pelvic and kidney fat) | Lamb and goat meat |  |
| 634 | EX | Spain | goat meat (m. Longissimus thoracis) | Lamb and goat meat |  |
|  |  |  | goat (pelvic and kidney fat) | Lamb and goat meat |  |
| 635 | BS | United Kingdom | chicken (breast, leg) cooked | Chicken meat | GC |
| 636 | BS | USA | chicken (breast) raw | Chicken meat |  |
|  |  |  | turkey (breast) raw | Turkey meat |  |
| 637 | BS | Poland | pork (m. Longissimus dorsi) | Pork | GC |
| 638 | CF | Denmark | chicken (breast) raw | Chicken meat |  |
| ID, Paper unique identification number (see Table S1 for references); ST, Study type (CF – Comparison of Farms, BS – Basket Study, EX – Controlled Experiment); AM, analytical method of fatty acids assessment (GC – gas chromatography based, NIRS – NIR-spectroscopy calibrated with GC-data). *Information provided by author, considered as separate datapoints; †Studies which provided only brief descriptions of the methods used to assess fatty acids composition. | | | | | |

| **Table S2 cont.** Study type, location, species/product, animal group and fatty acids analysis method information of the comparison studies included in the meta-analysis. | | | | | | | |
| --- | --- | --- | --- | --- | --- | --- | --- |
| **ID** | | **ST** | | **Location** | **Product*** | **Animal group** | **AM** |
| 640 | EX | | Italy | | rabbit meat (carcass) | Rabbit meat | GC |
|  |  | |  | | rabbit meat (m. Longissimus lumborum) | Rabbit meat |  |
| 641 | | EX | | Republic of Korea | chicken (breast) raw | Chicken meat | GC |
| 642 | | BS | | USA | chicken (breast) cooked | Chicken meat |  |
| 644 | | EX | | Sweden | pork (m. Longissimus dorsi) cooked | Pork |  |
|  | |  | |  | pork (m. Longissimus dorsi) raw | Pork |  |
| 645 | | BS | | Brazil | chicken (breast) raw | Chicken meat |  |
| 646 | | EX | | Italy | beef (m. Longissimus thoracis) | Beef |  |
| 647 | | EX | | Germany | pork (meat) | Pork | GC |
| 648 | | EX | | Turkey | chicken (breast, thigh) | Chicken meat | GC |
| 649 | | EX | | Italy | lamb (m. Longissimus dorsi) | Lamb and goat meat | GC |
| 650 | | EX | | Turkey | lamb (m. Semitendinosus) | Lamb and goat meat |  |
|  | |  | |  | sheep (liver) | Lamb and goat meat |  |
| 651 | | BS | | Switzerland | beef (m. Longissimus dorsi) | Beef | GC |
| 652 | | EX | | Spain | lamb (m. Longissimus dorsi) | Lamb and goat meat |  |
| 654 | | BS | | Spain | pork (cooked ham) | Pork |  |
| 655 | | EX | | Brazil | lamb (m. Longissimus dorsi) | Lamb and goat meat |  |
| 657 | | EX | | Spain | beef (m. Longissimus thoracis) | Beef | GC |
| 658 | | EX | | USA | cattle (back fat) | Beef |  |
| 659 | | BS | | United Kingdom | chicken (breast) | Chicken meat | GC |
| 660 | | EX | | Croatia | chicken (meat) | Chicken meat |  |
| 661 | | EX | | Taiwan | goat meat (m. Longissimus dorsi) | Lamb and goat meat | GC |
| 662 | | EX | | Croatia | chicken (breast) | Chicken meat |  |
| ID, Paper unique identification number (see Table S1 for references); ST, Study type (CF – Comparison of Farms, BS – Basket Study, EX – Controlled Experiment); AM, analytical method of fatty acids assessment (GC – gas chromatography based, NIRS – NIR-spectroscopy calibrated with GC-data). *Information provided by author, considered as separate datapoints; †Studies which provided only brief descriptions of the methods used to assess fatty acids composition. | | | | | | | |

| **Table S3.** Production systems information for studies with more than two systems. | | | | |  |
| --- | --- | --- | --- | --- | --- |
| **ID** | **Species** | **SN** | **Production system** | **Additional comparisons used in the sensitivity analyses 2 and 3*** | |
| 167 | pig | 1 | organic (100% concentrate fed) | 1 and 4 | |
|  |  | 2 | organic (70% concentrate + 30% barley/pea silage fed)† |  | |
|  |  | 3 | organic (70% concentrate + 30% clover/grass silage fed)† |  | |
|  |  | 4 | conventional (100% concentrate fed)‡ |  | |
| 268 | chicken | 1 | organic‡ | 1 and 3 | |
|  |  | 2 | conventional (intensive)‡ |  | |
|  |  | 3 | conventional (free range) |  | |
| 466 | cattle | 1 | organic‡ | 1 and 3 | |
|  |  | 2 | conventional (intensive)‡ |  | |
|  |  | 3 | conventional (pasture) |  | |
| 469 | cattle | 1 | organic‡ | 1 and 3 | |
|  |  | 2 | conventional‡ |  | |
|  |  | 3 | conventional (intensive) |  | |
| 485 | cattle | 1 | organic‡ | 1 and 3 | |
|  |  | 2 | conventional‡ |  | |
|  |  | 3 | conventional (intensive) |  | |
| 568 | chicken | 1 | organic‡ | 1 and 3 | |
|  |  | 2 | conventional (standard)‡ | 1 and 4 | |
|  |  | 3 | conventional (maize fed) |  | |
|  |  | 4 | conventional (free range) |  | |
| ID, Paper unique identification number (see Table S1 for references); SN, number of the system. *Numbers refer to the SN within the same study; †Results from these treatments were averaged and used as a standard organic system in the meta-analysis; ‡Used as a standard system in the standard meta-analysis and sensitivity meta-analysis 1. | | | | |  |

| **Table S3 cont.** Production systems information for studies with more than two systems. | | | | |  |
| --- | --- | --- | --- | --- | --- |
| **ID** | **Species** | **SN** | **Production system** | **Additional comparisons used in the sensitivity analyses 2 and 3*** | |
| 569 | chicken | 1 | organic‡ | 1 and 3 | |
|  |  | 2 | conventional‡ |  | |
|  |  | 3 | conventional (free range) |  | |
| 570 | pig | 1 | organic (fed with limited mixture composed of organically grown cereals, legume seeds and rapeseed cake + ad libitum maize silage)† |  | |
|  |  | 2 | organic (fed with limited mixture composed of organically grown cereals, legume seeds and rapeseed cake + ad libitum maize silage + 0.5% supplement of a herb mixture)† |  | |
|  |  | 3 | organic (fed with limited mixture composed of organically grown cereals, legume seeds and rapeseed cake + ad libitum grass silage)† |  | |
|  |  | 4 | organic (fed with limited mixture composed of organically grown cereals, legume seeds and rapeseed cake + ad libitum grass silage + 0.5% supplement of a herb mixture)† |  | |
|  |  | 5 | conventional (soya-based mixture fed) |  | |
| 640 | rabbit | 1 | organic (local breed)‡ | 1 and 3 | |
|  |  | 2 | conventional (local breed)‡ |  | |
|  |  | 3 | conventional (commercial hybrid) |  | |
| 642 | chicken | 1 | organic‡ | 1 and 3 | |
|  |  | 2 | conventional‡ |  | |
|  |  | 3 | conventional (antibiotic free) |  | |
| ID, Paper unique identification number (see Table S1 for references); SN, number of the system. *Numbers refer to the SN within the same study; †Results from these treatments were averaged and used as a standard organic system in the meta-analysis; ‡Used as a standard system in the standard meta-analysis and sensitivity meta-analysis 1. | | | | |  |

| **Table S3 cont.** Production systems information for studies with more than two systems. | | | | |  |
| --- | --- | --- | --- | --- | --- |
| **ID** | **Species** | **SN** | **Production system** | **Additional comparisons used in the sensitivity analyses 2 and 3*** | |
| 645 | chicken | 1 | organic‡ | 1 and 3 | |
|  |  | 2 | conventional‡ |  | |
|  |  | 3 | conventional (free range) |  | |
| 647 | pig | 1 | organic (mainly concentrates fed)‡ | 2 and 4 | |
|  |  | 2 | organic (concentrates fed, partly replaced by grass cobs) | 3 and 4 | |
|  |  | 3 | organic (concentrates fed, partly replaced by grass silage) |  | |
|  |  | 4 | conventional‡ |  | |
| 648 | chicken | 1 | organic (slow growing)‡ | 1 and 3 | |
|  |  | 2 | conventional (slow growing)‡ |  | |
|  |  | 3 | conventional (fast growing) |  | |
| 649 | lamb | 1 | organic (low stocking)‡ |  | |
|  |  | 2 | conventional (low stocking)‡ |  | |
|  | lamb | 1 | organic (high stocking)‡ |  | |
|  |  | 2 | conventional (high stocking)‡ |  | |
| 658 | cattle | 1 | organic (pasture + concentrate fed)‡ | 2 and 3 | |
|  |  | 2 | organic (grass fed) |  | |
|  |  | 3 | conventional‡ |  | |
| 661 | goat | 1 | organic (dry mulberry leave fed) | 1 and 3 | |
|  |  | 2 | organic (alfalfa hay fed)‡ |  | |
|  |  | 3 | conventional‡ |  | |
| ID, Paper unique identification number (see Table S1 for references); SN, number of the system. *Numbers refer to the SN within the same study; †Results from these treatments were averaged and used as a standard organic system in the meta-analysis; ‡Used as a standard system in the standard meta-analysis and sensitivity meta-analysis 1. | | | | |  |

| **Table S4.** Information extracted from the papers and included in the database used for meta-analysis. | |
| --- | --- |
| **Information  about the paper** | Paper ID, authors, publication year, title, journal/publisher, type of paper (journal article, conference proceedings, conference paper, report, book chapter, thesis), corresponding author, language of publication, information about peer-review, source of paper (electronic databases, contact with authors, reference list of reviews and original publications). |
| **Study characteristics** | Study type (Controlled Experiment - EX, Comparison of Farms - CF, Basket Study - BS), product, species, breed, production system description, experimental year(s), location of the study by country*, analytical methods used. |
| **Data** | Name of the compositional parameter, number of replicates, mean, SE or SD, measurement unit, data type (numeric, graphical). |
| *Country codes according ISO 3166-2 (see [*http://www.iso.org/iso/home/standards/country_codes.htm*](http://www.iso.org/iso/home/standards/country_codes.htm)) | |

| **Table S5.** Summary of inclusion criteria used in the standard and the sensitivity analyses carried out. Detailed results of the sensitivity analysis are shown in the Appendix on the Newcastle University website ([*http://research.ncl.ac.uk/nefg/QOF*](http://research.ncl.ac.uk/nefg/QOF)) | | | | | | | | | |
| --- | --- | --- | --- | --- | --- | --- | --- | --- | --- |
| **Analysis** | **Data available** | |  | **Production systems compared** | |  | **20% of studies with the least precise treatment effects excluded** |  | **Studies considered to have less scientifically sound methods of fatty acids assessments excluded**† |
|  | Only papers  reporting N, mean, SD/SE | All papers  reporting means |  | Standard organic  with standard conventional* | Each organic  with each conventional |  |  |  |  |
| Standard‡ |  |  |  |  |  |  |  |  |  |
| WM | + |  |  | + |  |  |  |  |  |
| Sensitivity§ |  |  |  |  |  |  |  |  |  |
| 1 (UM)‡ |  | + |  | + |  |  |  |  |  |
| 2 (WM) | + |  |  |  | + |  |  |  |  |
| 3 (UM) |  | + |  |  | + |  |  |  |  |
| 4 (WM) | + |  |  | + |  |  | + |  |  |
| 5 (WM) | + |  |  | + |  |  |  |  | + |
| *A pragmatic choice was made to compare organic with a standard conventional comparator; †Five studies which provided only brief descriptions of the methods used (see Supplementary Table S3 and S2); ‡Results of the standard meta-analysis and sensitivity analysis 1 are presented in the main paper; §Sensitivity analysis was conducted to explore the robustness of the arbitrary decisions and to illustrate all effects (see Supplementary Table S3 for details and Appendix Table A1-3 for results of sensitivity analysis 2-5). WM, weighted meta-analysis; UM, unweighted meta-analysis. | | | | | | | | | |

| **Table S6.** List of composition parameters included in the statistical analyses.* | |
| --- | --- |
| **Category** | **Parameters** |
| **Major components** | Ash, Dry mass, Fat, Intramuscular fat, Protein, Water |
| **Fatty acids** | 10:0 (capric acid), 12:0 (lauric acid), 14:0 (myristic acid), 14:1, 15:0 (pentadecanoic acid), 16:0 (palmitic acid), 16:1 (palmitoleic acid), 16:1 n-7, 16:1 n-9, 17:0 (heptadecanoic acid), 17:1 (heptadecenoic acid), 18:0 (stearic acid), 18:1 n-7, 20:0 (arachidic acid), 20:1 n-9, 20:2, 21:0, 22:0, 23:0 (cerotic acid), 24:0 (lignoceric acid), AA (cis-5,8,11,14-20:4), ALA (cis-9,12,15-18:3), cis-11,14-20:2 n-6, cis-11-20:1 (eicosenoic acid), CLA (cis-9-trans-11-18:2), CLA (total), CLA (trans-10-cis-12-18:2), CLA index, DGLA (cis-8,11,14-20:3), DHA (cis-4,7,10,13,16,19-22:6), DPA (cis-7,10,13,16,19-22:5), DTA (cis-7,10,13,16-22:4), EPA (cis-5,8,11,14,17-20:5), EPA+DHA†, ETE (cis-11,14,17-20:3), GLA (cis-6,9,12-18:3), LA (cis-9,12-18:2), LA/ALA ratio†, MUFA, n-3 FA, n-3/n-6 ratio, n-6 FA, n-6/n-3 ratio, OA (cis-9-18:1), PUFA, PUFA/SFA ratio, SFA, trans-18:1 (total), trans-18:1 n-9, trans-9-18:1, USFA, USFA/SFA ratio, VA (trans-11-18:1), VLC n-3 PUFA (EPA+DPA+DHA)†, Δ-9 desaturase 16:1/16:0 activity index, Δ-9 desaturase 18:1/18:0 activity index |
| **Vitamins and antioxidants** | α-tocopherol (total) |
| **Minerals and undesirable metals** | Arsenic (As), Cadmium (Cd), Copper (Cu), Iron (Fe), Iron (Fe) (in haemoglobin), Lead (Pb), Selenium (Se), Zinc (Zn) |
| **Pesticides, mycotoxins  and other contaminants** | 4-4’-DDD, 4-4’-DDE, 4-4’-DDT, Aldrin, Chlorpyrifos, Diazinon, Dieldrin, Disyston, Endrin, Ethion, Ethyl parathion, Heptachlor, Hexachlorobenzene (HCB), Lindane, Malathion, Methoxychlor, Methyl parathion, Mirex, Pirimiphos-Me, Ronnel, Trithion, α-benzene hexachloride (α-BHC), β-benzene hexachloride (β-BHC), δ-benzene hexachloride (δ-BHC) |
| **Other** | Atherogenicity Index (AI), Atherogenicity Index (AI)†, Campylobacter spp., Cholesterol, Lipid oxidation (TBARS), pH, Thrombogenicity index (TI), Thrombogenicity Index (TI)† |
| *Compounds for which number of comparisons organic vs. conventional was ≥ 3, †Calculated based on published fatty acids composition data. | |

| **Table S7.** List of composition parameters excluded from the statistical analyses.* | |
| --- | --- |
| **Category** | **Parameters** |
| **Major components** | Abdominal fat, Carbohydrate, Intra-abdominal fat, Meat weight (hind leg), Meat weight (loin weight), Meat/bone ratio |
| **Fatty acids** | 11:0, 13:0, 14:1 n-6, 15:1, 18:1 (total), 18:1 n-12, 18:1 n-5, 18:2, 18:3, 18:4 n-3, 19:0, 20:1 (eicosanoic acid), 20:1 (gadoleic acid), 20:3, 20:3 n-6, 20:4, 22:1, 22:2, 22:2 n-6, 22:4 n-3, 24:1 n-9, 25:1, 3S,7R,11R,15-phytanic acid (SRR), 6:0 (caproic acid), 8:0 (caprylic acid), AA/EPA ratio, anteiso-15:0, branched-15:0, branched-16:0, branched-17:0, cis-11-18:1 (cis-vaccenic acid), cis-12,15-18:2, cis-12-18:1, cis-13-18:1, cis-14,trans-16-18:1, cis-15-18:1, cis-18:1 (total), cis-8-20:1, cis-9,15-18:2, cis-9-trans-12-18:2 + trans-9,12-18:2, cis-9-trans-13-18:2, cis-MUFA, cis-PUFA, CLA (cis-9,11-18:2), DPA (cis-7,10,13,16,19-22:5), iso-14:0, iso-15:0, iso-16:0, iso-17:0, LA/ALA ratio, LCFA, MCFA, MUFA/SFA ratio, Other FA, SCFA, trans-10-18:1, trans-10-18:1 + trans-11-18:1, trans-11-cis15-18:2, trans-12,13,14-18:1, trans-16:1 (trans-palmitelaidic acid), trans-16-18:1, trans-18:2 n-6, trans-6,7,8-18:1, trans8-cis-13-18:2, trans-9,11-18:2, trans-9,12-18:2, trans-9-16:1, trans-FA, Triglycerides |
| **N components** | Alanine (Ala), Ammonia, Arginine (Arg), Arginine (Arg) + Threonine (Thr), Aspartic acid (Asp), Cadaverine, Carnosine, Citrulline, Creatine, Cysteine (Cys), Dipeptides (total), Free Amino Acids (FAA) total, Glutamic acid (Glu), Glycine (Gly), Histidine (His), Isoleucine (Ile), Leucine (Leu), Lysine (Lys), Methionine (Met), Nitrate, Nitrite, Ornithine, Phenylalanine (Phe), Proline (Pro), Serine (Ser), Taurine, Threonine (Thr), Tryptophan (Trp), Tyrosine (Tyr), Valine (Val), β-Alanine |
| **Vitamins and antioxidants** | Anserine, Glutathione (GSH), Retinol, β-carotene, γ-tocopherol |
| **Minerals and undesirable metals** | Calcium (Ca), Chromium (Cr), Cobalt (Co), Magnesium (Mg), Manganese (Mn), Mercury (Hg), Molybdenum (Mo), Nickel (Ni), Other Arsenic species, Phosphorus (P), Potassium (K), Sodium (Na) |
| **Pesticides, mycotoxins  and other contaminants** | 2-amino-3,4,8-trimethylimidazo[4,5-f]quinoxaline (4,8-DiMeIQx), 3-amino-2-oxazolidinone, carbonyls, chlorinated hydrocarbon pesticides, cis-clordane, cis-clordene, Dimethyl arsinate (DMA), Eptachlor, Esachlorciclohexane (HCH), Heptachlor epoxide, o,p'DDD, o,p'DDE, o,p'DDT, Octaclorostyrene, Organophosphate pesticides, Ossiclordane, p,p'DDD, p,p'DDE, Pesticide residues, Polychlorinated biphenyls (PCB), Quintozene, Roxarsone, trans-clordane, trans-clordene, trans-nonachlor, α-endosulfan |
| *Compounds for which number of comparisons organic vs. conventional was < 3. | |

| **Table S7 cont.** List of composition parameters excluded from the statistical analyses.* | |
| --- | --- |
| **Category** | **Parameters** |
| **Volatile compounds** | 1,2-Propanediol, 1,8-cineole, 1.19-eicosadienoic acid, 1-dodecanol, 1-heptanol,  1-hexanedecanol, 1-hydroxy-2-propanone, 1-nonene, 1-octen-3-ol, 1-octene,  1-pentanol, 1-propanol, 1-tetradecanol, 1-tridecanol, 2,2,4,6,6-pentametilheptanal, 2,3-octanedione, 2,3-pentanedione, 2/3-methylthiophene + isobutyl acetate,  2-butanone, 2-ethyl-1-hexanol, 2-ethylfuran, 2-heptanone, 2-hexanone, 2-isobutyl-4-methylpyridine, 2-methyl-3-pentanone, 2-methylbutanal, 2-methylbutanoic acid,  2-methyl-dihydro-3(H)-furanone, 2-methylpropanal, 2-nonenal, 2-octanone,  2-pentanone, 2-pentyl, 2-pentylfuran, 2-phenoxyethanol, 2-propanol, 2-undecenal,  3-hydroxy-2-butanone, 3-methyl-1-butanol, 3-methyl-2-butenal, 3-methylbutanal,  3-methylbutanenitrile, 3-methylbutanoic acid, 3-methylhexane, 3-methylnonane,  3-phenylpropionitrile, 4-methyl-3-penten-2-one, 4-methylnonane,  4-methylpentanenitrile, Acetic acid, Benzaldehyde, Benzamide, Benzoic acid, Benzophenone, Benzothiazole, Benzylcyanide, Butyl acetate, Cyclohexanone, Decanal, Decanenitrile, Dimethyl disulphide, Dimethyl tetrasulphide, Dimethyl trisulphide, Dodecanal, Dodecanoic acid, Ethanol, Ethyl acetate, Formaldehyde, Furfural, Heptanal, Heptane, Heptanol, Hexadecanal, Hexanal, Hexanoic acid, Hexanol, Indole, Isobutyramide, Limonene, Methylcyclohexane, Methylpyrazine, n-formylmorpholine, n-formylpiperidine, n-heptanal, n-methylbenzamide, Nonanal, Nonanoic acid, Octanal, Octane, Pentanal, Pentanoic acid, Propanoic acid, Pyrazine, Pyrrole, Tetradecanal, Tetradecane, Tetradecanenitrile, Tetradecanoic acid, Thiazole, Thiophene, Tridecane, Undecanal, Xylene, γ-aminobutyric lactam |
| **Other** | 1-methyl-9H-pyrido[3,4-b]indole (harmane), 2-amino-1-methyl-6-phenylimidazo-[4,5-b]pyridine (PhIP), 2-amino-3,8-dimethylimidazo[4,5-f]quinoxaline (MeIQx),  9H-pyrido[3,4-b]indole (norharmane), Aerobic bacteria, Catalase, E. coli, Ether extract, Glutathione peroxidase (GPx), Glutathione reductase (GR), Glycogen (residual), Gross energy, Heterocyclic amines (HCAs) (total), Metmyoglobin, NaCl, Phosphates, Putrescine, Staphylococcus spp., Total coliforms |
| *Compounds for which number of comparisons organic vs. conventional was < 3. | |

# 2. ADDITIONAL METHODS

The methods for random-effects model used in weighted meta-analysis were previously described by Baranski *et al*.^(^[^1^](#_ENREF_1)^)^.

## **2.1.** Calculations used for weighted meta-analyses

The SMD from a single study was calculated in random-effect model using standard formulas within “metafor” as follows:

$$SMD= \frac{\bar{X}_{O}- \bar{X}_{C}}{S_{within}} \times J$$

where *X̅o* is the mean value for experimental group (organic), *X̅*_C_ is the mean value for control group (conventional), *S_within_* is the pooled standard deviation of the two groups, and *J* is a factor used to correct for small sample size. *J* is calculated as:

$$J=1- \frac{3}{4\times(n_{C}+n_{O}-2)-1}$$

where *n_O_* and *n_C_* are organic and conventional sample sizes.

*S_within_* is calculated as:

$$S_{within}=\sqrt{\frac{\left( n_{O}-1 \right)S_{O}^{2}+\left( n_{C}-1 \right)S_{C}^{2}}{n_{O}+n_{C}-2}}$$

where *S_O_* and *S_C_* are the standard deviations in individual systems (organic and conventional) respectively.

The pooled SMD (SMD_tot_) across all studies was calculated as:

$${SMD}_{tot}= \frac{\sum_{i=1}^{n} (\frac{1}{v_{i}}\times{SMD}_{i})}{\sum_{i=1}^{n} (\frac{1}{v_{i}})}$$

Where *v_i_* is a sampling variance estimated as:

$$v_{i}=\frac{n_{C}+n_{O}}{n_{C}\times n_{O}}+ \frac{{SMD}^{2}}{2\times\left( n_{C}+n_{O} \right)}$$

The pooled or summary effect (SMD_tot_) was calculated for all nutrient- and composition-related parameters reported in a minimum of 3 studies, following procedures advocated by Lipsey and Wilson^(^[^2^](#_ENREF_2)^)^.

## **2.2.** Calculations used for percentage mean differences (MPDs)

For each data-pair (*X̅_O_*, *X̅_C_*) extracted from the literature and used in the meta-analysis the percentage difference was calculated as:

$+\left[ \left( {\bar{X}_{O}\times100}/{\bar{X}_{C}} \right)-100 \right]$ for data sets where *X̅_O_>X̅_C_*, or

$-\left[ \left( {\bar{X}_{C}\times100}/{\bar{X}_{O}} \right)-100 \right]$ for data sets where *X̅_C_>X̅_O_*

# 3. ADDITIONAL RESULTS

Supplementary Table S8 shows the basic information/statistics on the publications/data used for meta-analyses of composition parameters included in Fig. 2-4 in the main paper.

Supplementary Table S9 and S10 shows the mean percentage differences (MPD) and standard errors (SE) calculated using the data included in for standard and sensitivity 1 meta-analyses of composition parameters shown in Fig. 2-4 of the main paper (MPDs are also shown as symbols in Fig. 2-4).

Supplementary Table S11 shows the meta-analysis results for addition composition parameters (protein, 20:0 (arachidic acid), 14:1, 16:1 (palmitoleic acid), 17:1 (heptadecenoic acid), CLA (cis-9-trans-11-18:2), PUFA/SFA ratio, *n*-3/*n*-6 ratio, EPA+DHA, atherogenicity index, thrombogenicity index, cholesterol, lipid oxidation (TBARS)) for which significant differences were detected by the standard and sensitivity 1 meta-analyses protocols.

Supplementary Figures S3 to S5 show forest plots and the results of the standard and sensitivity 1 meta-analyses random-effect and mixed-effect models with study type as moderator, for data from studies which compared the composition of organic and conventional animal products.

Supplementary Figures S6 to S35 show forest plots comparing SMDs from standard meta-analysis random-effect and mixed-effect models for different animal groups, for composition parameters for which significant difference between organic and conventional animal products were found by one of the meta-analyses protocols.

Supplementary Table S12 shows the results of the standard and sensitivity 1 meta-analyses for parameters where none of the meta-analyses protocols detected significant differences between organic and conventional meat.

Supplementary Table S13 shows the results of the statistical tests for publication bias ported in Table 1 of the main paper.

| **Table S8.** Basic information/statistics on the publications/data used for meta-analyses of composition parameters included in Fig. 2 in the main paper. | | | | | | | | | | |
| --- | --- | --- | --- | --- | --- | --- | --- | --- | --- | --- |
|  |  |  |  |  | **Number of comparisons reporting that concentrations were** | | | | | |
|  |  |  | **Total sample size*** | | **numerically higher in** | | **identical** | **significantly higher in** | | **not significantly different**§ |
| **Parameter** | **Studies** | ***n*** | **ORG** | **CONV** | **ORG** | **CONV** |  | **ORG**† | **CONV**‡ |  |
| Fat | 31 | 34 | 622 | 618 | 8 | 25 | 1 | 2 | 8 | 11 |
| Intramuscular fat | 9 | 9 | 207 | 215 | 3 | 6 | 0 | 1 | 4 | 1 |
| SFA | 34 | 38 | 725 | 704 | 18 | 20 | 0 | 2 | 7 | 11 |
| 12:0 (lauric acid) | 13 | 15 | 261 | 234 | 7 | 7 | 1 | 1 | 1 | 8 |
| 14:0 (myristic acid) | 25 | 27 | 450 | 449 | 10 | 16 | 1 | 1 | 5 | 10 |
| 16:0 (palmitic acid) | 28 | 30 | 511 | 508 | 13 | 17 | 0 | 0 | 3 | 13 |
| MUFA | 32 | 36 | 706 | 690 | 10 | 26 | 0 | 1 | 9 | 9 |
| OA (cis-9-18:1) | 25 | 27 | 482 | 483 | 11 | 16 | 0 | 2 | 4 | 10 |
| PUFA | 31 | 35 | 688 | 672 | 28 | 6 | 1 | 10 | 1 | 7 |
| n-3 FA | 27 | 31 | 557 | 537 | 24 | 7 | 0 | 9 | 0 | 5 |
| ALA (cis-9,12,15-18:3) | 27 | 32 | 477 | 449 | 23 | 9 | 0 | 6 | 1 | 8 |
| EPA (cis-5,8,11,14,17-20:5)\|\| | 19 | 23 | 348 | 329 | 12 | 11 | 0 | 4 | 1 | 4 |
| DPA (cis-7,10,13,16,19-22:5) | 12 | 15 | 290 | 257 | 10 | 5 | 0 | 4 | 0 | 4 |
| DHA (cis-4,7,10,13,16,19-22:6) | 19 | 23 | 348 | 329 | 13 | 9 | 1 | 5 | 0 | 4 |
| VLC n-3 PUFA (EPA+DPA+DHA)¶ | 12 | 15 | 290 | 257 | 11 | 4 | 0 | - | - | - |
| n-6 FA | 25 | 29 | 534 | 507 | 19 | 10 | 0 | 6 | 0 | 8 |
| LA (cis-9,12-18:2) | 2 | 2 | 29 | 31 | 1 | 1 | 0 | 0 | 0 | 1 |
| AA (cis-5,8,11,14-20:4)\|\| | 21 | 24 | 363 | 344 | 11 | 13 | 0 | 2 | 3 | 7 |
| LA/ALA ratio¶ | 25 | 28 | 428 | 420 | 10 | 18 | 0 | - | - | - |
| n-6/n-3 ratio | 28 | 32 | 612 | 590 | 10 | 22 | 0 | 1 | 5 | 8 |
| *n*, numbers of data points (comparisons) included in the meta-analysis; ORG, organic samples; CONV, conventional samples; SFA, saturated fatty acids; MUFA, monounsaturated fatty acids; OA, oleic acid; PUFA, polyunsaturated fatty acids; FA, fatty acids; ALA, α-linolenic acid; EPA, eicosapentaenoic acid; DPA, docosapentaenoic acid; DHA, docosahexaenoic acid; VLC n-3 PUFA, very long chain n-3 PUFA; LA, linoleic acid; AA, arachidonic acid. *Total number of samples analysed in different publications; †The number of comparisons in which statistically significant difference was found with higher level in ORG; ‡The number of comparisons in which statistically significant difference was found with higher level in CONV; §The number of comparisons in which there was no significant difference between ORG and CONV; \|\|Outlying data pairs (where the MPD between ORG and CONV was over fifty times greater than the mean value including the outliers) were removed; ¶Calculated based on published fatty acids composition data. | | | | | | | | | | |

| **Table S9.** Mean percentage differences (MPD) and confidence intervals (CI) calculated using the data included in standard meta-analyses and sensitivity meta-analysis 1 of composition parameters shown in Fig. 2 of the main paper (MPDs are also shown as symbols in Fig. 2). | | | | | | | |
| --- | --- | --- | --- | --- | --- | --- | --- |
|  | **Calculated based on data included in** | | | | | | |
|  | **standard meta-analysis** | | |  | **sensitivity meta-analysis 1** | | |
| **Parameter** | ***n*** | **MPD*** | **95% CI** |  | ***n*** | **MPD*** | **95% CI** |
| Fat | 22 | -22.21 | -43.92, -0.51 |  | 34 | -21.46 | -40.34, -2.59 |
| Intramuscular fat | 7 | -12.40 | -37.76, 12.97 |  | 9 | -21.73 | -45.58, 2.12 |
| SFA | 26 | -2.37 | -5.69, 0.94 |  | 38 | -1.67 | -4.17, 0.83 |
| 12:0 (lauric acid) | 11 | 4.94 | -21.44, 31.31 |  | 15 | 7.94 | -12.61, 28.48 |
| 14:0 (myristic acid) | 23 | -18.35 | -31.97, -4.72 |  | 27 | -18.11 | -30.25, -5.97 |
| 16:0 (palmitic acid) | 24 | -10.85 | -27.67, 5.98 |  | 30 | -8.50 | -22.03, 5.02 |
| MUFA | 24 | -7.97 | -12.47, -3.48 |  | 36 | -6.55 | -10.01, -3.09 |
| OA (cis-9-18:1) | 22 | -3.71 | -8.43, 1.01 |  | 27 | -4.91 | -9.16, -0.66 |
| PUFA | 23 | 23.29 | 11.27, 35.31 |  | 35 | 18.90 | 7.28, 30.51 |
| n-3 FA | 21 | 46.99 | 10.08, 83.89 |  | 31 | 38.38 | 12.16, 64.61 |
| ALA (cis-9,12,15-18:3) | 22 | 17.00 | -11.49, 45.49 |  | 32 | 35.08 | 1.34, 68.82 |
| EPA (cis-5,8,11,14,17-20:5)† | 13 | 0.93 | -37.51, 39.37 |  | 20 | -6.11 | -35.79, 23.56 |
| DPA (cis-7,10,13,16,19-22:5) | 11 | 30.45 | -0.18, 61.07 |  | 15 | 29.49 | 7.07, 51.91 |
| DHA (cis-4,7,10,13,16,19-22:6) | 14 | 13.84 | -35.39, 63.07 |  | 22 | 8.63 | -23.91, 41.18 |
| VLC n-3 PUFA (EPA+DPA+DHA)‡ | - | - | - |  | 15 | 24.20 | 3.57, 44.83 |
| n-6 FA | 19 | 16.34 | 1.73, 30.94 |  | 29 | 12.57 | 1.92, 23.22 |
| LA (cis-9,12-18:2) | 23 | 8.53 | -11.48, 28.55 |  | 30 | 9.69 | -7.07, 26.44 |
| AA (cis-5,8,11,14-20:4)† | 13 | 11.67 | -8.16, 31.50 |  | 19 | 1.40 | -14.68, 17.47 |
| LA/ALA ratio‡ | - | - | - |  | 28 | -20.43 | -40.41, -0.45 |
| n-6/n-3 ratio | 17 | -21.98 | -46.56, 2.60 |  | 32 | -27.71 | -48.05, -7.38 |
| *n*, number of data points included in the comparison; MPD, mean percentage difference; SFA, saturated fatty acids; MUFA, monounsaturated fatty acids; OA, oleic acid; PUFA, polyunsaturated fatty acids; FA, fatty acids; ALA, α-linolenic acid; EPA, eicosapentaenoic acid; DPA, docosapentaenoic acid; DHA, docosahexaenoic acid; VLC n-3 PUFA, very long chain n-3 PUFA; LA, linoleic acid; AA, arachidonic acid. *Magnitude of difference between organic (ORG) and conventional (CONV) samples (value <0 indicate higher concentration in CONV, value >0 indicate higher concentration in ORG); †Outlying data pairs (where the MPD between ORG and CONV was over fifty times greater than the mean value including the outliers) were removed; ‡Calculated based on published fatty acids composition data. | | | | | | | |

| **Table S10.** Mean percentage differences (MPD) and confidence intervals (CI) calculated using the data included in standard meta-analyses and sensitivity meta-analysis 1 of composition parameters shown in Fig. 3 and 4 of the main paper (MPDs are also shown as symbols in Fig. 3 and 4). | | | | | | | |
| --- | --- | --- | --- | --- | --- | --- | --- |
|  | **Calculated based on data included in** | | | | | | |
| **Parameter/** | **standard meta-analysis** | | |  | **sensitivity meta-analysis 1** | | |
| **Animal group*** | ***n*** | **MPD**† | **95% CI** |  | ***n*** | **MPD**† | **95% CI** |
| Fat |  |  |  |  |  |  |  |
| Beef | 6 | -40.10 | -97.52, 17.31 |  | 7 | -35.83 | -85.07, 13.41 |
| Lamb and goat meat | 7 | 11.08 | -5.62, 27.78 |  | 11 | -5.44 | -31.32, 20.43 |
| Pork | - | - | - |  | 5 | -4.64 | -24.65, 15.37 |
| Chicken meat | 4 | -50.04 | -90.14, -9.93 |  | 9 | -31.90 | -79.37, 15.56 |
| Intramuscular fat |  |  |  |  |  |  |  |
| Pork | 4 | 0.31 | -20.30, 20.92 |  | 5 | -6.42 | -27.13, 14.29 |
| SFA |  |  |  |  |  |  |  |
| Beef | 5 | -5.27 | -19.50, 8.95 |  | 8 | -3.92 | -12.70, 4.86 |
| Lamb and goat meat | 9 | -0.56 | -3.60, 2.47 |  | 14 | 0.53 | -2.20, 3.27 |
| Pork | 4 | -2.88 | -7.09, 1.32 |  | 6 | -4.60 | -8.01, -1.18 |
| Chicken meat | 5 | -2.71 | -12.48, 7.05 |  | 7 | -0.99 | -8.09, 6.11 |
| 12:0 (lauric acid) |  |  |  |  |  |  |  |
| Beef | - | - | - |  | 4 | -13.03 | -25.86, -0.20 |
| Lamb and goat meat | 7 | 5.87 | -26.08, 37.82 |  | 9 | 13.15 | -13.70, 40.01 |
| 14:0 (myristic acid) |  |  |  |  |  |  |  |
| Beef | 5 | -23.30 | -43.41, -3.20 |  | 6 | -19.03 | -37.46, -0.60 |
| Lamb and goat meat | 9 | 2.03 | -4.48, 8.53 |  | 11 | 0.42 | -6.35, 7.20 |
| Pork | 4 | -17.40 | -43.87, 9.08 |  | 4 | -17.40 | -43.87, 9.08 |
| Chicken meat | 4 | -65.22 | -109.31, -21.13 |  | 5 | -63.35 | -97.70, -29.00 |
| 16:0 (palmitic acid) |  |  |  |  |  |  |  |
| Beef | 5 | -4.07 | -10.44, 2.30 |  | 7 | -2.55 | -7.35, 2.26 |
| Lamb and goat meat | 9 | -0.18 | -2.25, 1.89 |  | 11 | -0.01 | -1.81, 1.79 |
| Pork | 4 | -3.19 | -7.22, 0.84 |  | 5 | -3.70 | -6.97, -0.42 |
| Chicken meat | 5 | -45.45 | -124.69, 33.79 |  | 6 | -36.73 | -103.65, 30.18 |
| MUFA |  |  |  |  |  |  |  |
| Beef | 4 | -10.84 | -26.15, 4.47 |  | 7 | -7.86 | -16.55, 0.83 |
| Lamb and goat meat | 8 | 1.16 | -2.11, 4.43 |  | 13 | -0.84 | -4.69, 3.02 |
| Pork | 4 | -7.30 | -11.88, -2.73 |  | 6 | -2.98 | -9.33, 3.36 |
| Chicken meat | 5 | -19.67 | -25.82, -13.53 |  | 7 | -17.47 | -23.83, -11.11 |
| *n*, number of data points included in the comparison; MPD, mean percentage difference; SFA, saturated fatty acids; MUFA, monounsaturated fatty acids. *The summary results and product groups for which n≤3 were removed (for summary results see Table S9.), †Magnitude of difference between organic (ORG) and conventional (CONV) samples (value <0 indicate higher concentration in CONV, value >0 indicate higher concentration in ORG). | | | | | | | |

| **Table S10 cont.** Mean percentage differences (MPD) and confidence intervals (CI) calculated using the data included in standard meta-analyses and sensitivity meta-analysis 1 of composition parameters shown in Fig. 3 and 4 of the main paper (MPDs are also shown as symbols in Fig. 3 and 4). | | | | | | | |
| --- | --- | --- | --- | --- | --- | --- | --- |
|  | **Calculated based on data included in** | | | | | | |
| **Parameter/** | **standard meta-analysis** | | |  | **sensitivity meta-analysis 1** | | |
| **Animal group*** | ***n*** | **MPD**† | **95% CI** |  | ***n*** | **MPD**† | **95% CI** |
| OA (cis-9-18:1) |  |  |  |  |  |  |  |
| Beef | 5 | 2.96 | -6.25, 12.18 |  | 6 | 1.83 | -6.01, 9.67 |
| Lamb and goat meat | 9 | -0.74 | -2.71, 1.23 |  | 11 | -2.16 | -5.82, 1.51 |
| Pork | 4 | -2.99 | -11.97, 5.99 |  | 5 | -3.72 | -10.82, 3.38 |
| Chicken meat | - | - | - |  | 4 | -25.71 | -29.06, -22.36 |
| PUFA |  |  |  |  |  |  |  |
| Beef | 4 | 29.43 | 5.58, 53.29 |  | 7 | 18.13 | 1.62, 34.63 |
| Lamb and goat meat | 7 | 8.96 | -0.63, 18.55 |  | 12 | 17.28 | -4.12, 38.68 |
| Pork | 4 | 25.35 | 0.81, 49.88 |  | 6 | 9.33 | -23.91, 42.57 |
| Chicken meat | 5 | 41.74 | -2.69, 86.17 |  | 7 | 32.27 | -0.81, 65.36 |
| n-3 FA |  |  |  |  |  |  |  |
| Beef | - | - | - |  | 7 | 55.42 | 22.50, 88.35 |
| Lamb and goat meat | 8 | 8.54 | -4.19, 21.28 |  | 11 | 11.05 | -2.36, 24.46 |
| Chicken meat | 6 | 66.20 | -16.20, 148.6 |  | 8 | 41.64 | -26.96, 110.24 |
| ALA (cis-9,12,15-18:3) |  |  |  |  |  |  |  |
| Beef | 4 | 46.72 | 22.50, 70.94 |  | 8 | 43.61 | 31.21, 56.01 |
| Lamb and goat meat | 8 | 13.39 | -4.27, 31.05 |  | 12 | 57.96 | -15.82, 131.74 |
| Pork | 4 | -6.51 | -50.46, 37.45 |  | 5 | -0.70 | -36.60, 35.19 |
| Chicken meat | 5 | 28.11 | -92.77, 148.99 |  | 6 | 19.36 | -80.82, 119.54 |
| EPA (cis-5,8,11,14,17-20:5)‡ | | |  |  |  |  |  |
| Beef | - | - | - |  | 5 | 55.74 | 28.96, 82.53 |
| Lamb and goat meat | 7 | -17.96 | -72.62, 36.71 |  | 10 | -25.88 | -68.30, 16.55 |
| Chicken meat | - | - | - |  | 4 | -24.33 | -94.40, 45.74 |
| DPA (cis-7,10,13,16,19-22:5) | | |  |  |  |  |  |
| Beef | - | - | - |  | 5 | 54.59 | 13.57, 95.62 |
| Lamb and goat meat | 7 | 19.55 | -13.60, 52.71 |  | 8 | 22.79 | -6.62, 52.2 |
| DHA (cis-4,7,10,13,16,19-22:6) | | |  |  |  |  |  |
| Beef | - | - | - |  | 5 | 39.39 | -15.78, 94.56 |
| Lamb and goat meat | 6 | 27.41 | -13.98, 68.80 |  | 10 | 19.94 | -6.40, 46.29 |
| Chicken meat | 5 | -19.41 | -139.29, 100.48 |  | 6 | -31.18 | -131.75, 69.39 |
| *n*, number of data points included in the comparison; MPD, mean percentage difference; OA, oleic acid; PUFA, polyunsaturated fatty acids; FA, fatty acids; ALA, α-linolenic acid; EPA, eicosapentaenoic acid; DPA, docosapentaenoic acid; DHA, docosahexaenoic acid;. *The summary results and product groups for which n≤3 were removed (for summary results see Table S9.), †Magnitude of difference between organic (ORG) and conventional (CONV) samples (value <0 indicate higher concentration in CONV, value >0 indicate higher concentration in ORG); ‡Outlying data pairs (where the MPD between ORG and CONV was over fifty times greater than the mean value including the outliers) were removed. | | | | | | | |

| **Table S10 cont.** Mean percentage differences (MPD) and confidence intervals (CI) calculated using the data included in standard meta-analyses and sensitivity meta-analysis 1 of composition parameters shown in Fig. 3 and 4 of the main paper (MPDs are also shown as symbols in Fig. 3 and 4). | | | | | | | |
| --- | --- | --- | --- | --- | --- | --- | --- |
|  | **Calculated based on data included in** | | | | | | |
| **Parameter/** | **standard meta-analysis** | | |  | **sensitivity meta-analysis 1** | | |
| **Animal group*** | ***n*** | **MPD**† | **95% CI** |  | ***n*** | **MPD**† | **95% CI** |
| VLC n-3 PUFA (EPA+DPA+DHA)§ | | |  |  |  |  |  |
| Beef | - | - | - |  | 5 | 52.69 | 18.88, 86.49 |
| Lamb and goat meat | - | - | - |  | 8 | 12.89 | -13.71, 39.48 |
| LA/ALA ratio§ |  |  |  |  |  |  |  |
| Beef | - | - | - |  | 7 | -42.06 | -69.11, -15 |
| Lamb and goat meat | - | - | - |  | 10 | -36.11 | -77.72, 5.49 |
| Pork | - | - | - |  | 5 | 9.41 | -13.84, 32.65 |
| n-6 FA |  |  |  |  |  |  |  |
| Beef | - | - | - |  | 7 | 1.46 | -17.58, 20.51 |
| Lamb and goat meat | 8 | 3.13 | -9.90, 16.15 |  | 11 | 1.56 | -7.94, 11.05 |
| Chicken meat | 4 | 48.33 | -2.01, 98.66 |  | 6 | 36.89 | 1.40, 72.37 |
| LA (cis-9,12-18:2) |  |  |  |  |  |  |  |
| Beef | 5 | 20.12 | -8.92, 49.17 |  | 8 | 6.51 | -15.31, 28.34 |
| Lamb and goat meat | 8 | 4.86 | -6.22, 15.93 |  | 10 | 13.19 | -7.43, 33.82 |
| Pork | 4 | 3.20 | -40.31, 46.70 |  | 5 | 8.91 | -26.60, 44.42 |
| Chicken meat | 5 | 8.56 | -78.80, 95.92 |  | 6 | 10.13 | -61.26, 81.53 |
| AA (cis-5,8,11,14-20:4)‡ | |  |  |  |  |  |  |
| Beef | - | - | - |  | 4 | 3.81 | -17.79, 25.41 |
| Lamb and goat meat | 6 | 0.17 | -19.37, 19.71 |  | 8 | -5.36 | -21.87, 11.16 |
| Chicken meat | - | - | - |  | 4 | 24.16 | -35.40, 83.72 |
| n-6/n-3 ratio |  |  |  |  |  |  |  |
| Beef | 5 | -55.73 | -106.62, -4.83 |  | 9 | -60.25 | -93.88, -26.61 |
| Lamb and goat meat | 6 | -1.47 | -9.16, 6.23 |  | 13 | -23.79 | -55.76, 8.19 |
| Chicken meat | - | - | - |  | 5 | 12.33 | -36.71, 61.37 |
| *n*, number of data points included in the comparison; MPD, mean percentage difference; VLC n-3 PUFA, very long chain n-3 PUFA; EPA, eicosapentaenoic acid; DPA, docosapentaenoic acid; DHA, docosahexaenoic acid; FA, fatty acids; LA, linoleic acid; AA, arachidonic acid. *The summary results and product groups for which n≤3 were removed (for summary results see Table S9.), †Magnitude of difference between organic (ORG) and conventional (CONV) samples (value <0 indicate higher concentration in CONV, value >0 indicate higher concentration in ORG); ‡Outlying data pairs (where the MPD between ORG and CONV was over fifty times greater than the mean value including the outliers) were removed; §Calculated based on published fatty acids composition data. | | | | | | | |

| **Table S11.** Meta-analysis results for additional composition parameters (protein, 20:0 (arachidic acid), 14:1, 16:1 (palmitoleic acid), 17:1 (heptadecenoic acid), CLA (cis-9-trans-11-18:2), PUFA/SFA ratio, n-3/n-6 ratio, EPA+DHA, atherogenicity index, thrombogenicity index, cholesterol, lipid oxidation (TBARS), Cu, Fe) for which significant differences were detected by the standard and sensitivity meta-analysis 1 protocols. | | | | | | | | | | | | | |
| --- | --- | --- | --- | --- | --- | --- | --- | --- | --- | --- | --- | --- | --- |
|  | **Standard meta-analysis** | | | | | | |  | **Sensitivity meta-analysis 1** | | | | |
| **Parameter** | ***n*** | **SMD** | **95% CI** | ***P**** | **Heterogeneity**† | **MPD**‡ | **95% CI** |  | ***n*** | **Ln ratio**§ | ***P**** | **MPD**‡ | **95% CI** |
| Protein | 17 | 0.19 | -0.17, 0.54 | 0.307 | Yes (78%) | 1.02 | -0.51, 2.55 |  | 23 | 4.62 | 0.059 | 1.07 | -0.20, 2.34 |
| 20:0 (arachidic acid) | 9 | 0.33 | -0.15, 0.81 | 0.177 | Yes (81%) | 53.61 | -39.93, 147.16 |  | 12 | 4.91 | 0.020 | 66.98 | -14.18, 148.14 |
| 14:1 | 4 | -0.02 | -0.43, 0.39 | 0.909 | No (0%) | -1.85 | -15.46, 11.77 |  | 6 | 4.42 | 0.141 | -27.93 | -76.62, 20.77 |
| 16:1 (palmitoleic acid) | 18 | -0.10 | -0.36, 0.16 | 0.443 | Yes (53%) | -9.10 | -30.33, 12.13 |  | 23 | 4.55 | 0.182 | -10.04 | -27.65, 7.57 |
| 17:1 (heptadecenoic acid) | 8 | 0.29 | -0.30, 0.89 | 0.331 | Yes (82%) | 16.18 | -6.24, 38.59 |  | 11 | 4.73 | 0.049 | 15.23 | -1.27, 31.74 |
| CLA (cis-9-trans-11-18:2) | 5 | -0.66 | -1.19, -0.13 | 0.015 | Yes (68%) | -22.17 | -44.50, 0.16 |  | 11 | 4.59 | 0.411 | -2.19 | -21.96, 17.59 |
| PUFA/SFA ratio | 4 | 2.75 | -2.05, 7.55 | 0.261 | Yes (100%) | 50.44 | -33.29, 134.16 |  | 10 | 4.85 | 0.015 | 36.28 | -1.26, 73.82 |
| n-3/n-6 ratio | - | - | - | - | - | - | - |  | 31 | 4.80 | 0.008 | 29.21 | 5.86, 52.56 |
| EPA+DHA¶ | - | - | - | - | - | - | - |  | 22 | 4.70 | 0.262 | 26.34 | -55.90, 108.59 |
| Atherogenicity index | 4 | 0.47 | -0.17, 1.11 | 0.148 | Yes (79%) | 6.64 | -0.66, 13.94 |  | 5 | 4.67 | 0.062 | 7.02 | 1.32, 12.72 |
| Atherogenicity index¶ | - | - | - | - | - | - | - |  | 13 | 4.58 | 0.221 | -2.53 | -8.37, 3.3 |
| Thrombogenicity index | 4 | -0.35 | -0.64, -0.06 | 0.018 | No (0%) | -4.40 | -6.73, -2.08 |  | 5 | 4.57 | 0.028 | -3.99 | -5.97, -2.02 |
| Thrombogenicity index¶ | - | - | - | - | - | - | - |  | 15 | 4.54 | 0.025 | -7.77 | -15.1, -0.44 |
| Cholesterol | - | - | - | - | - | - | - |  | 5 | 4.58 | 0.189 | -3.01 | -10.60, 4.59 |
| Lipid oxidation (TBARS)\|\| | 8 | 0.19 | -0.18, 0.56 | 0.310 | Yes (52%) | 23.03 | -9.35, 55.42 |  | 11 | 4.75 | 0.050 | 19.53 | -4.09, 43.15 |
| Se | - | - | - | - | - | - | - |  | 3 | 4.53 | 0.256 | -8.12 | -27.34, 11.1 |
| Cu | 3 | -4.77 | -8.92, -0.63 | 0.024 | Yes (98%) | -25.96 | -42.61, -9.30 |  | 4 | 4.36 | 0.064 | -27.80 | -40.12, -15.48 |
| Fe | 4 | 1.00 | -0.65, 2.66 | 0.236 | Yes (96%) | 13.79 | 2.14, 25.43 |  | 5 | 4.77 | 0.068 | 18.86 | 5.44, 32.27 |
| *n*, number of data points included in the comparison; MPD, mean percentage difference; SMD, standardised mean difference of random-effect model; CLA, conjugated linoleic acids; PUFA, polyunsaturated fatty acids; SFA, saturated fatty acids EPA, eicosapentaenoic acid; DHA, docosahexaenoic acid. **P* value <0.05 indicates significance of the difference in composition between organic and conventional meat; †Heterogeneity and the I^2^ Statistic; ‡Magnitude of difference between organic (ORG) and conventional (CONV) samples (value <0 indicate higher concentration in CONV, value >0 indicate higher concentration in ORG); §Ln ratio = Ln(ORG/CONV × 100%); \|\|Outlying data pairs (where the MPD between ORG and CONV was over fifty times greater than the mean value including the outliers) were removed; ¶Calculated based on published fatty acids composition data. | | | | | | | | | | | | | |


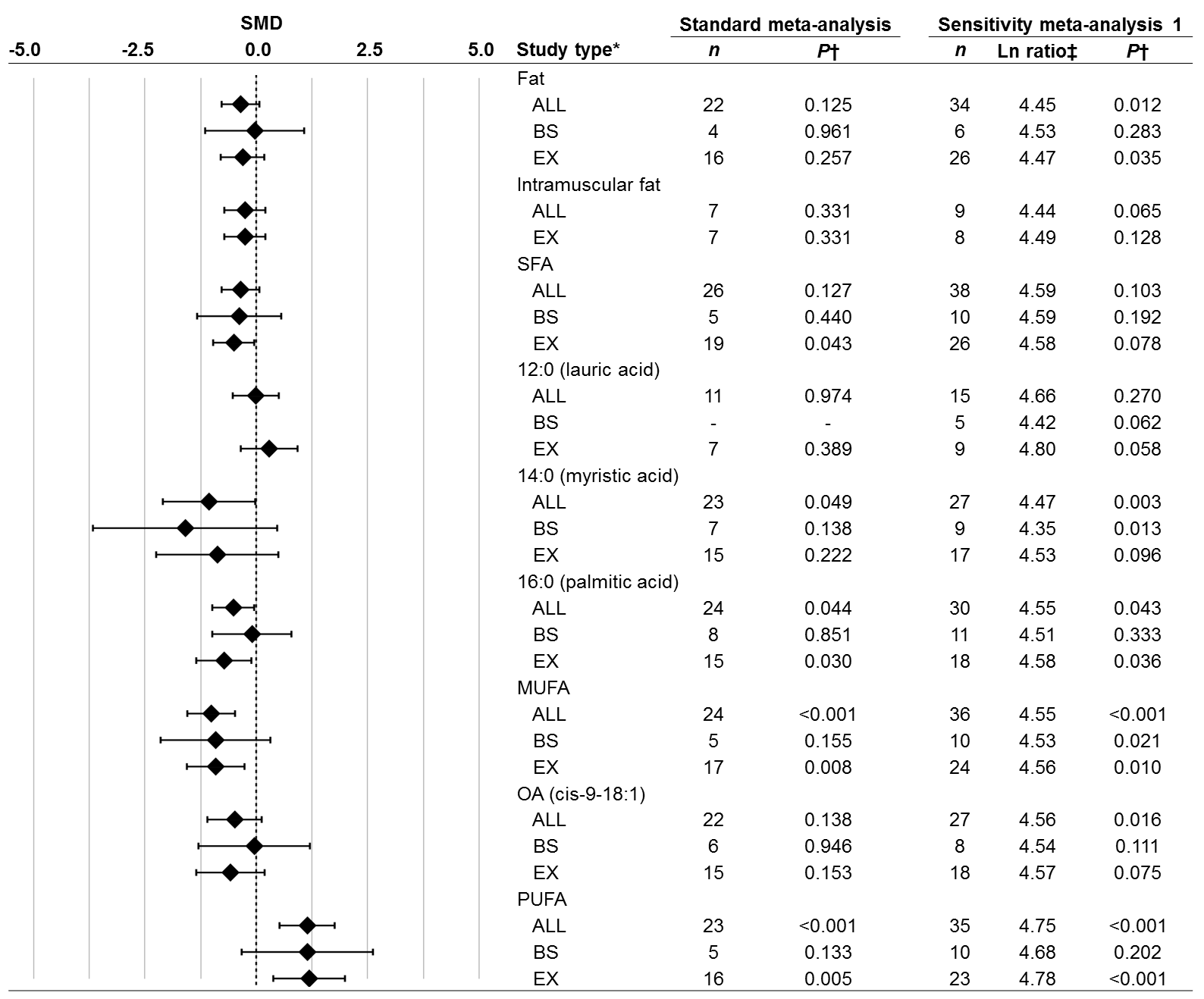


**Figure S3.** Results of the standard meta-analyses and sensitivity meta-analysis 1 for different study types for fat composition in meat. SMD, standardised mean difference with 95% confidence intervals represented by horizontal bars; *n*, number of data points included in meta-analyses; CF, comparison of farms, BS, basket study, EX, controlled experiment; SFA, saturated fatty acids; MUFA, monounsaturated fatty acids; OA, oleic acid; PUFA, polyunsaturated fatty acids. *for parameters for which *n* ≤ 3 for specific study type, results obtained in the weighted meta-analyses are not shown, †*P* value <0.05 indicates a significant difference between ORG and CONV, ‡Ln ratio = Ln(ORG/CONV × 100%).


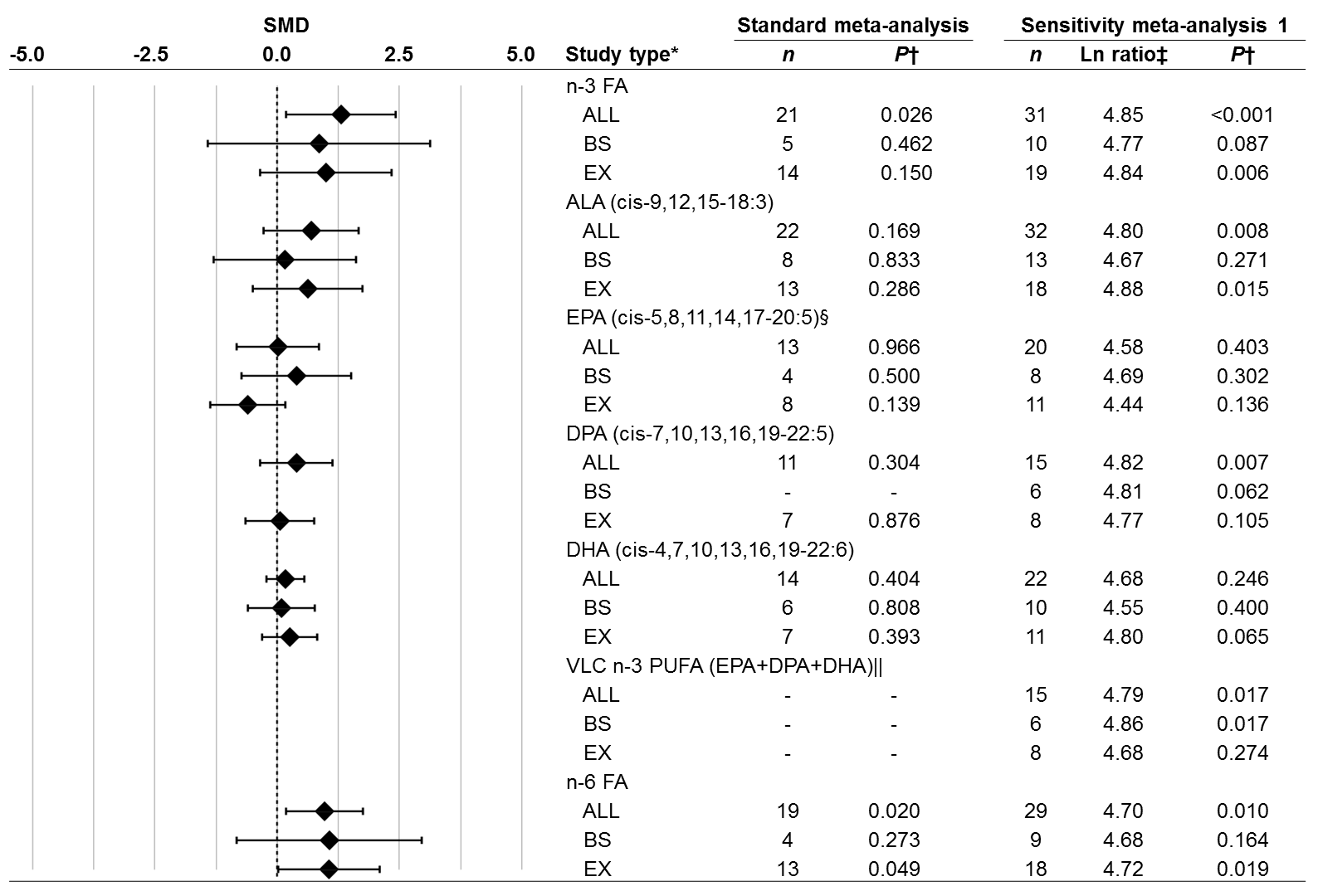


**Figure S4.** Results of the standard meta-analyses and sensitivity meta-analysis 1 for different study types for fat composition in meat. SMD, standardised mean difference with 95% confidence intervals represented by horizontal bars; n, number of data points included in meta-analyses; CF, comparison of farms, BS, basket study, EX, controlled experiment; FA, fatty acids; ALA, α-linolenic acid; EPA, eicosapentaenoic acid; DPA, docosapentaenoic acid; DHA, docosahexaenoic acid; VLC n-3 PUFA, very long chain n-3 polyunsaturated fatty acids. *For parameters for which *n* ≤ 3 for specific study type, results obtained in the weighted meta-analyses are not shown, †*P* value <0.05 indicates a significant difference between ORG and CONV, ‡Ln ratio = Ln(ORG/CONV × 100%), §outlying data points (where the MPD between ORG and CONV was more than fifty times greater than the mean value including the outliers) were removed, ||calculated based on published fatty acids composition data.


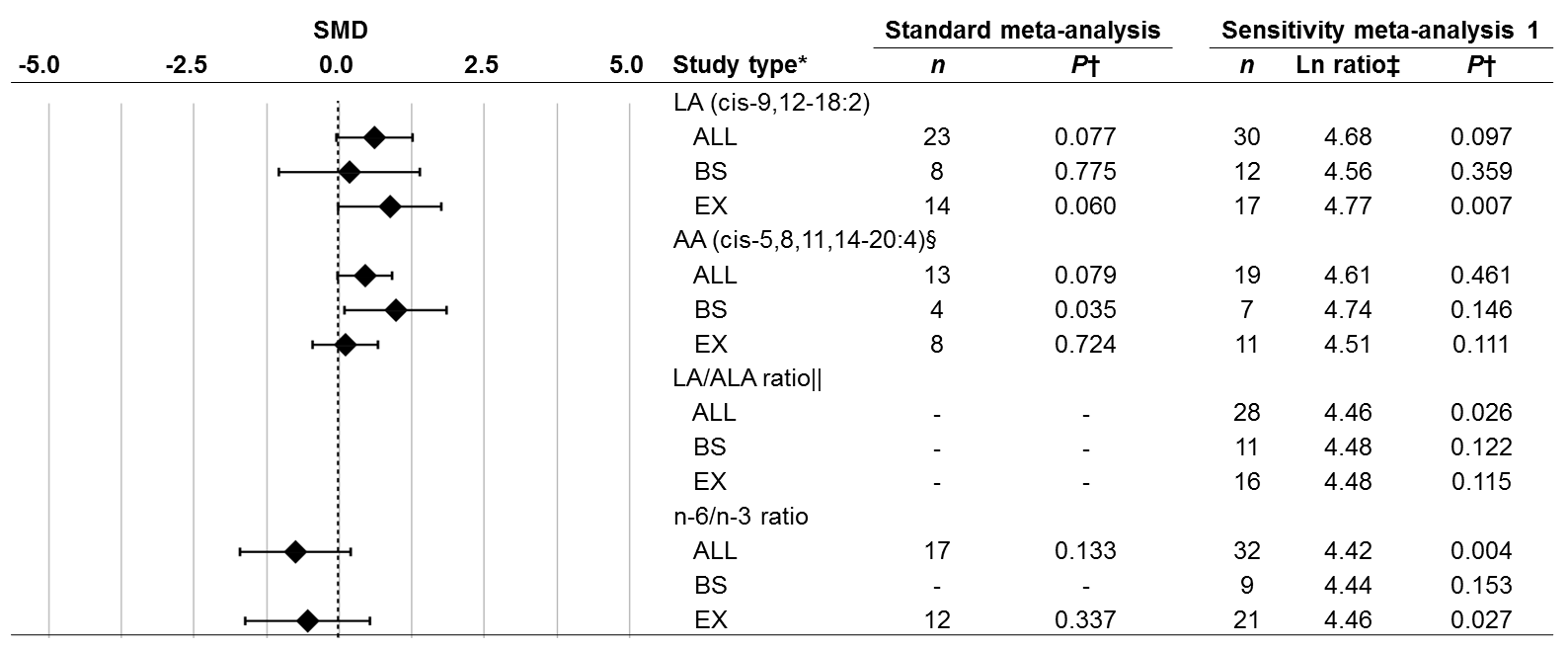


**Figure S5.** Results of the standard meta-analyses and sensitivity meta-analysis 1 for different study types for fat composition in meat. SMD, standardised mean difference with 95% confidence intervals represented by horizontal bars; n, number of data points included in meta-analyses; CF, comparison of farms, BS, basket study, EX, controlled experiment; LA, linoleic acid; AA, arachidonic acid; ALA, α-linolenic acid. *For parameters for which *n* ≤ 3 for specific study type, results obtained in the weighted meta-analyses are not shown, †*P* value <0.05 indicates a significant difference between ORG and CONV, ‡Ln ratio = Ln(ORG/CONV × 100%), §outlying data points (where the MPD between ORG and CONV was more than fifty times greater than the mean value including the outliers) were removed, ||calculated based on published fatty acids composition data.


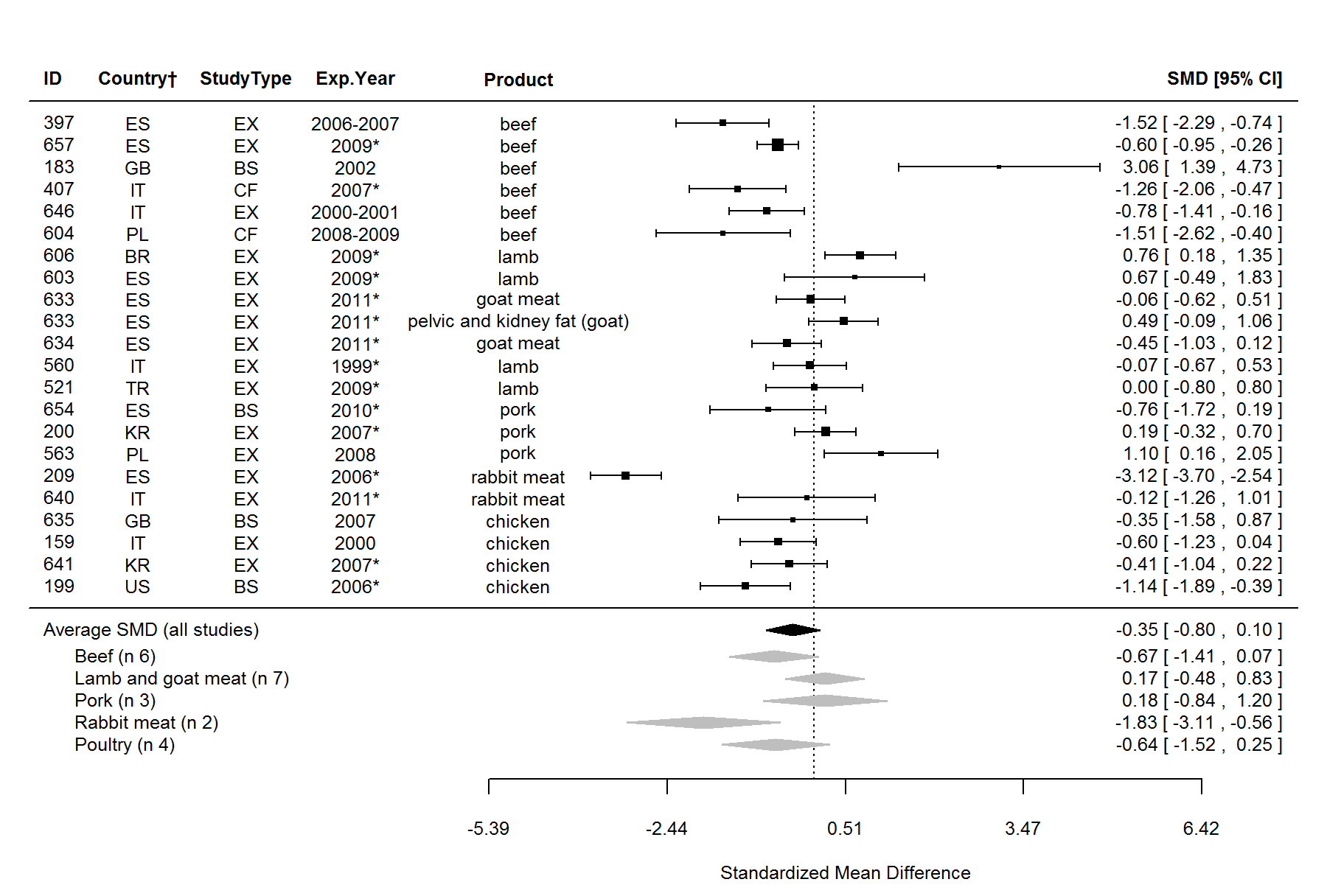


**Figure S6.** Forest plot showing the results of the comparison of fat content between organic and conventional meat using standardised mean differences (SMDs) with 95% confidence intervals, for studies included in standard meta-analysis. The estimated average SMD for all studies and SMDs for different animal groups are indicated at the bottom of the figure. Sign of the SMD indicates if the analysed parameter is higher (+) or lower (-) in organic foods. ID, Paper unique identification number (see supplementary Table S2 for references); CF, comparison of farms, BS, basket study, EX, controlled experiment. *No information about the experimental year (estimated as publication year - 2), †Country codes according ISO 3166-2 (see [*http://www.iso.org/iso/home/standards/country_codes.htm*](http://www.iso.org/iso/home/standards/country_codes.htm)).


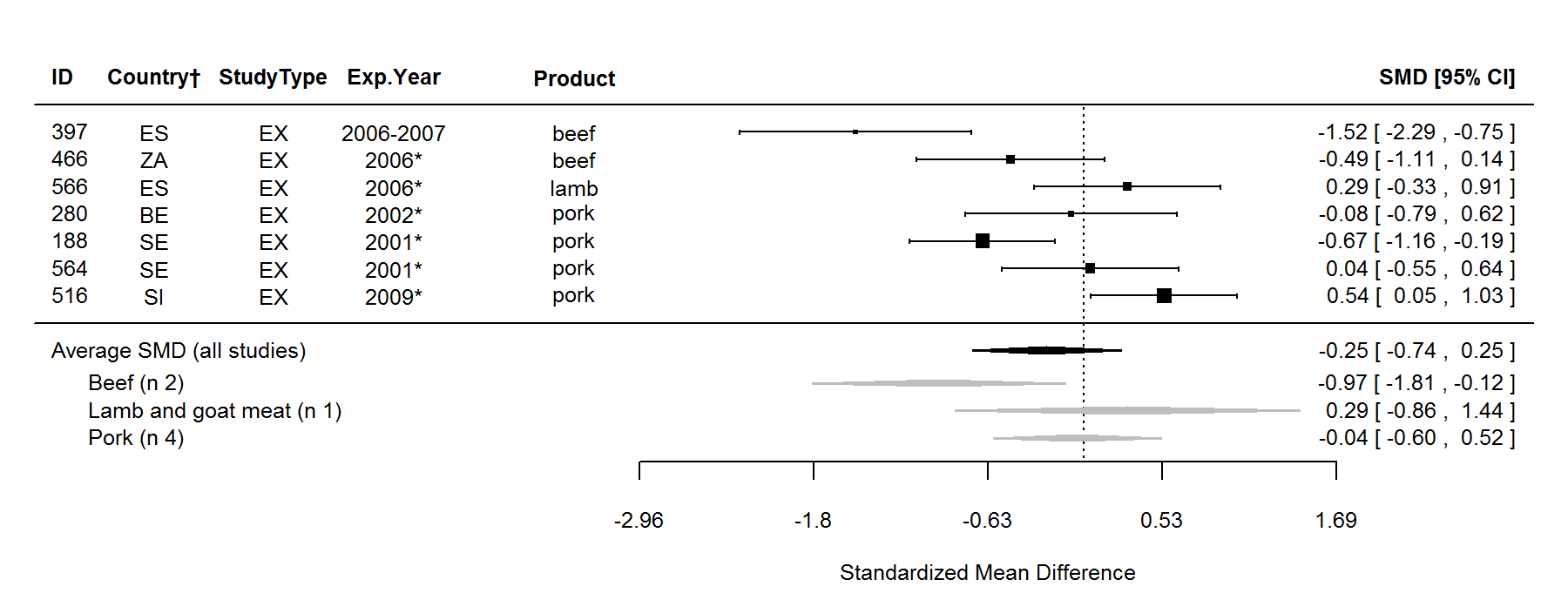


**Figure S7.** Forest plot showing the results of the comparison of intramuscular fat content between organic and conventional meat using standardised mean differences (SMDs) with 95% confidence intervals, for studies included in standard meta-analysis. The estimated average SMD for all studies and SMDs for different animal groups are indicated at the bottom of the figure. Sign of the SMD indicates if the analysed parameter is higher (+) or lower (-) in organic foods. ID, Paper unique identification number (see supplementary Table S2 for references); CF, comparison of farms, BS, basket study, EX, controlled experiment. *No information about the experimental year (estimated as publication year - 2), †Country codes according ISO 3166-2 (see [*http://www.iso.org/iso/home/standards/country_codes.htm*](http://www.iso.org/iso/home/standards/country_codes.htm)).


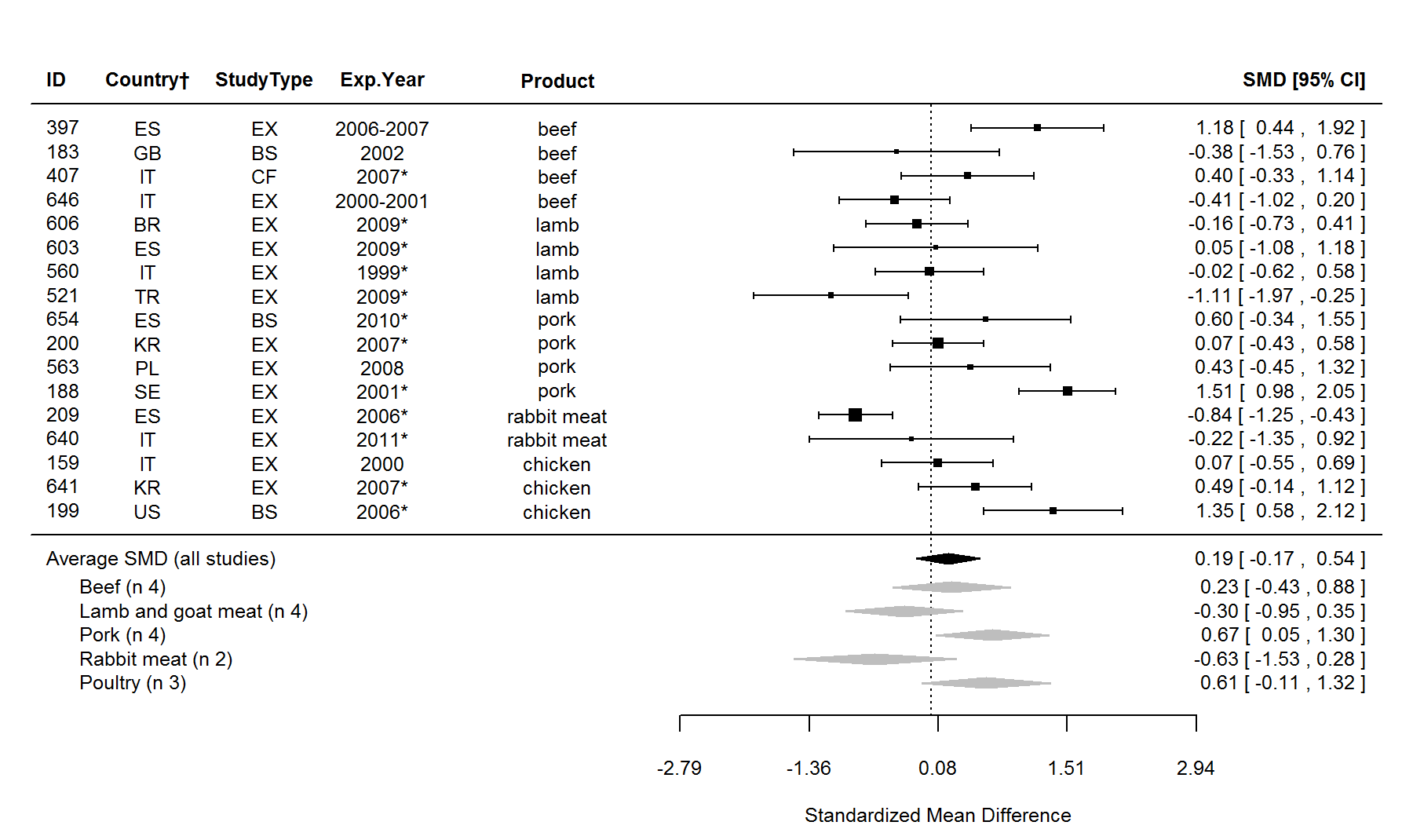


**Figure S8.** Forest plot showing the results of the comparison of protein content between organic and conventional meat using standardised mean differences (SMDs) with 95% confidence intervals, for studies included in standard meta-analysis. The estimated average SMD for all studies and SMDs for different animal groups are indicated at the bottom of the figure. Sign of the SMD indicates if the analysed parameter is higher (+) or lower (-) in organic foods. ID, Paper unique identification number (see supplementary Table S2 for references); CF, comparison of farms, BS, basket study, EX, controlled experiment. *No information about the experimental year (estimated as publication year - 2), †Country codes according ISO 3166-2 (see [*http://www.iso.org/iso/home/standards/country_codes.htm*](http://www.iso.org/iso/home/standards/country_codes.htm)).


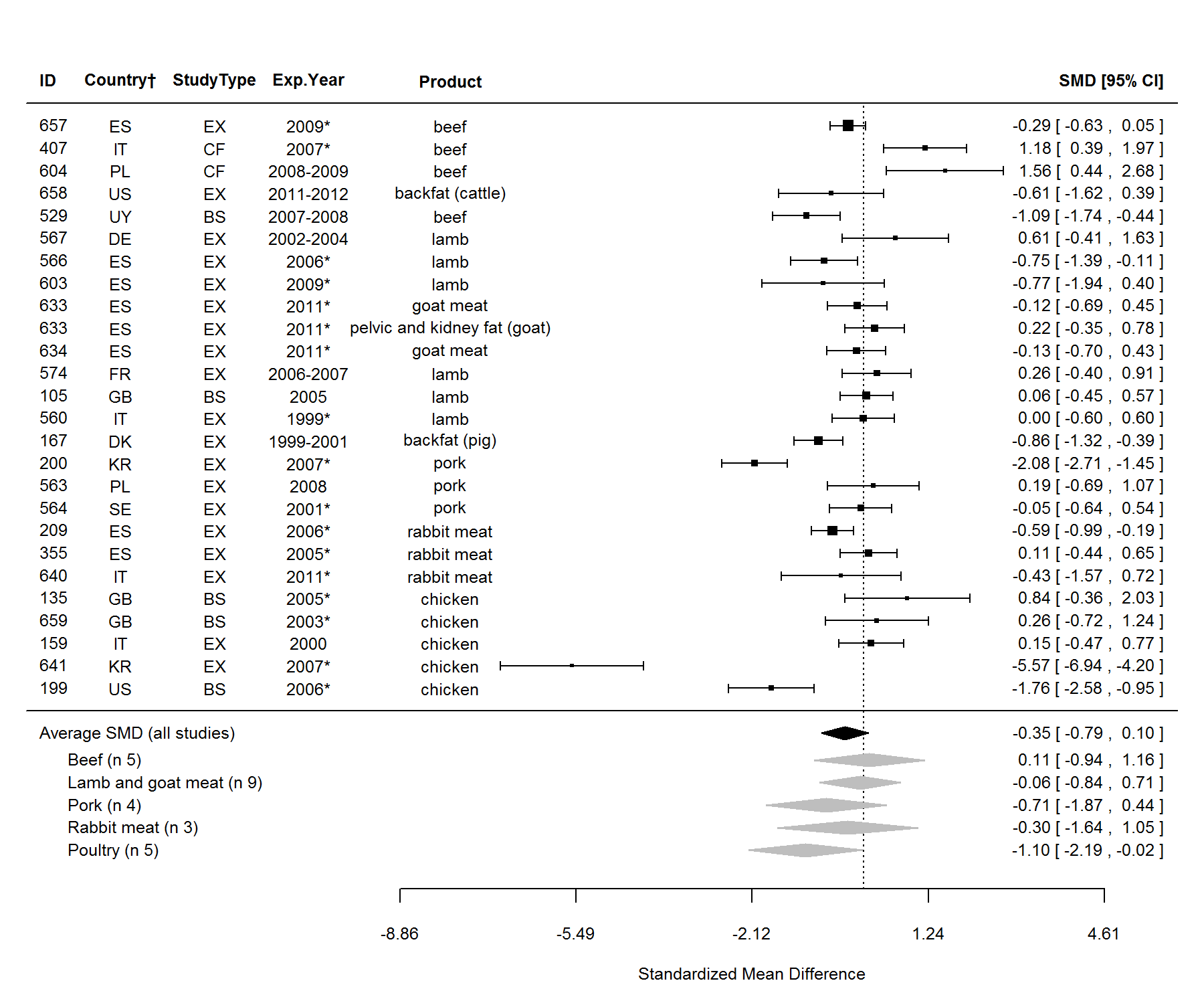


**Figure S9.** Forest plot showing the results of the comparison of saturated fatty acids (SFA) content between organic and conventional meat using standardised mean differences (SMDs) with 95% confidence intervals, for studies included in standard meta-analysis. The estimated average SMD for all studies and SMDs for different animal groups are indicated at the bottom of the figure. Sign of the SMD indicates if the analysed parameter is higher (+) or lower (-) in organic foods. ID, Paper unique identification number (see supplementary Table S2 for references); CF, comparison of farms, BS, basket study, EX, controlled experiment. *No information about the experimental year (estimated as publication year - 2), †Country codes according ISO 3166-2 (see [*http://www.iso.org/iso/home/standards/country_codes.htm*](http://www.iso.org/iso/home/standards/country_codes.htm)).


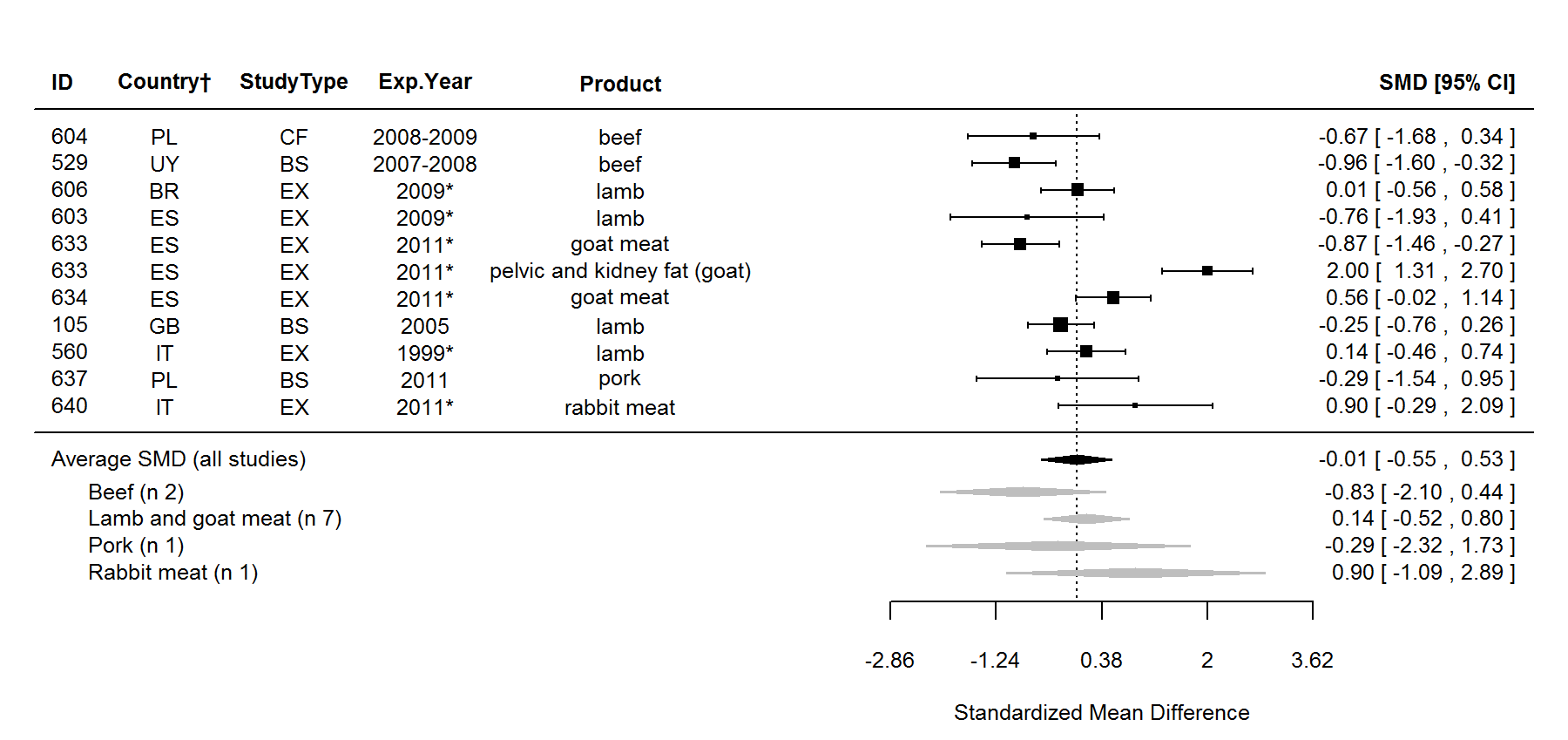


**Figure S10.** Forest plot showing the results of the comparison of 12:0 (lauric acid) content between organic and conventional meat using standardised mean differences (SMDs) with 95% confidence intervals, for studies included in standard meta-analysis. The estimated average SMD for all studies and SMDs for different animal groups are indicated at the bottom of the figure. Sign of the SMD indicates if the analysed parameter is higher (+) or lower (-) in organic foods. ID, Paper unique identification number (see supplementary Table S2 for references); CF, comparison of farms, BS, basket study, EX, controlled experiment. *No information about the experimental year (estimated as publication year - 2), †Country codes according ISO 3166-2 (see [*http://www.iso.org/iso/home/standards/country_codes.htm*](http://www.iso.org/iso/home/standards/country_codes.htm)).


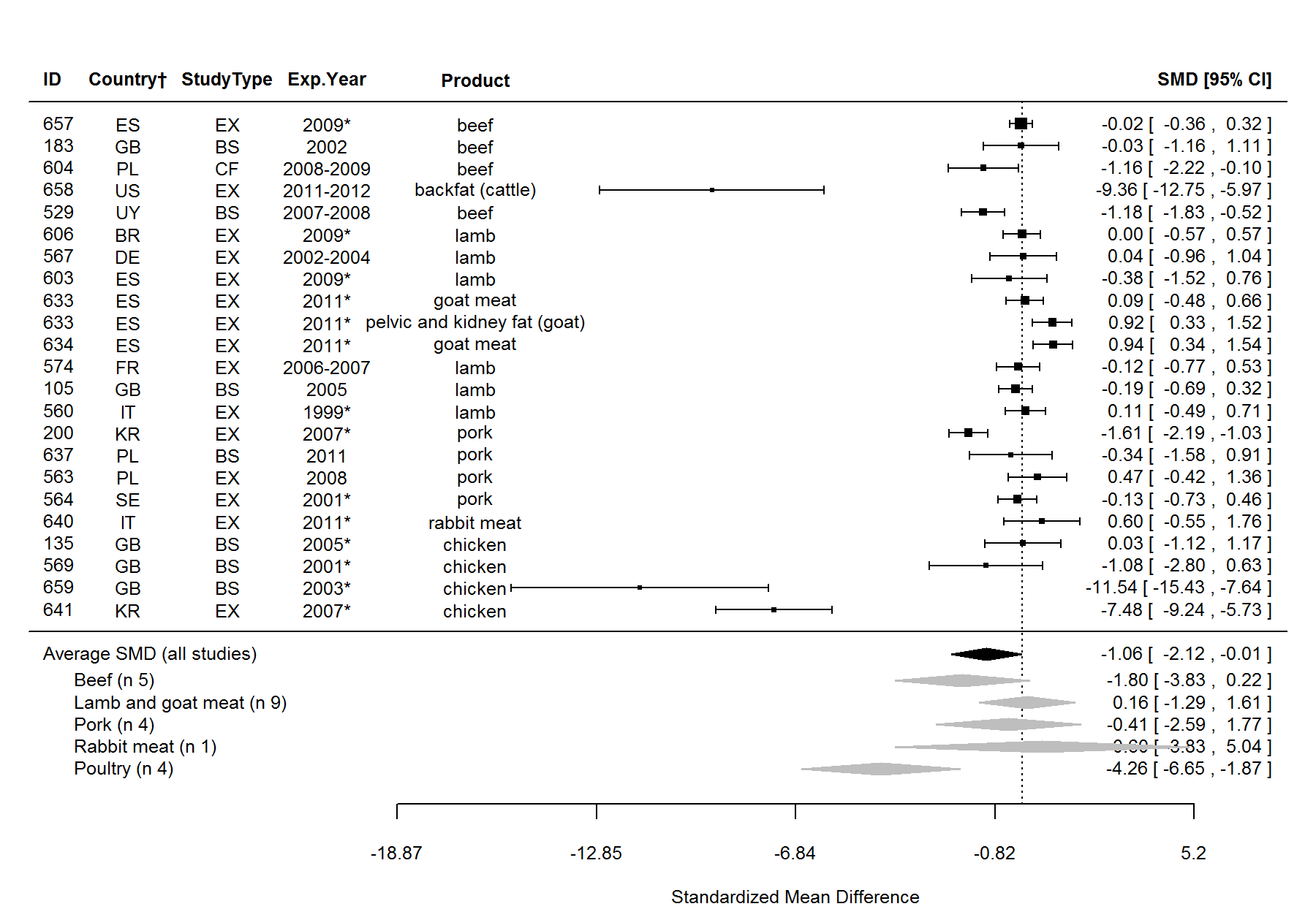


**Figure S11.** Forest plot showing the results of the comparison of 14:0 (myristic acid) content between organic and conventional meat using standardised mean differences (SMDs) with 95% confidence intervals, for studies included in standard meta-analysis. The estimated average SMD for all studies and SMDs for different animal groups are indicated at the bottom of the figure. Sign of the SMD indicates if the analysed parameter is higher (+) or lower (-) in organic foods. ID, Paper unique identification number (see supplementary Table S2 for references); CF, comparison of farms, BS, basket study, EX, controlled experiment. *No information about the experimental year (estimated as publication year - 2), †Country codes according ISO 3166-2 (see [*http://www.iso.org/iso/home/standards/country_codes.htm*](http://www.iso.org/iso/home/standards/country_codes.htm)).


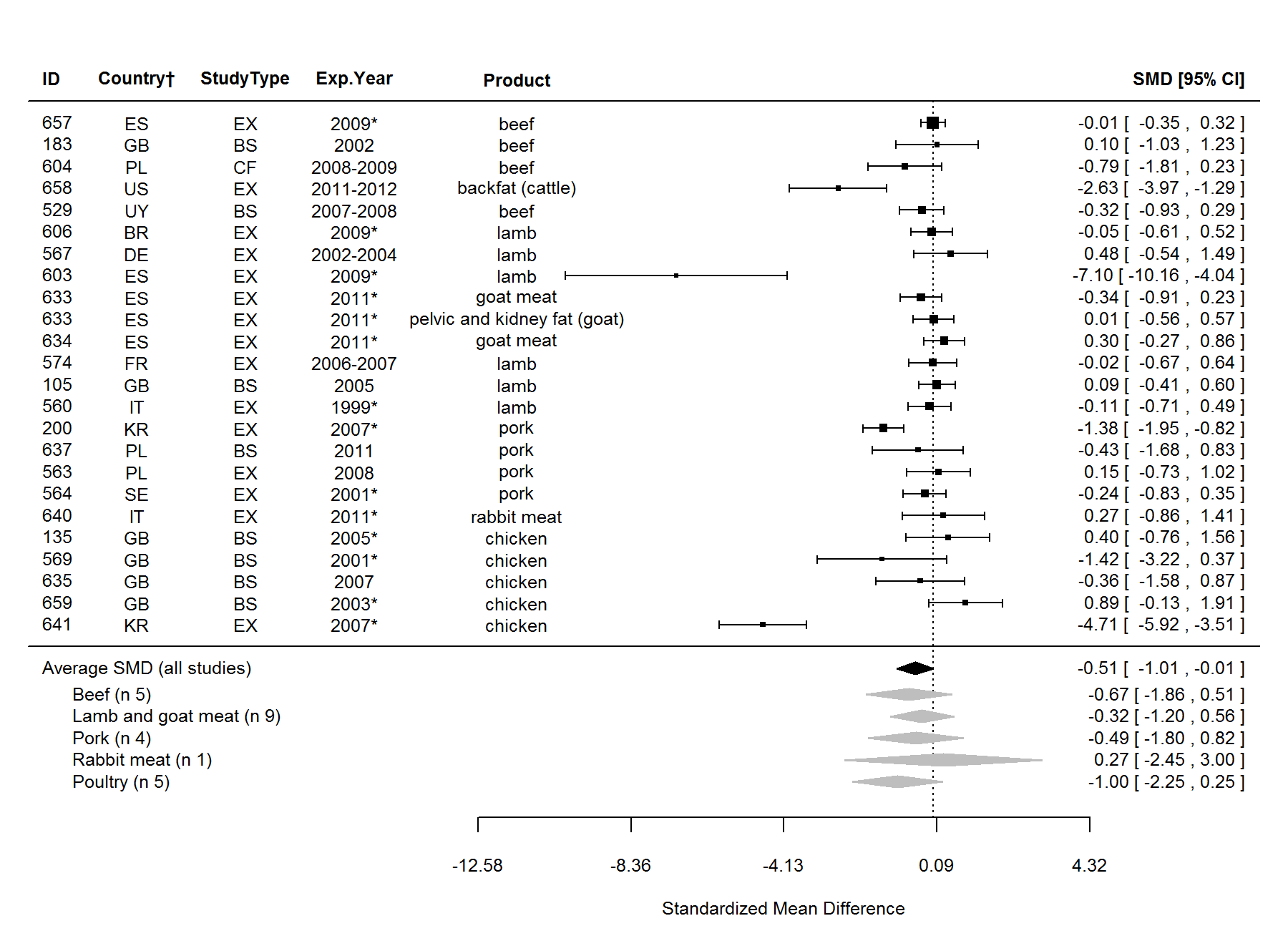


**Figure S12.** Forest plot showing the results of the comparison of 16:0 (palmitic acid) content between organic and conventional meat using standardised mean differences (SMDs) with 95% confidence intervals, for studies included in standard meta-analysis. The estimated average SMD for all studies and SMDs for different animal groups are indicated at the bottom of the figure. Sign of the SMD indicates if the analysed parameter is higher (+) or lower (-) in organic foods. ID, Paper unique identification number (see supplementary Table S2 for references); CF, comparison of farms, BS, basket study, EX, controlled experiment. *No information about the experimental year (estimated as publication year - 2), †Country codes according ISO 3166-2 (see [*http://www.iso.org/iso/home/standards/country_codes.htm*](http://www.iso.org/iso/home/standards/country_codes.htm)).


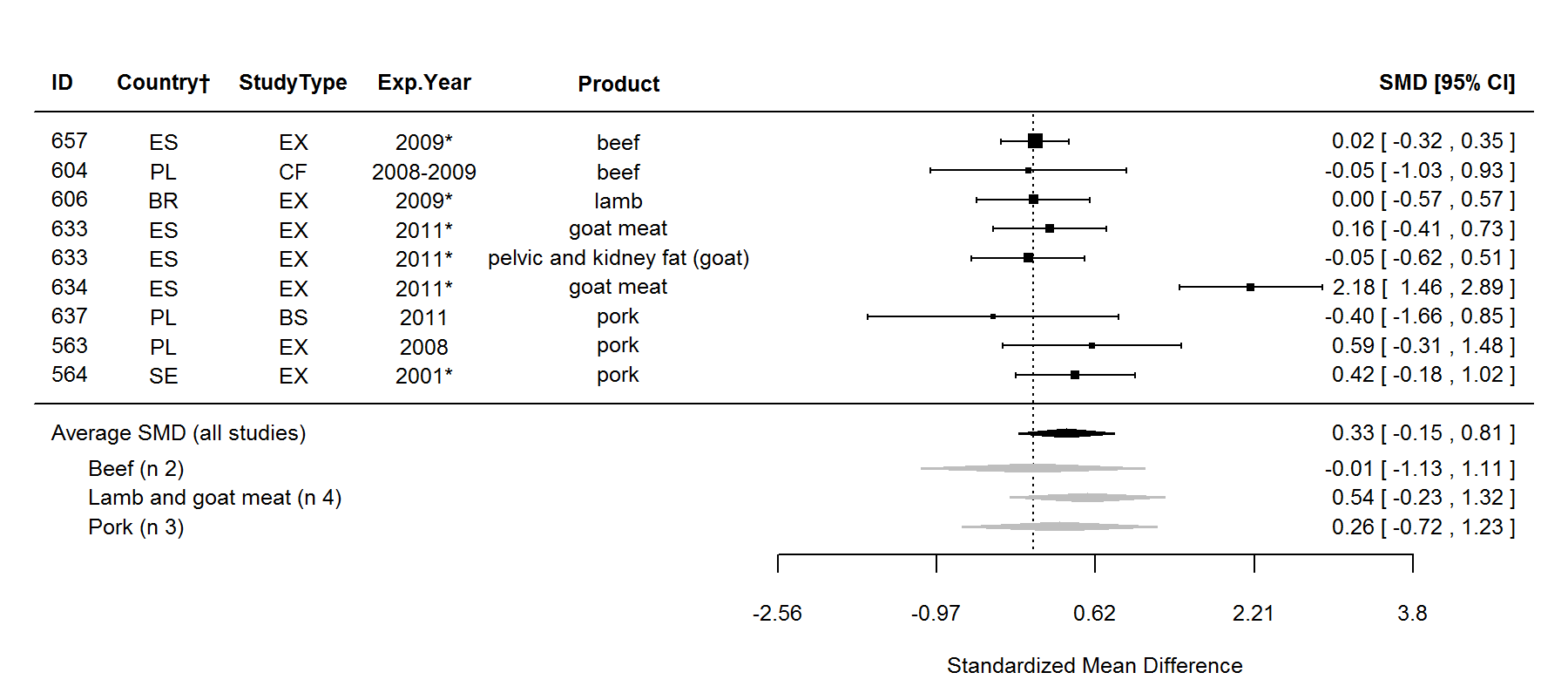


**Figure S13.** Forest plot showing the results of the comparison of 20:0 (arachidic acid) content between organic and conventional meat using standardised mean differences (SMDs) with 95% confidence intervals, for studies included in standard meta-analysis. The estimated average SMD for all studies and SMDs for different animal groups are indicated at the bottom of the figure. Sign of the SMD indicates if the analysed parameter is higher (+) or lower (-) in organic foods. ID, Paper unique identification number (see supplementary Table S2 for references); CF, comparison of farms, BS, basket study, EX, controlled experiment. *No information about the experimental year (estimated as publication year - 2), †Country codes according ISO 3166-2 (see [*http://www.iso.org/iso/home/standards/country_codes.htm*](http://www.iso.org/iso/home/standards/country_codes.htm)).


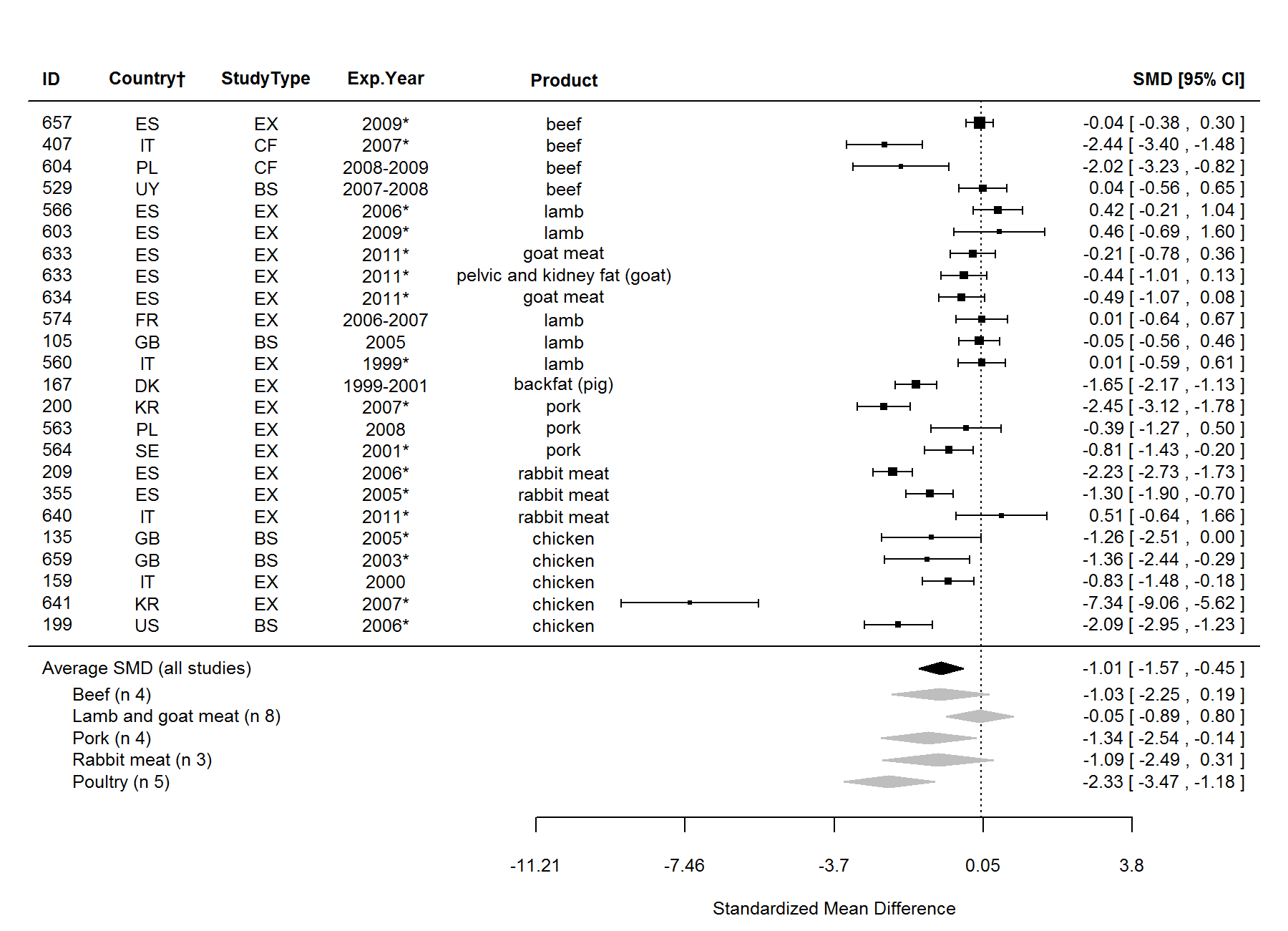


**Figure S14.** Forest plot showing the results of the comparison of monounsaturated fatty acids (MUFA) content between organic and conventional meat using standardised mean differences (SMDs) with 95% confidence intervals, for studies included in standard meta-analysis. The estimated average SMD for all studies and SMDs for different animal groups are indicated at the bottom of the figure. Sign of the SMD indicates if the analysed parameter is higher (+) or lower (-) in organic foods. ID, Paper unique identification number (see supplementary Table S2 for references); CF, comparison of farms, BS, basket study, EX, controlled experiment. *No information about the experimental year (estimated as publication year - 2), †Country codes according ISO 3166-2 (see [*http://www.iso.org/iso/home/standards/country_codes.htm*](http://www.iso.org/iso/home/standards/country_codes.htm)).


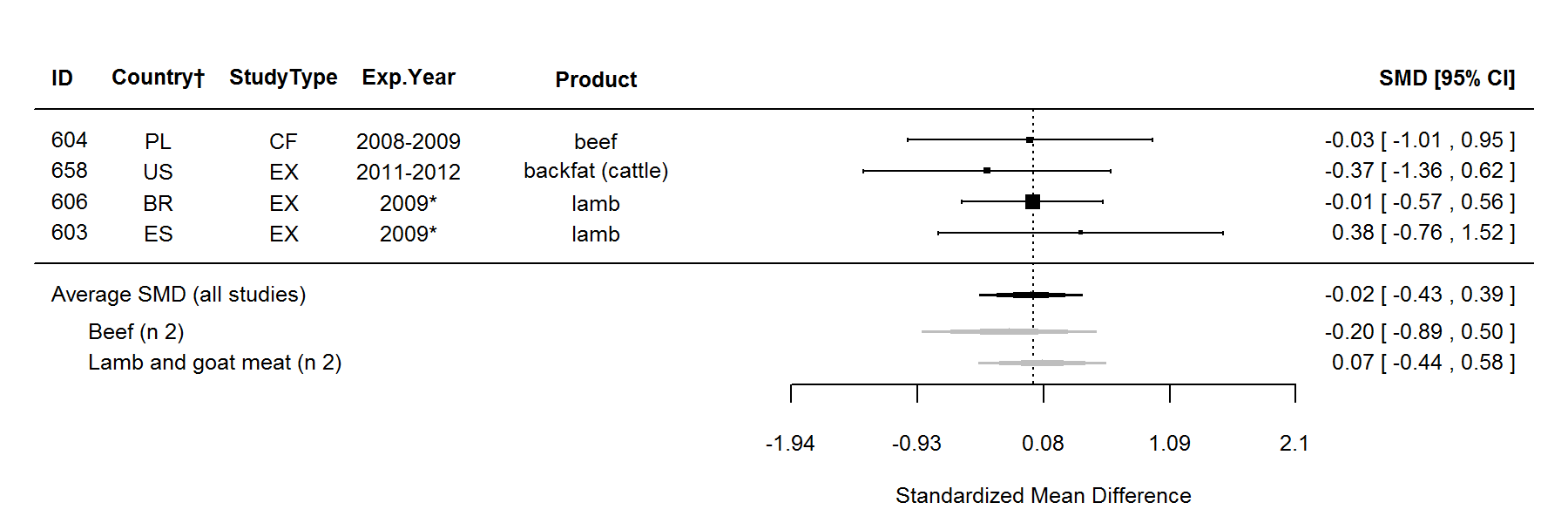


**Figure S15.** Forest plot showing the results of the comparison of 14:1 fatty acid content between organic and conventional meat using standardised mean differences (SMDs) with 95% confidence intervals, for studies included in standard meta-analysis. The estimated average SMD for all studies and SMDs for different animal groups are indicated at the bottom of the figure. Sign of the SMD indicates if the analysed parameter is higher (+) or lower (-) in organic foods. ID, Paper unique identification number (see supplementary Table S2 for references); CF, comparison of farms, BS, basket study, EX, controlled experiment. *No information about the experimental year (estimated as publication year - 2), †Country codes according ISO 3166-2 (see [*http://www.iso.org/iso/home/standards/country_codes.htm*](http://www.iso.org/iso/home/standards/country_codes.htm)).


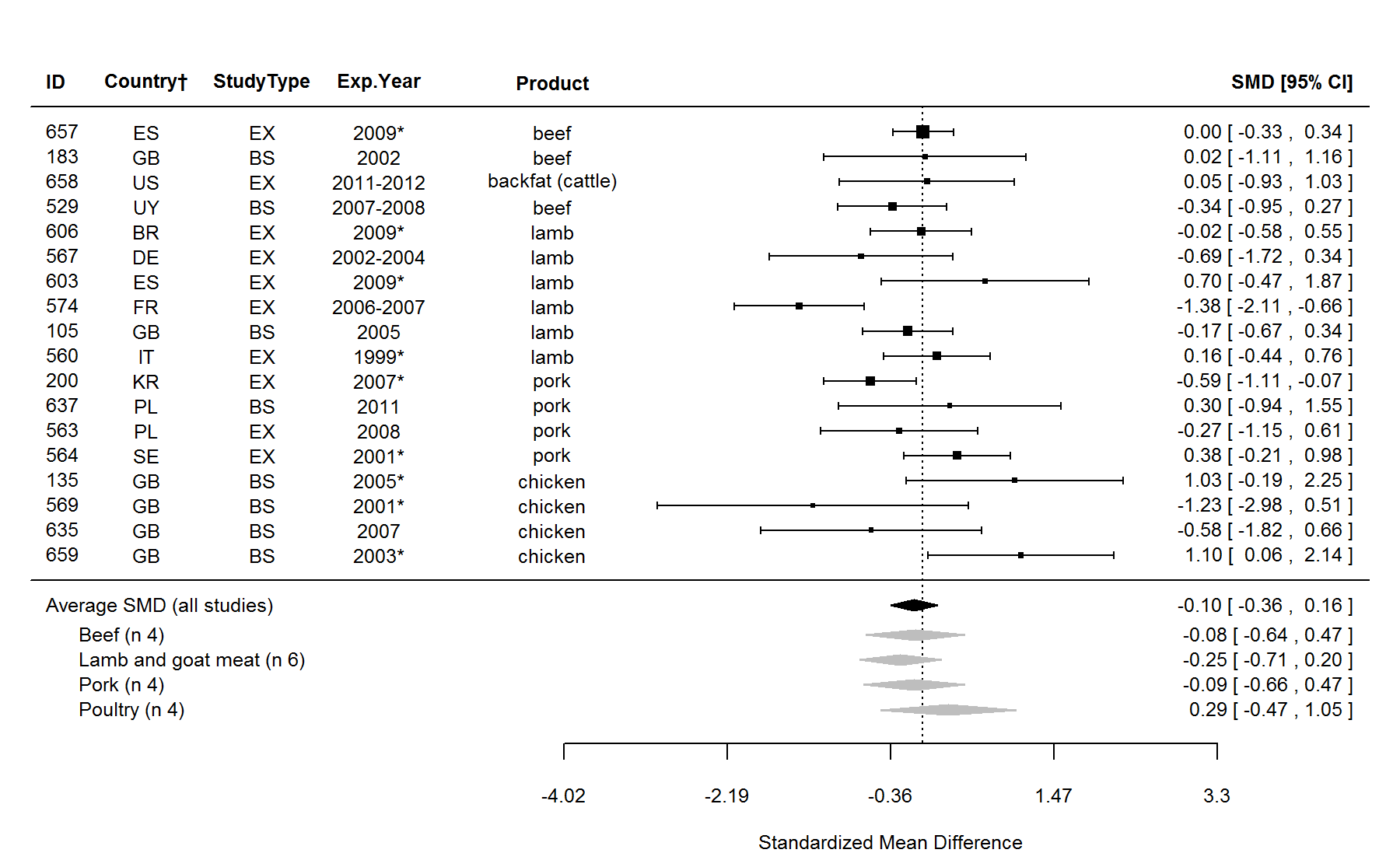


**Figure S16.** Forest plot showing the results of the comparison of 16:1 (palmitoleic acid) content between organic and conventional meat using standardised mean differences (SMDs) with 95% confidence intervals, for studies included in standard meta-analysis. The estimated average SMD for all studies and SMDs for different animal groups are indicated at the bottom of the figure. Sign of the SMD indicates if the analysed parameter is higher (+) or lower (-) in organic foods. ID, Paper unique identification number (see supplementary Table S2 for references); CF, comparison of farms, BS, basket study, EX, controlled experiment. *No information about the experimental year (estimated as publication year - 2), †Country codes according ISO 3166-2 (see [*http://www.iso.org/iso/home/standards/country_codes.htm*](http://www.iso.org/iso/home/standards/country_codes.htm)).


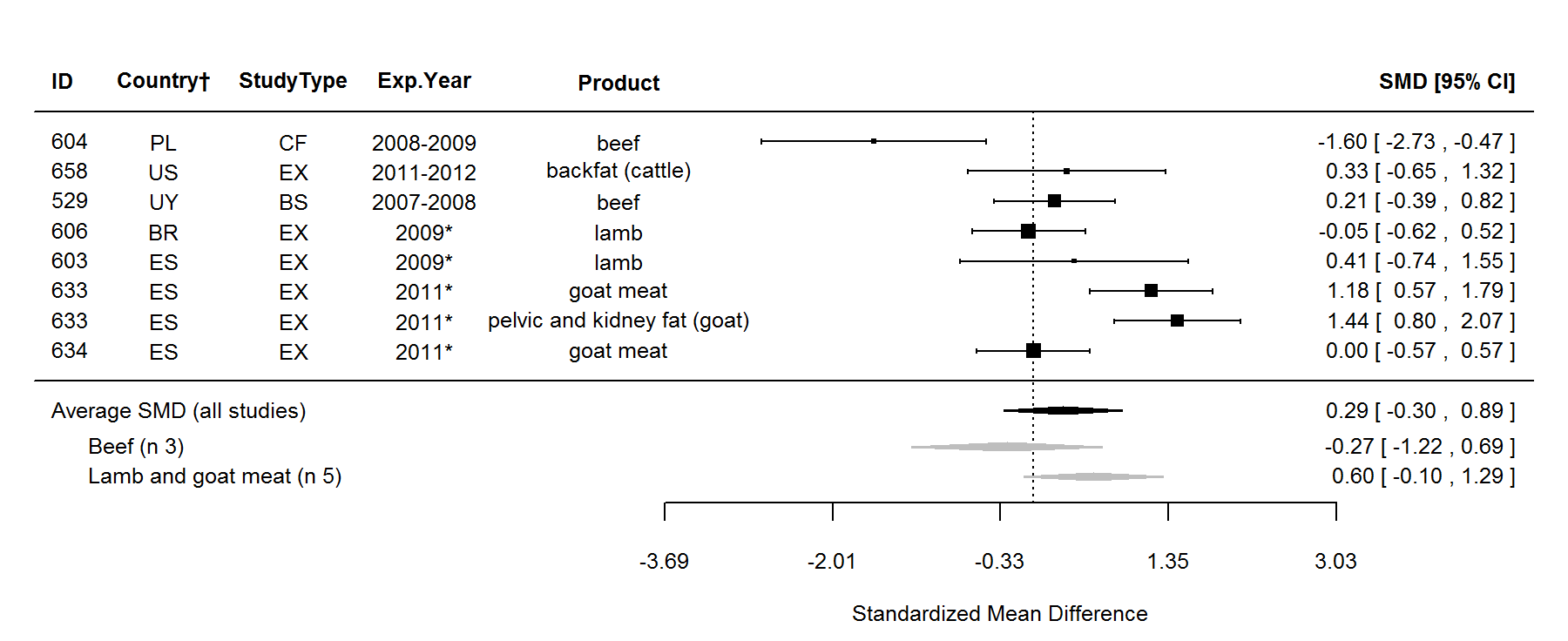


**Figure S17.** Forest plot showing the results of the comparison of 17:1 (heptadecenoic acid) content between organic and conventional meat using standardised mean differences (SMDs) with 95% confidence intervals, for studies included in standard meta-analysis. The estimated average SMD for all studies and SMDs for different animal groups are indicated at the bottom of the figure. Sign of the SMD indicates if the analysed parameter is higher (+) or lower (-) in organic foods. ID, Paper unique identification number (see supplementary Table S2 for references); CF, comparison of farms, BS, basket study, EX, controlled experiment. *No information about the experimental year (estimated as publication year - 2), †Country codes according ISO 3166-2 (see [*http://www.iso.org/iso/home/standards/country_codes.htm*](http://www.iso.org/iso/home/standards/country_codes.htm)).


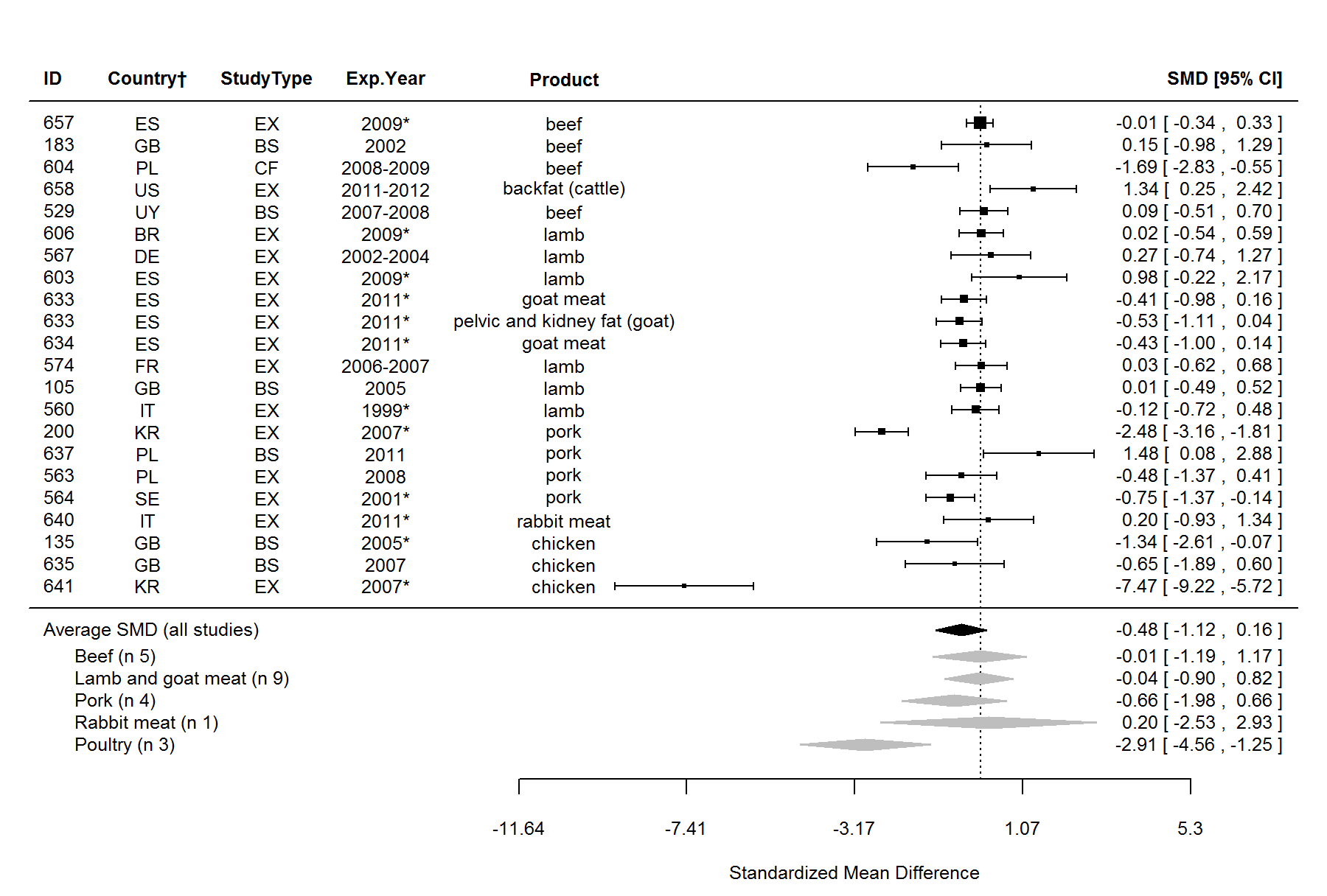


**Figure S18.** Forest plot showing the results of the comparison of oleic acid (OA, cis-9-18:1) content between organic and conventional meat using standardised mean differences (SMDs) with 95% confidence intervals, for studies included in standard meta-analysis. The estimated average SMD for all studies and SMDs for different animal groups are indicated at the bottom of the figure. Sign of the SMD indicates if the analysed parameter is higher (+) or lower (-) in organic foods. ID, Paper unique identification number (see supplementary Table S2 for references); CF, comparison of farms, BS, basket study, EX, controlled experiment. *No information about the experimental year (estimated as publication year - 2), †Country codes according ISO 3166-2 (see [*http://www.iso.org/iso/home/standards/country_codes.htm*](http://www.iso.org/iso/home/standards/country_codes.htm)).


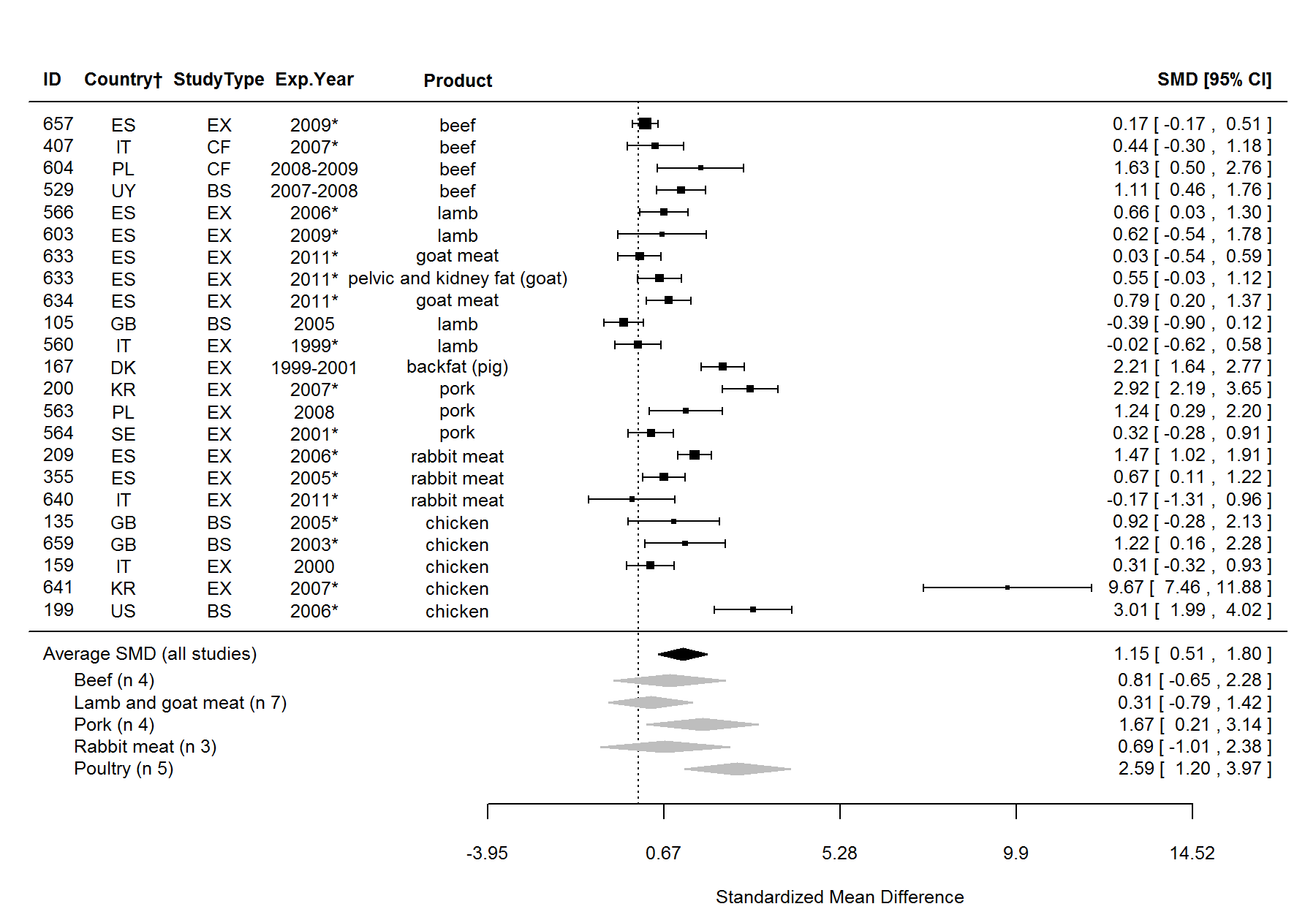


**Figure S19.** Forest plot showing the results of the comparison of polyunsaturated fatty acids (PUFA) content between organic and conventional meat using standardised mean differences (SMDs) with 95% confidence intervals, for studies included in standard meta-analysis. The estimated average SMD for all studies and SMDs for different animal groups are indicated at the bottom of the figure. Sign of the SMD indicates if the analysed parameter is higher (+) or lower (-) in organic foods. ID, Paper unique identification number (see supplementary Table S2 for references); CF, comparison of farms, BS, basket study, EX, controlled experiment. *No information about the experimental year (estimated as publication year - 2), †Country codes according ISO 3166-2 (see [*http://www.iso.org/iso/home/standards/country_codes.htm*](http://www.iso.org/iso/home/standards/country_codes.htm)).

*
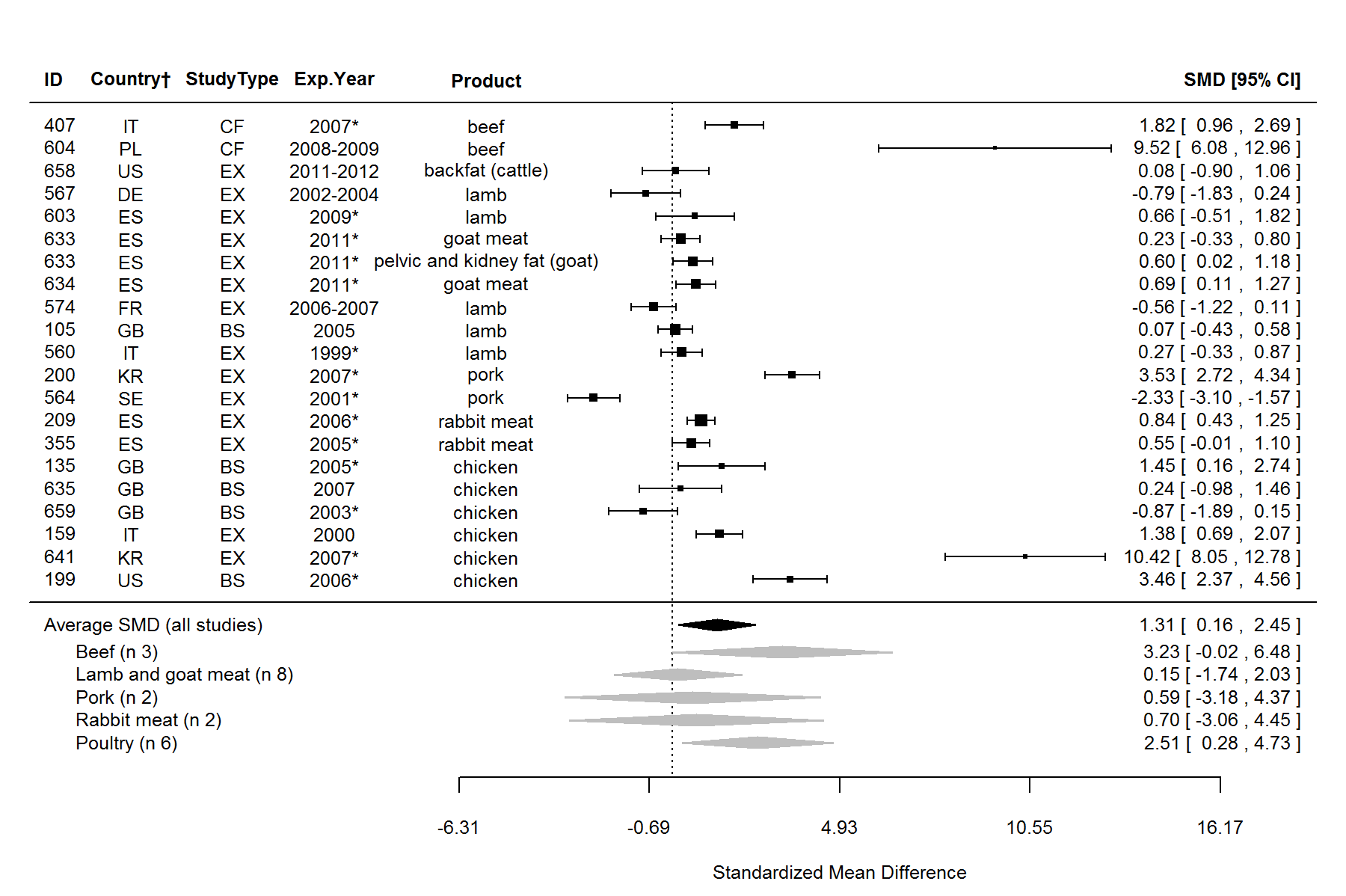
*

**Figure S20.** Forest plot showing the results of the comparison of *n*-3 fatty acids content between organic and conventional meat using standardised mean differences (SMDs) with 95% confidence intervals, for studies included in standard meta-analysis. The estimated average SMD for all studies and SMDs for different animal groups are indicated at the bottom of the figure. Sign of the SMD indicates if the analysed parameter is higher (+) or lower (-) in organic foods. ID, Paper unique identification number (see supplementary Table S2 for references); CF, comparison of farms, BS, basket study, EX, controlled experiment. *No information about the experimental year (estimated as publication year - 2), †Country codes according ISO 3166-2 (see [*http://www.iso.org/iso/home/standards/country_codes.htm*](http://www.iso.org/iso/home/standards/country_codes.htm)).


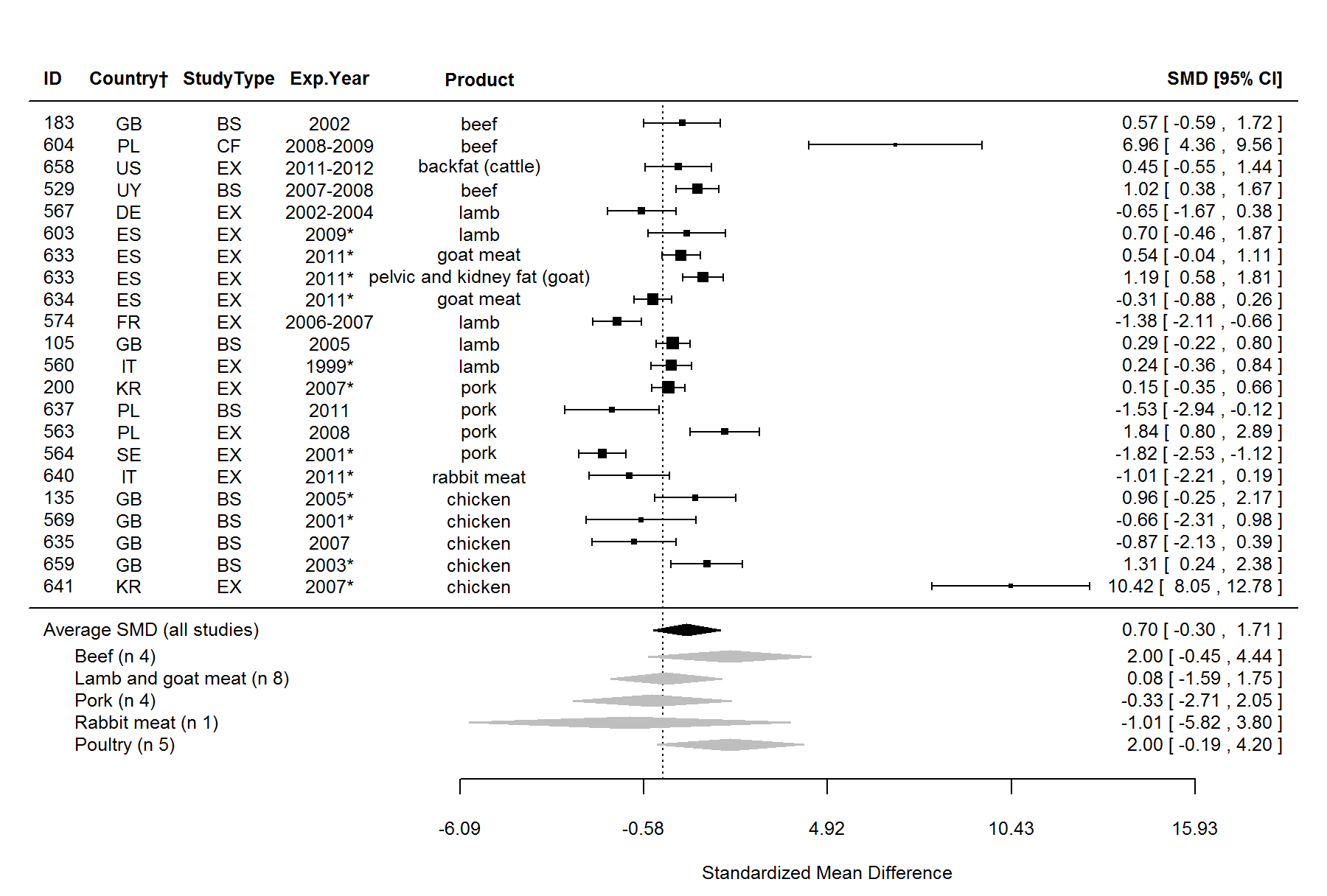


**Figure S21.** Forest plot showing the results of the comparison of α-linolenic acid (ALA, cis-9,12,15-18:3) content between organic and conventional meat using standardised mean differences (SMDs) with 95% confidence intervals, for studies included in standard meta-analysis. The estimated average SMD for all studies and SMDs for different animal groups are indicated at the bottom of the figure. Sign of the SMD indicates if the analysed parameter is higher (+) or lower (-) in organic foods. ID, Paper unique identification number (see supplementary Table S2 for references); CF, comparison of farms, BS, basket study, EX, controlled experiment. *No information about the experimental year (estimated as publication year - 2), †Country codes according ISO 3166-2 (see [*http://www.iso.org/iso/home/standards/country_codes.htm*](http://www.iso.org/iso/home/standards/country_codes.htm)).


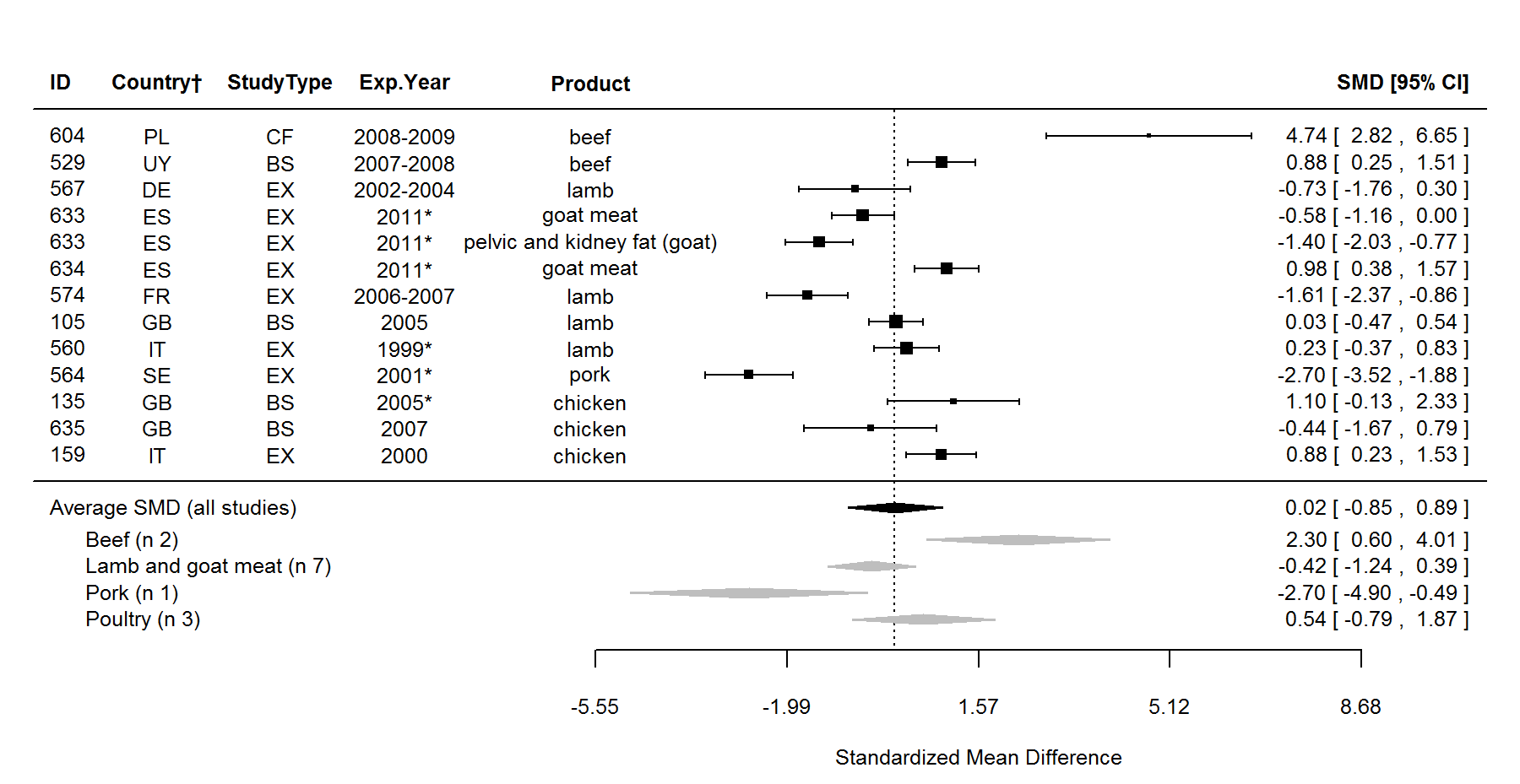


**Figure S22.** Forest plot showing the results of the comparison of eicosapentaenoic acid (EPA, cis-5,8,11,14,17-20:5) content between organic and conventional meat using standardised mean differences (SMDs) with 95% confidence intervals, for studies included in standard meta-analysis. The estimated average SMD for all studies and SMDs for different animal groups are indicated at the bottom of the figure. Sign of the SMD indicates if the analysed parameter is higher (+) or lower (-) in organic foods. ID, Paper unique identification number (see supplementary Table S2 for references); CF, comparison of farms, BS, basket study, EX, controlled experiment. *No information about the experimental year (estimated as publication year - 2), †Country codes according ISO 3166-2 (see [*http://www.iso.org/iso/home/standards/country_codes.htm*](http://www.iso.org/iso/home/standards/country_codes.htm)).


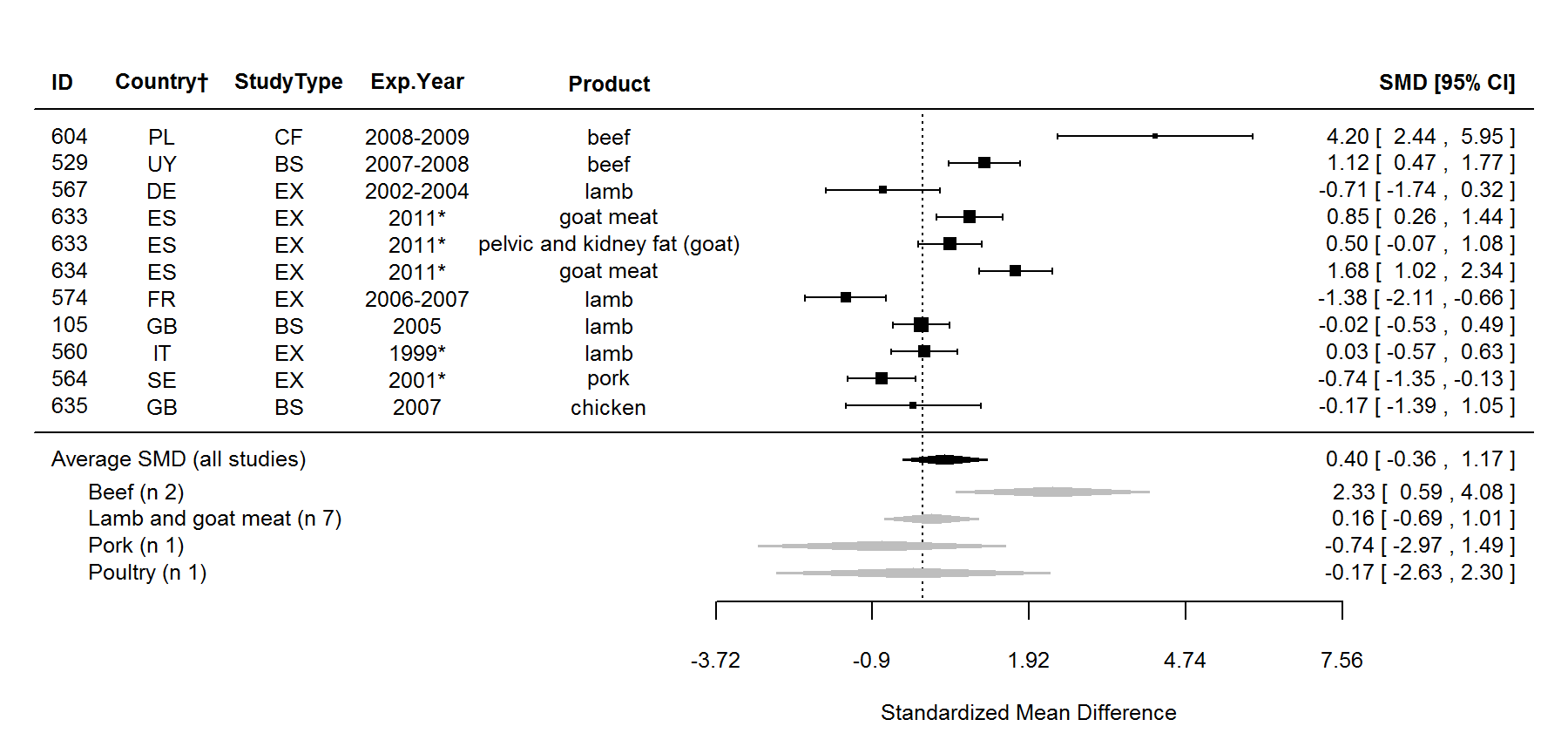


**Figure S23.** Forest plot showing the results of the comparison of docosapentaenoic acid (DPA, cis-7,10,13,16,19-22:5) content between organic and conventional meat using standardised mean differences (SMDs) with 95% confidence intervals, for studies included in standard meta-analysis. The estimated average SMD for all studies and SMDs for different animal groups are indicated at the bottom of the figure. Sign of the SMD indicates if the analysed parameter is higher (+) or lower (-) in organic foods. ID, Paper unique identification number (see supplementary Table S2 for references); CF, comparison of farms, BS, basket study, EX, controlled experiment. *No information about the experimental year (estimated as publication year - 2), †Country codes according ISO 3166-2 (see [*http://www.iso.org/iso/home/standards/country_codes.htm*](http://www.iso.org/iso/home/standards/country_codes.htm)).


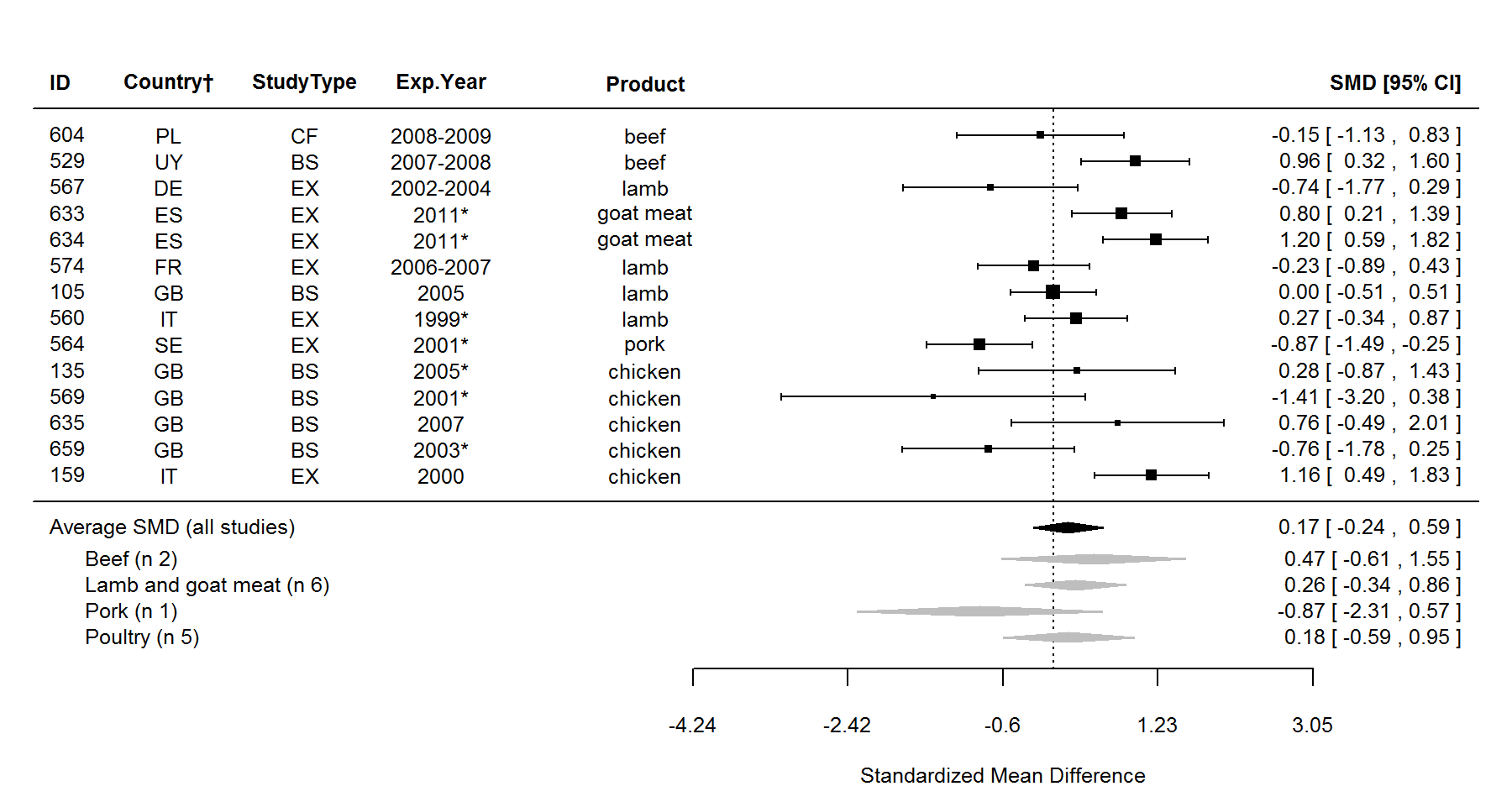


**Figure S24.** Forest plot showing the results of the comparison of docosahexaenoic acid (DHA, cis-4,7,10,13,16,19-22:6) content between organic and conventional meat using standardised mean differences (SMDs) with 95% confidence intervals, for studies included in standard meta-analysis. The estimated average SMD for all studies and SMDs for different animal groups are indicated at the bottom of the figure. Sign of the SMD indicates if the analysed parameter is higher (+) or lower (-) in organic foods. ID, Paper unique identification number (see supplementary Table S2 for references); CF, comparison of farms, BS, basket study, EX, controlled experiment. *No information about the experimental year (estimated as publication year - 2), †Country codes according ISO 3166-2 (see [*http://www.iso.org/iso/home/standards/country_codes.htm*](http://www.iso.org/iso/home/standards/country_codes.htm)).


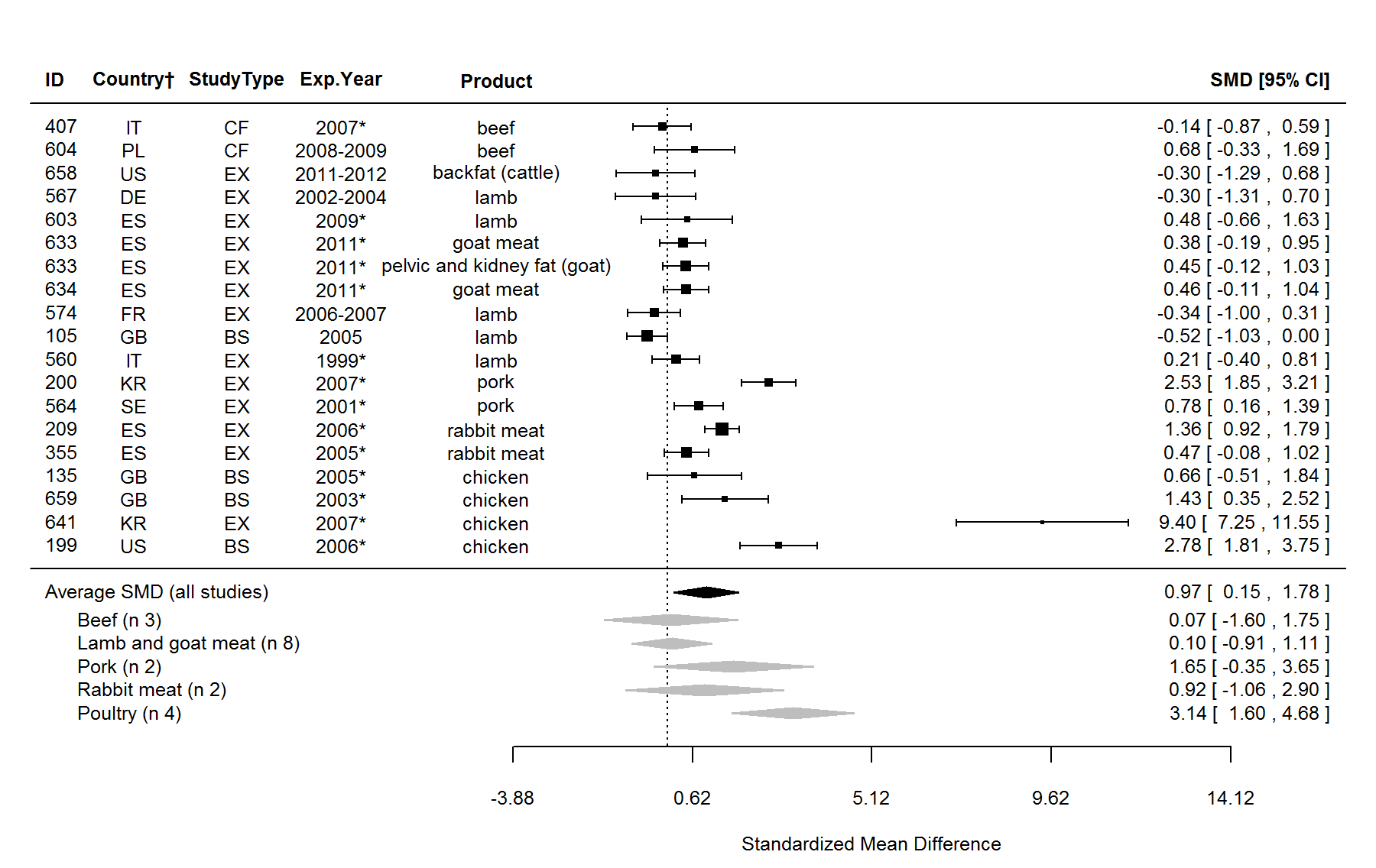


**Figure S25.** Forest plot showing the results of the comparison of *n*-6 fatty acid content between organic and conventional meat using standardised mean differences (SMDs) with 95% confidence intervals, for studies included in standard meta-analysis. The estimated average SMD for all studies and SMDs for different animal groups are indicated at the bottom of the figure. Sign of the SMD indicates if the analysed parameter is higher (+) or lower (-) in organic foods. ID, Paper unique identification number (see supplementary Table S2 for references); CF, comparison of farms, BS, basket study, EX, controlled experiment. *No information about the experimental year (estimated as publication year - 2), †Country codes according ISO 3166-2 (see [*http://www.iso.org/iso/home/standards/country_codes.htm*](http://www.iso.org/iso/home/standards/country_codes.htm)).


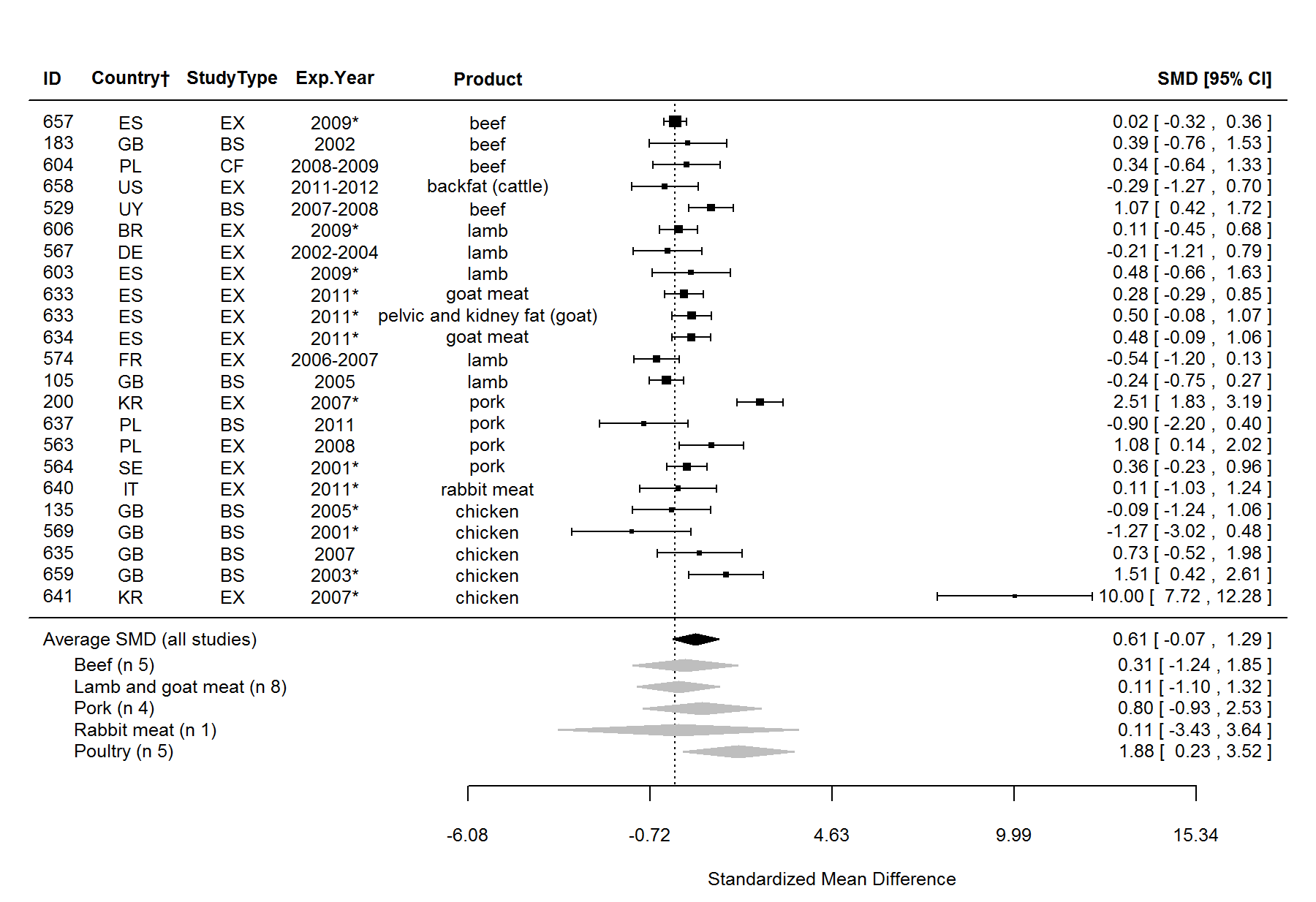


**Figure S26.** Forest plot showing the results of the comparison of linoleic acid (LA, cis-9,12-18:2) content between organic and conventional meat using standardised mean differences (SMDs) with 95% confidence intervals, for studies included in standard meta-analysis. The estimated average SMD for all studies and SMDs for different animal groups are indicated at the bottom of the figure. Sign of the SMD indicates if the analysed parameter is higher (+) or lower (-) in organic foods. ID, Paper unique identification number (see supplementary Table S2 for references); CF, comparison of farms, BS, basket study, EX, controlled experiment. *No information about the experimental year (estimated as publication year - 2), †Country codes according ISO 3166-2 (see [*http://www.iso.org/iso/home/standards/country_codes.htm*](http://www.iso.org/iso/home/standards/country_codes.htm)).


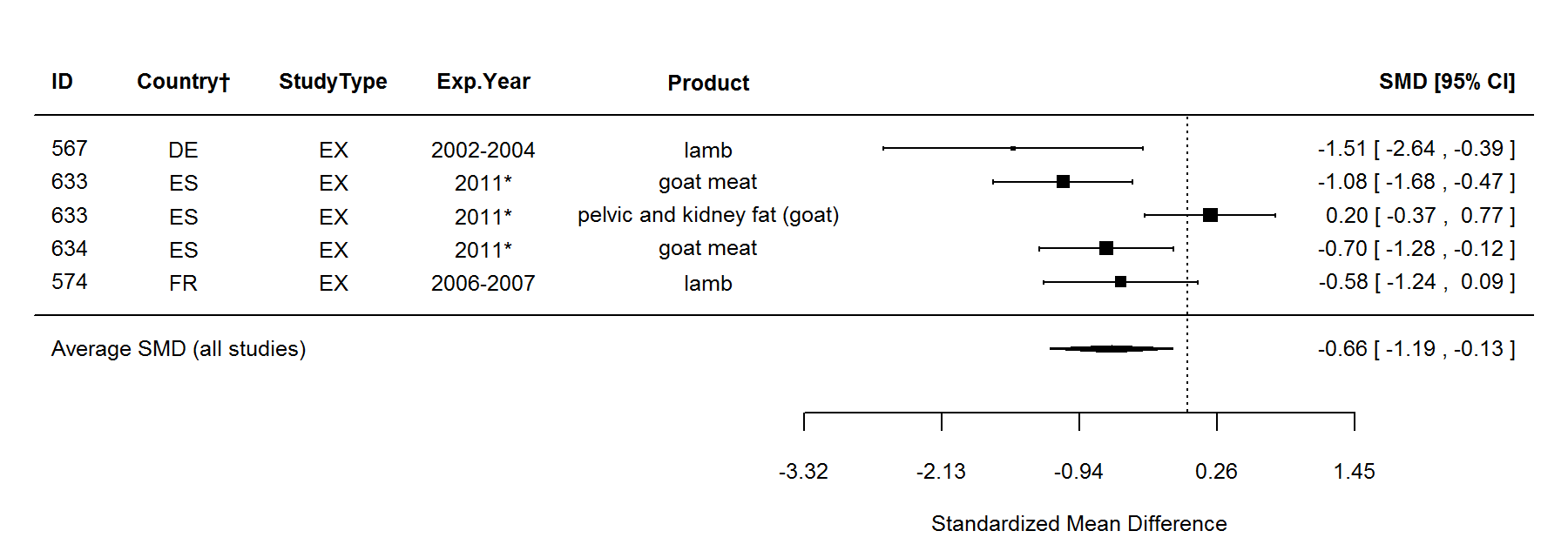


**Figure S27.** Forest plot showing the results of the comparison of conjugated linoleic acid (CLA, cis-9-trans-11-18:2) content between organic and conventional meat using standardised mean differences (SMDs) with 95% confidence intervals, for studies included in standard meta-analysis. The estimated average SMD for all studies and SMDs for different animal groups are indicated at the bottom of the figure. Sign of the SMD indicates if the analysed parameter is higher (+) or lower (-) in organic foods. ID, Paper unique identification number (see supplementary Table S2 for references); CF, comparison of farms, BS, basket study, EX, controlled experiment. *No information about the experimental year (estimated as publication year - 2), †Country codes according ISO 3166-2 (see [*http://www.iso.org/iso/home/standards/country_codes.htm*](http://www.iso.org/iso/home/standards/country_codes.htm)).


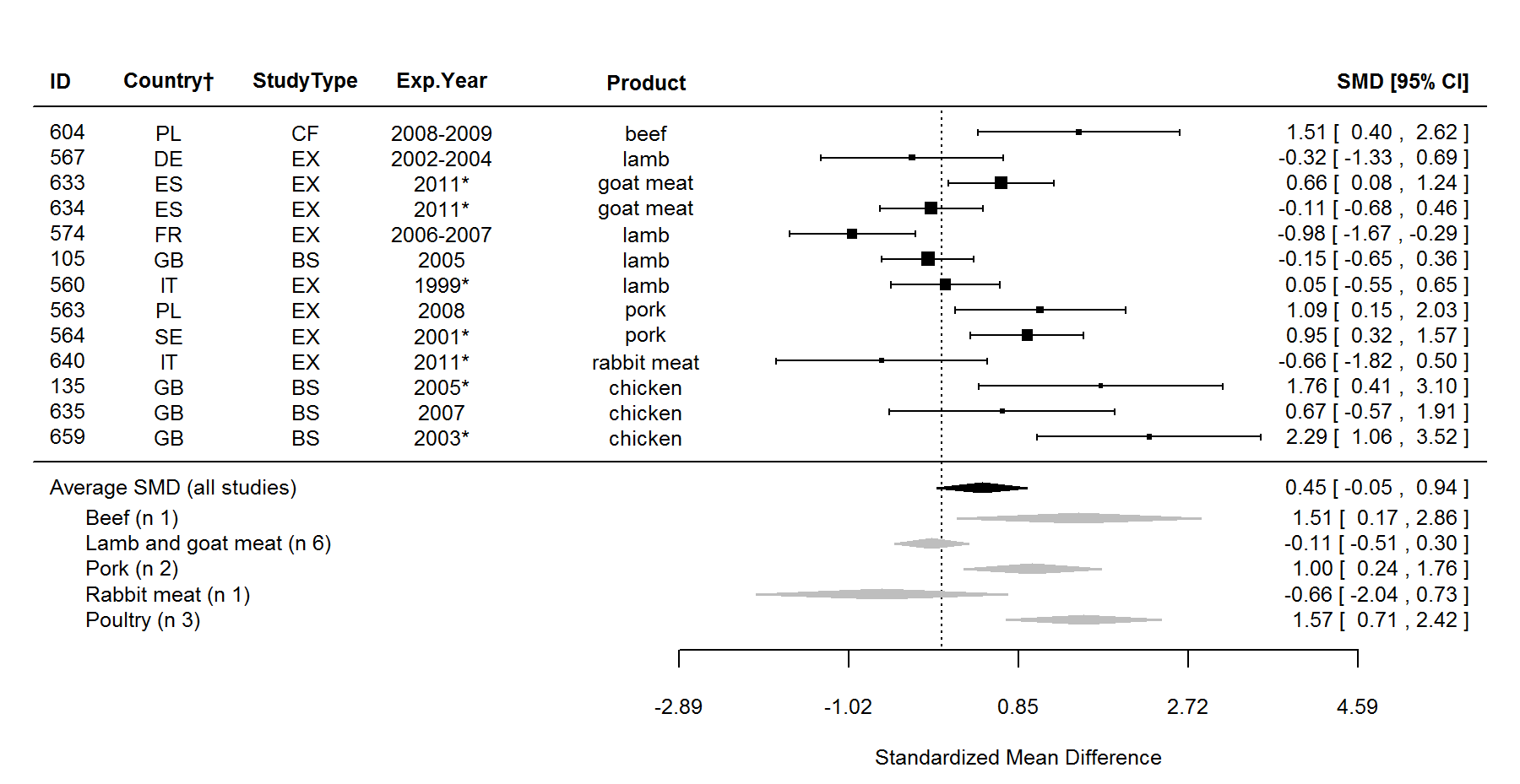


**Figure S28.** Forest plot showing the results of the comparison of arachidonic acid (AA, cis-5,8,11,14-20:4) content between organic and conventional meat using standardised mean differences (SMDs) with 95% confidence intervals, for studies included in standard meta-analysis. The estimated average SMD for all studies and SMDs for different animal groups are indicated at the bottom of the figure. Sign of the SMD indicates if the analysed parameter is higher (+) or lower (-) in organic foods. ID, Paper unique identification number (see supplementary Table S2 for references); CF, comparison of farms, BS, basket study, EX, controlled experiment. *No information about the experimental year (estimated as publication year - 2), †Country codes according ISO 3166-2 (see [*http://www.iso.org/iso/home/standards/country_codes.htm*](http://www.iso.org/iso/home/standards/country_codes.htm)).

*
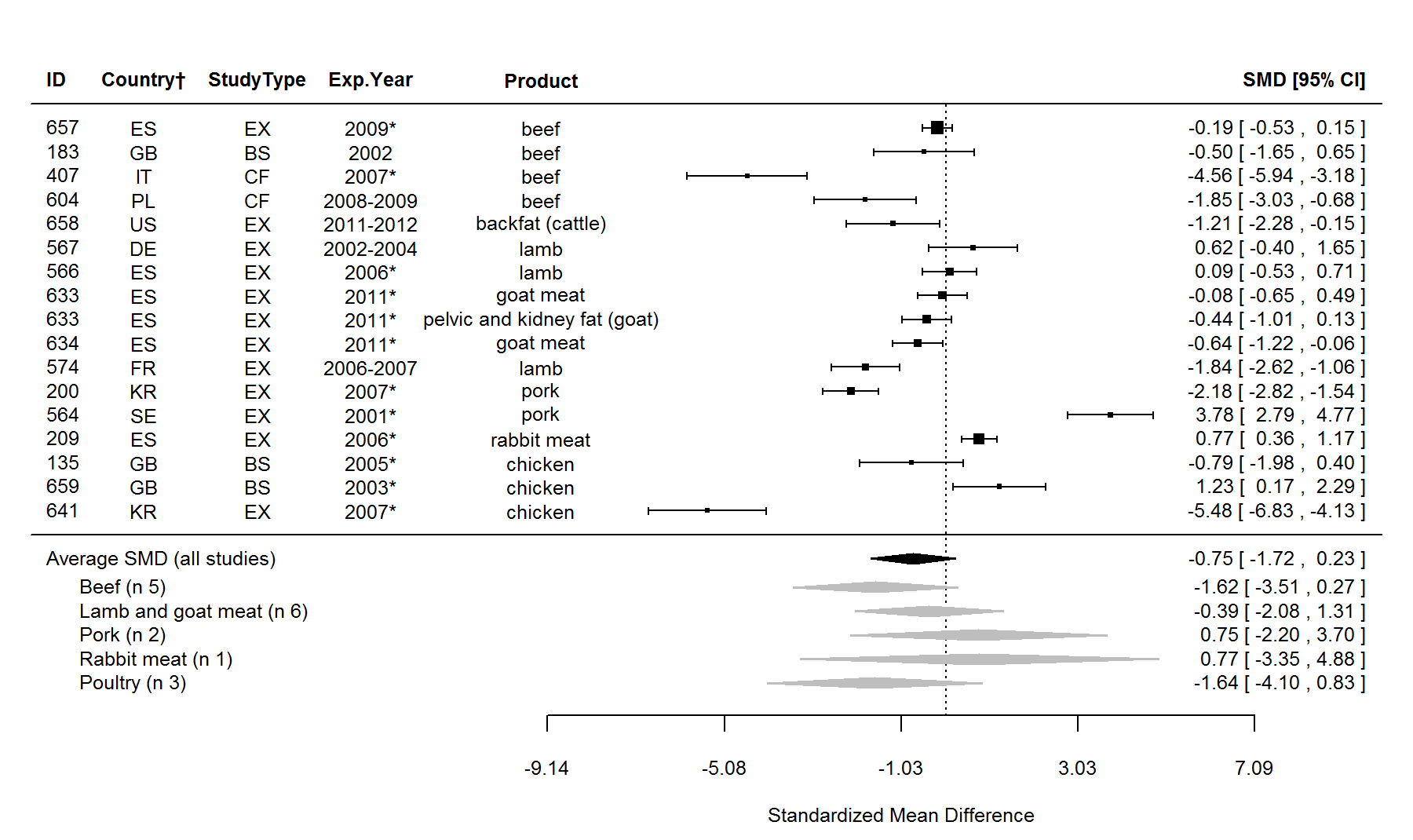
*

**Figure S29.** Forest plot showing the results of the comparison of *n*-6/-3 ratio between organic and conventional meat using standardised mean differences (SMDs) with 95% confidence intervals, for studies included in standard meta-analysis. The estimated average SMD for all studies and SMDs for different animal groups are indicated at the bottom of the figure. Sign of the SMD indicates if the analysed parameter is higher (+) or lower (-) in organic foods. ID, Paper unique identification number (see supplementary Table S2 for references); CF, comparison of farms, BS, basket study, EX, controlled experiment. *No information about the experimental year (estimated as publication year - 2), †Country codes according ISO 3166-2 (see [*http://www.iso.org/iso/home/standards/country_codes.htm*](http://www.iso.org/iso/home/standards/country_codes.htm)).


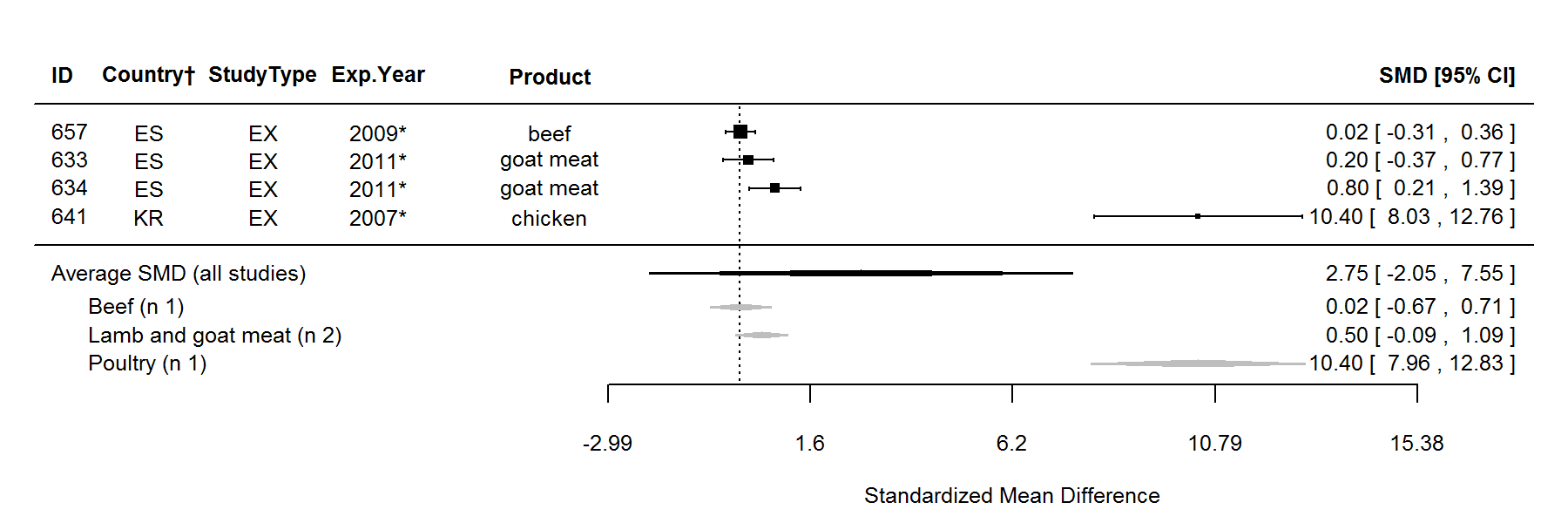


**Figure S30.** Forest plot showing the results of the comparison of polyunsaturated to saturated fatty acids (PUFA/SFA) ratio between organic and conventional meat using standardised mean differences (SMDs) with 95% confidence intervals, for studies included in standard meta-analysis. The estimated average SMD for all studies and SMDs for different animal groups are indicated at the bottom of the figure. Sign of the SMD indicates if the analysed parameter is higher (+) or lower (-) in organic foods. ID, Paper unique identification number (see supplementary Table S2 for references); CF, comparison of farms, BS, basket study, EX, controlled experiment. *No information about the experimental year (estimated as publication year - 2), †Country codes according ISO 3166-2 (see [*http://www.iso.org/iso/home/standards/country_codes.htm*](http://www.iso.org/iso/home/standards/country_codes.htm)).


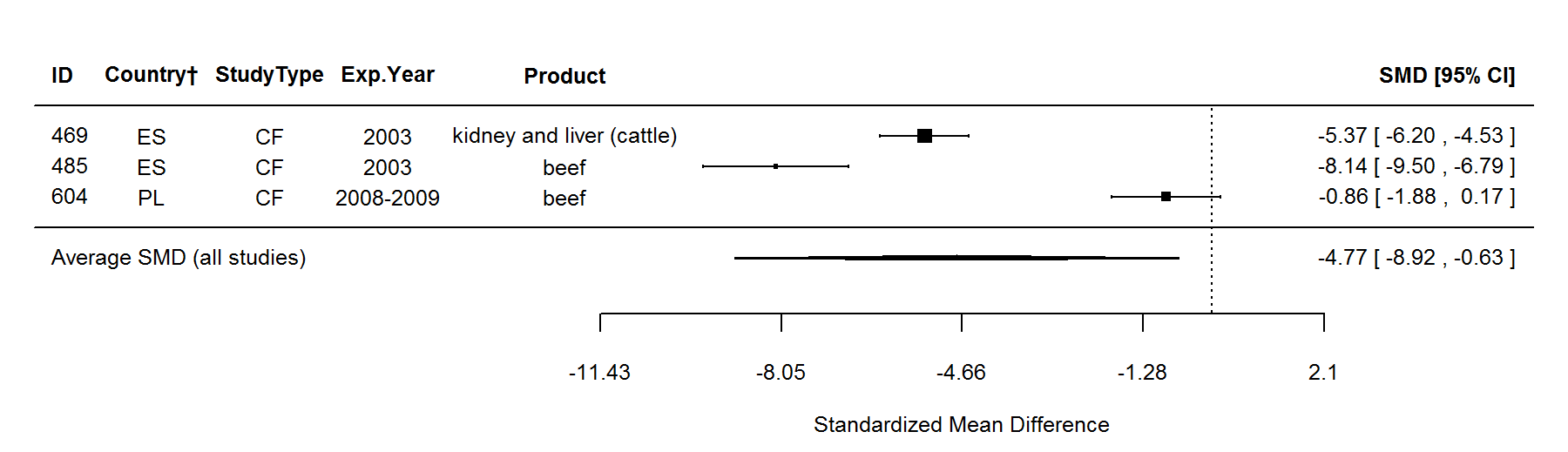


**Figure S31.** Forest plot showing the results of the comparison of copper (Cu) content between organic and conventional meat using standardised mean differences (SMDs) with 95% confidence intervals, for studies included in standard meta-analysis. The estimated average SMD for all studies and SMDs for different animal groups are indicated at the bottom of the figure. Sign of the SMD indicates if the analysed parameter is higher (+) or lower (-) in organic foods. ID, Paper unique identification number (see supplementary Table S2 for references); CF, comparison of farms, BS, basket study, EX, controlled experiment. *No information about the experimental year (estimated as publication year - 2), †Country codes according ISO 3166-2 (see [*http://www.iso.org/iso/home/standards/country_codes.htm*](http://www.iso.org/iso/home/standards/country_codes.htm)).


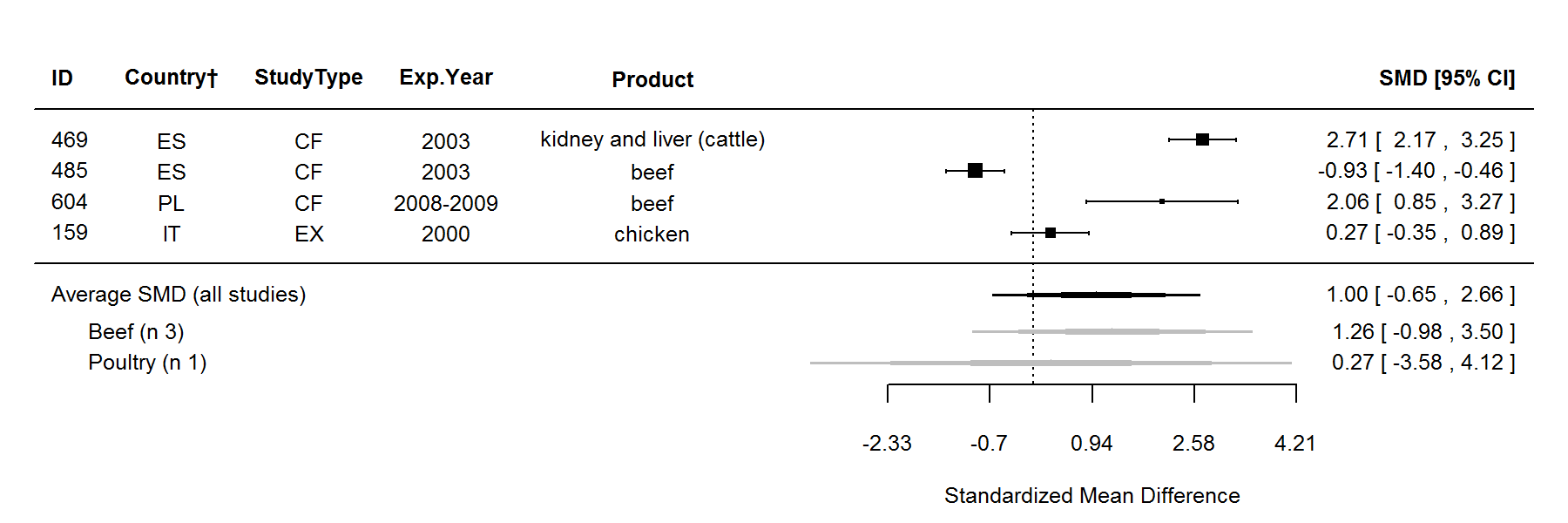


**Figure S32.** Forest plot showing the results of the comparison of iron (Fe) content between organic and conventional meat using standardised mean differences (SMDs) with 95% confidence intervals, for studies included in standard meta-analysis. The estimated average SMD for all studies and SMDs for different animal groups are indicated at the bottom of the figure. Sign of the SMD indicates if the analysed parameter is higher (+) or lower (-) in organic foods. ID, Paper unique identification number (see supplementary Table S2 for references); CF, comparison of farms, BS, basket study, EX, controlled experiment. *No information about the experimental year (estimated as publication year - 2), †Country codes according ISO 3166-2 (see [*http://www.iso.org/iso/home/standards/country_codes.htm*](http://www.iso.org/iso/home/standards/country_codes.htm)).


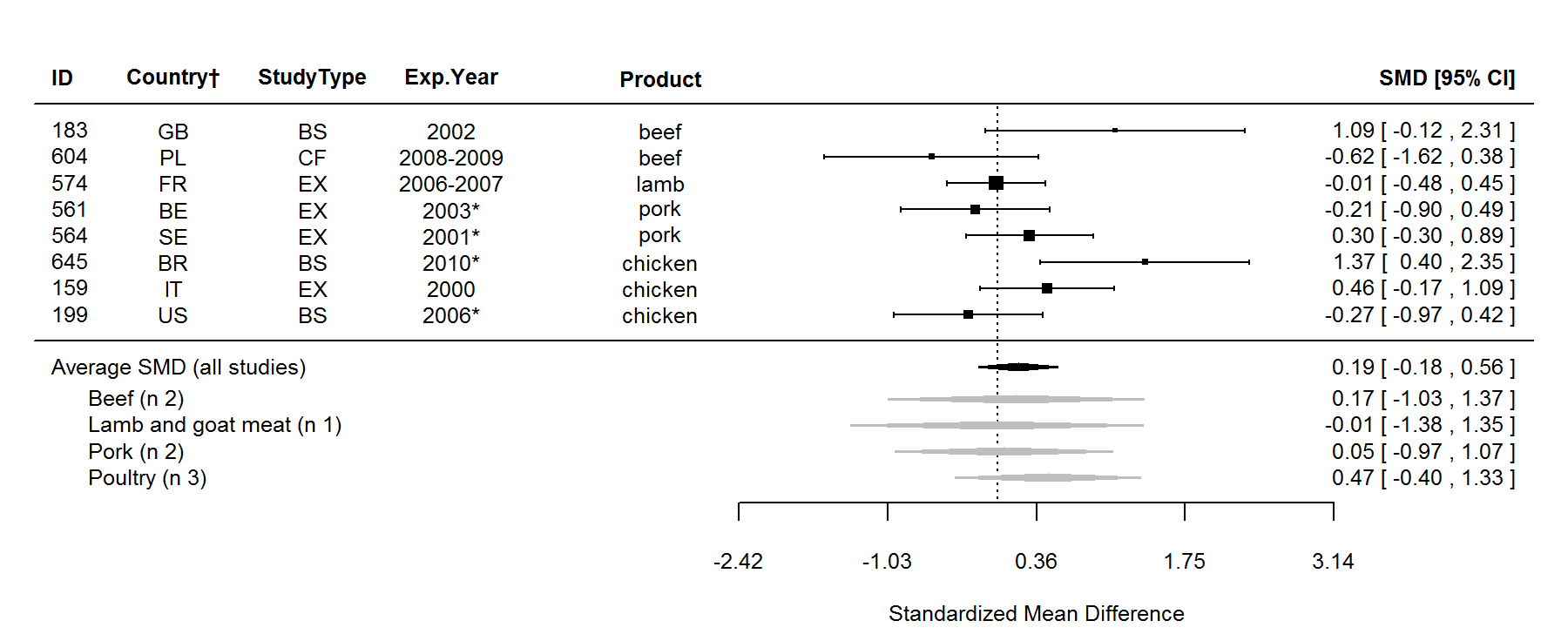


**Figure S33.** Forest plot showing the results of the comparison of lipid oxidation (TBARS) between organic and conventional meat using standardised mean differences (SMDs) with 95% confidence intervals, for studies included in standard meta-analysis. The estimated average SMD for all studies and SMDs for different animal groups are indicated at the bottom of the figure. Sign of the SMD indicates if the analysed parameter is higher (+) or lower (-) in organic foods. ID, Paper unique identification number (see supplementary Table S2 for references); CF, comparison of farms, BS, basket study, EX, controlled experiment. *No information about the experimental year (estimated as publication year - 2), †Country codes according ISO 3166-2 (see [*http://www.iso.org/iso/home/standards/country_codes.htm*](http://www.iso.org/iso/home/standards/country_codes.htm)).


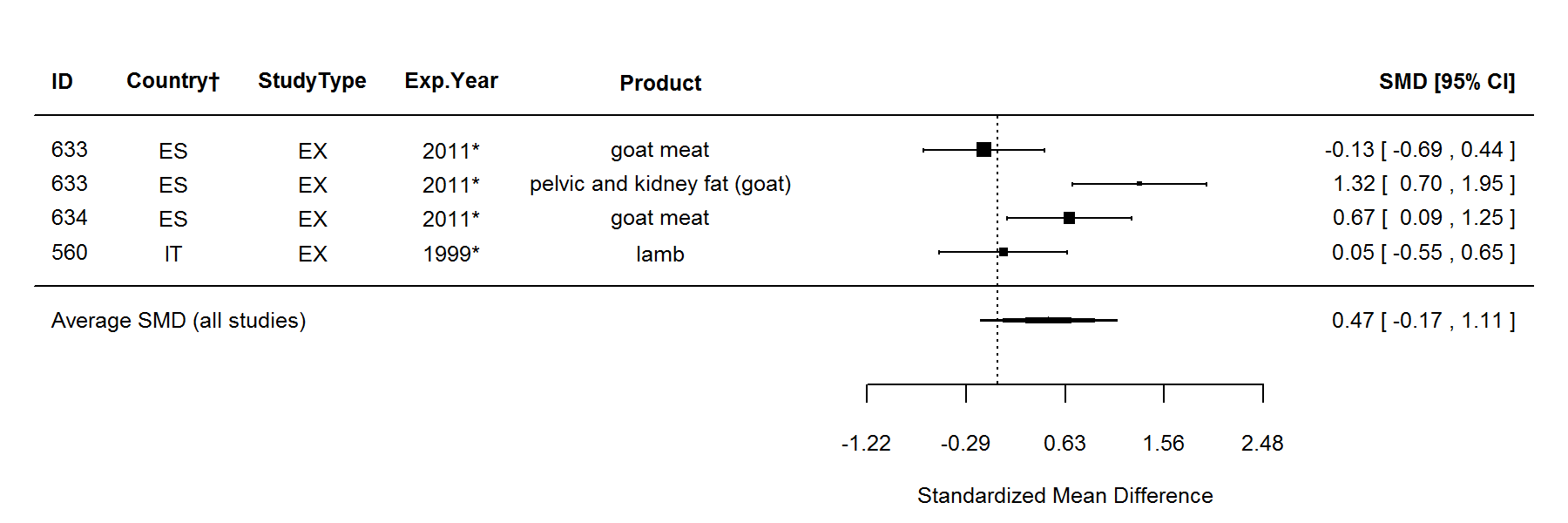


**Figure S34.** Forest plot showing the results of the comparison of atherogenicity index between organic and conventional meat using standardised mean differences (SMDs) with 95% confidence intervals, for studies included in standard meta-analysis. The estimated average SMD for all studies and SMDs for different animal groups are indicated at the bottom of the figure. Sign of the SMD indicates if the analysed parameter is higher (+) or lower (-) in organic foods. ID, Paper unique identification number (see supplementary Table S2 for references); CF, comparison of farms, BS, basket study, EX, controlled experiment. *No information about the experimental year (estimated as publication year - 2), †Country codes according ISO 3166-2 (see [*http://www.iso.org/iso/home/standards/country_codes.htm*](http://www.iso.org/iso/home/standards/country_codes.htm)).


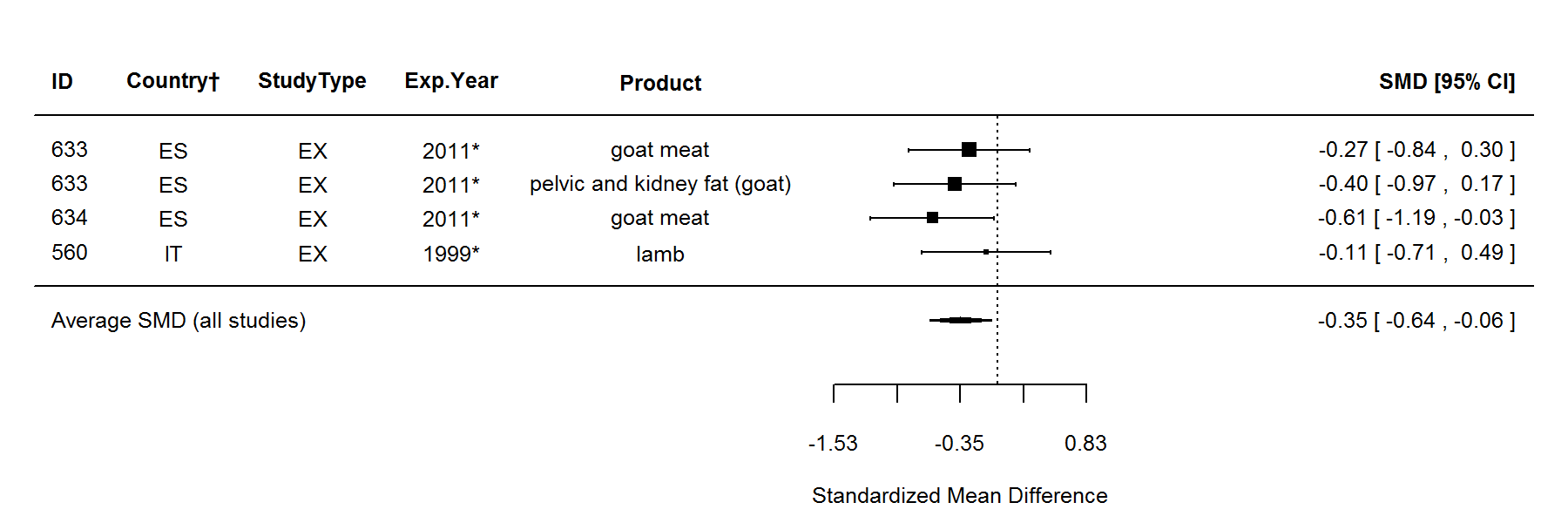


**Figure S35.** Forest plot showing the results of the comparison of thrombogenicity index between organic and conventional meat using standardised mean differences (SMDs) with 95% confidence intervals, for studies included in standard meta-analysis. The estimated average SMD for all studies and SMDs for different animal groups are indicated at the bottom of the figure. Sign of the SMD indicates if the analysed parameter is higher (+) or lower (-) in organic foods. ID, Paper unique identification number (see supplementary Table S2 for references); CF, comparison of farms, BS, basket study, EX, controlled experiment. *No information about the experimental year (estimated as publication year - 2), †Country codes according ISO 3166-2 (see [*http://www.iso.org/iso/home/standards/country_codes.htm*](http://www.iso.org/iso/home/standards/country_codes.htm)).

| **Table S12.** Results of the standard meta-analysis and sensitivity meta-analysis 1 for parameters where none of the meta-analyses protocols detected significant differences between organic and conventional meat. | | | | | | | | | |
| --- | --- | --- | --- | --- | --- | --- | --- | --- | --- |
|  | **Standard meta-analysis** | | | | |  | **Sensitivity meta-analysis 1** | | |
| **Parameter** | ***n*** | **SMD** | **95% CI** | ***P**** | **Heterogeneity**† |  | ***n*** | **Ln ratio**‡ | ***P**** |
| *Major components* |  |  |  |  |  |  |  |  |  |
| Ash | 12 | -0.12 | -0.83, 0.60 | 0.750 | Yes (91%) |  | 17 | 4.62 | 0.353 |
| Dry mass | 5 | -0.29 | -0.78, 0.19 | 0.238 | Yes (56%) |  | 8 | 4.60 | 0.229 |
| Water | 12 | 0.22 | -0.56, 0.99 | 0.584 | Yes (93%) |  | 16 | 4.61 | 0.247 |
| *Fatty acids* |  |  |  |  |  |  |  |  |  |
| 10:0 (capric acid) | 4 | -0.34 | -0.84, 0.15 | 0.169 | Yes (38%) |  | 7 | 4.52 | 0.154 |
| 15:0 (pentadecanoic acid) | 10 | -0.13 | -0.76, 0.50 | 0.682 | Yes (86%) |  | 13 | 4.56 | 0.271 |
| 17:0 (heptadecanoic acid) | 10 | 0.19 | -0.26, 0.64 | 0.410 | Yes (74%) |  | 15 | 4.58 | 0.302 |
| 18:0 (stearic acid) | 24 | -0.21 |  | 0.116 | Yes (67%) |  | 30 | 4.55 | 0.124 |
| 21:0 | 3 | 0.28 | -0.63, 1.19 | 0.543 | Yes (80%) |  | 5 | 4.58 | 0.444 |
| 22:0 | - | - | - | - | - |  | 3 | 5.13 | 0.247 |
| 23:0 (cerotic acid) | - | - | - | - | - |  | 3 | 4.37 | 0.496 |
| 24:0 (lignoceric acid) | 3 | 0.29 | -0.18, 0.77 | 0.220 | No (0%) |  | 3 | 4.83 | 0.133 |
| 16:1 (palmitoleic acid) | 18 | -0.10 |  | 0.443 | Yes (53%) |  | 23 | 4.55 | 0.182 |
| 16:1 n-7 | 5 | -1.17 | -2.48, 0.15 | 0.081 | Yes (92%) |  | 5 | 4.49 | 0.220 |
| 16:1 n-9 | 4 | 0.20 | -0.29, 0.70 | 0.417 | Yes (56%) |  | 5 | 4.67 | 0.096 |
| 18:1 n-7 | 3 | 0.18 | -0.47, 0.83 | 0.589 | Yes (43%) |  | 3 | 4.63 | 0.386 |
| trans-18:1 (total) | 3 | -0.09 | -1.13, 0.94 | 0.863 | Yes (89%) |  | 5 | 4.52 | 0.186 |
| *n*, number of data points included in the comparison; SMD, standardised mean difference of random-effect model; CI, 95% confidence intervals. **P* value <0.05 indicates significance of the difference in composition between organic and conventional meat; †Heterogeneity and the I^2^ Statistic; ‡Ln ratio = Ln(ORG/CONV × 100%). | | | | | | | | | |

| **Table S12 cont.** Results of the standard meta-analysis and sensitivity meta-analysis 1 for parameters where none of the meta-analyses protocols detected significant differences between organic and conventional meat. | | | | | | | | | |
| --- | --- | --- | --- | --- | --- | --- | --- | --- | --- |
|  | **Standard meta-analysis** | | | | |  | **Sensitivity meta-analysis 1** | | |
| **Parameter** | ***n*** | **SMD** | **95% CI** | ***P**** | **Heterogeneity**† |  | ***n*** | **Ln ratio**‡ | ***P**** |
| trans-18:1 n-9 | - | - | - | - | - |  | 3 | 4.91 | 0.385 |
| trans-9-18:1 | - | - | - | - | - |  | 5 | 4.51 | 0.248 |
| VA (trans-11-18:1) | 5 | -0.19 | -0.98, 0.60 | 0.642 | Yes (82%) |  | 7 | 4.49 | 0.198 |
| 20:1 n-9 | - | - | - | - | - |  | 3 | 3.95 | 0.125 |
| cis-11-20:1 (eicosenoic acid) | 8 | -0.16 | -0.93, 0.61 | 0.685 | Yes (87%) |  | 9 | 4.43 | 0.091 |
| CLA (total) | 6 | 0.39 | -0.53, 1.30 | 0.408 | Yes (91%) |  | 7 | 4.73 | 0.347 |
| CLA (trans-10-cis-12-18:2) | - | - | - | - | - |  | 4 | 4.44 | 0.187 |
| CLA index | 3 | -0.14 | -1.09, 0.80 | 0.763 | Yes (87%) |  | 4 | 4.60 | 0.502 |
| 20:2 | - | - | - | - | - |  | 3 | 4.49 | 0.511 |
| cis-11,14-20:2 n-6 | 4 | 0.19 | -0.92, 1.30 | 0.739 | Yes (91%) |  | 6 | 4.71 | 0.208 |
| GLA (cis-6,9,12-18:3) | 7 | 0.02 | -0.52, 0.57 | 0.933 | Yes (72%) |  | 9 | 4.61 | 0.480 |
| DGLA (cis-8,11,14-20:3) | 10 | 0.07 |  | 0.715 | Yes (60%) |  | 14 | 4.54 | 0.235 |
| ETE (cis-11,14,17-20:3) | 4 | -0.18 | -0.47, 0.10 | 0.213 | No (0%) |  | 10 | 4.73 | 0.202 |
| DTA (cis-7,10,13,16-22:4) | 9 | 1.04 | -0.14, 2.21 | 0.083 | Yes (96%) |  | 12 | 4.92 | 0.204 |
| USFA | 6 | 1.37 | -0.29, 3.04 | 0.106 | Yes (97%) |  | 9 | 4.62 | 0.187 |
| USFA/SFA ratio | 3 | 0.04 | -0.28, 0.37 | 0.795 | No (0%) |  | 4 | 4.60 | 0.374 |
| Δ-9 desaturase 16:1/16:0 activity index | 3 | 0.76 | -1.44, 2.95 | 0.499 | Yes (97%) |  | 5 | 5.08 | 0.500 |
| Δ-9 desaturase 18:1/18:0 activity index§ | - | - | - | - | - |  | 4 | 4.63 | 0.123 |
| *Vitamins and antioxidants* |  |  |  |  |  |  |  |  |  |
| α-tocopherol (total) | 6 | 1.55 | -2.12, 5.22 | 0.408 | Yes (99%) |  | 7 | 4.46 | 0.174 |
| *n*, number of data points included in the comparison; SMD, standardised mean difference of random-effect model; CI, 95% confidence intervals; CLA, conjugated linoleic acids; DGLA, dihomo-γ-linolenic acid; DTA, docosatetraenoic acid; ETE, eicosatrienoic acid; GLA, γ-Linolenic acid; USFA, unsaturated fatty acids; SFA, saturated fatty acids; VA, vaccenic acid. **P* value <0.05 indicates significance of the difference in composition between organic and conventional meat; †Heterogeneity and the I^2^ Statistic; ‡Ln ratio = Ln(ORG/CONV × 100%); §Outlying data pairs (where the MPD between ORG and CONV was over fifty times greater than the mean value including the outliers) were removed. | | | | | | | | | |

| **Table S12 cont.** Results of the standard meta-analysis and sensitivity meta-analysis 1 for parameters where none of the meta-analyses protocols detected significant differences between organic and conventional meat. | | | | | | | | | |
| --- | --- | --- | --- | --- | --- | --- | --- | --- | --- |
|  | **Standard meta-analysis** | | | | |  | **Sensitivity meta-analysis 1** | | |
| **Parameter** | ***n*** | **SMD** | **95% CI** | ***P**** | **Heterogeneity**† |  | ***n*** | **Ln ratio**‡ | ***P**** |
| *Minerals and undesirable metals* |  |  |  |  |  |  |  |  |  |
| Arsenic (As) | - | - | - | - | - |  | 4 | 5.76 | 0.504 |
| Cadmium (Cd) | 4 | -0.02 | -0.54, 0.49 | 0.928 | Yes (75%) |  | 7 | 4.37 | 0.076 |
| Iron (Fe) (in haemoglobin) | 3 | 1.04 | -0.88, 2.96 | 0.289 | Yes (95%) |  | 3 | 4.85 | 0.250 |
| Lead (Pb) | - | - | - | - | - |  | 5 | 4.39 | 0.255 |
| Zinc (Zn) | 5 | 0.49 | -0.50, 1.48 | 0.330 | Yes (94%) |  | 6 | 4.68 | 0.110 |
| *Pesticides, mycotoxins and other contaminants* |  |  |  |  |  |  |  |  |  |
| 4-4’-DDD | - | - | - | - | - |  | 3 | 4.61 | 1.000 |
| 4-4’-DDE | - | - | - | - | - |  | 3 | 3.71 | 0.124 |
| 4-4’-DDT | - | - | - | - | - |  | 3 | 4.61 | 1.000 |
| Aldrin | - | - | - | - | - |  | 3 | 4.61 | 1.000 |
| Chlorpyrifos | - | - | - | - | - |  | 3 | 4.57 | 0.500 |
| Diazinon | - | - | - | - | - |  | 3 | 5.26 | 0.492 |
| Dieldrin | - | - | - | - | - |  | 3 | 4.61 | 1.000 |
| Disyston | - | - | - | - | - |  | 3 | 4.61 | 1.000 |
| Endrin | - | - | - | - | - |  | 4 | 4.61 | 1.000 |
| Ethion | - | - | - | - | - |  | 3 | 4.61 | 1.000 |
| Ethyl parathion | - | - | - | - | - |  | 3 | 4.61 | 1.000 |
| *n*, number of data points included in the comparison; SMD, standardised mean difference of random-effect model; CI, 95% confidence intervals; DDD, dichlorodiphenyldichloroethane; DDE, dichlorodiphenyldichloroethylene; DDT, dichlorodiphenyltrichloroethane. **P* value <0.05 indicates significance of the difference in composition between organic and conventional meat; †Heterogeneity and the I^2^ Statistic; ‡Ln ratio = Ln(ORG/CONV × 100%). | | | | | | | | | |

| **Table S12 cont.** Results of the standard meta-analysis and sensitivity meta-analysis 1 for parameters where none of the meta-analyses protocols detected significant differences between organic and conventional meat. | | | | | | | | | |
| --- | --- | --- | --- | --- | --- | --- | --- | --- | --- |
|  | **Standard meta-analysis** | | | | |  | **Sensitivity meta-analysis 1** | | |
| **Parameter** | ***n*** | **SMD** | **95% CI** | ***P**** | **Heterogeneity**† |  | ***n*** | **Ln ratio**‡ | ***P**** |
| Heptachlor | - | - | - | - | - |  | 3 | 4.61 | 1.000 |
| Hexachlorobenzene (HCB) | - | - | - | - | - |  | 4 | 4.32 | 0.505 |
| Lindane | - | - | - | - | - |  | 3 | 4.33 | 0.499 |
| Malathion | - | - | - | - | - |  | 3 | 4.61 | 1.000 |
| Methoxychlor | - | - | - | - | - |  | 3 | 4.61 | 1.000 |
| Methyl parathion | - | - | - | - | - |  | 3 | 3.94 | 0.503 |
| Mirex | - | - | - | - | - |  | 4 | 4.61 | 1.000 |
| Pirimiphos-Me | - | - | - | - | - |  | 3 | 4.61 | 1.000 |
| Ronnel | - | - | - | - | - |  | 3 | 4.57 | 0.489 |
| Trithion | - | - | - | - | - |  | 3 | 4.61 | 1.000 |
| α-benzene hexachloride (α-BHC) | - | - | - | - | - |  | 3 | 3.70 | 0.244 |
| β-benzene hexachloride (β-BHC) | - | - | - | - | - |  | 3 | 4.61 | 1.000 |
| δ-benzene hexachloride (δ-BHC) | - | - | - | - | - |  | 3 | 4.61 | 1.000 |
| *Other* |  |  |  |  |  |  |  |  |  |
| Campylobacter spp. | 3 | -0.01 | -0.23, 0.20 | 0.892 | No (0%) |  | 3 | 4.64 | 0.246 |
| pH§ | - | - | - | - | - |  | 12 | 4.61 | 0.066 |
| *n*, number of data points included in the comparison; SMD, standardised mean difference of random-effect model; CI, 95% confidence intervals; TBARS, thiobarbituric acid reactive substances method. **P* value <0.05 indicates significance of the difference in composition between organic and conventional meat; †Heterogeneity and the I^2^ Statistic; ‡Ln ratio = Ln(ORG/CONV × 100%); §Outlying data pairs (where the MPD between ORG and CONV was over fifty times greater than the mean value including the outliers) were removed. | | | | | | | | | |

| **Table S13.** Results of the statistical test for publication bias reported in Table 1 of the main paper. | | | | | |
| --- | --- | --- | --- | --- | --- |
|  | **Trim and fill test*** | | **No of missing *n* in Rosenthal’s Fail-safe N test**† | **No of missing *n* in Orwin’s Fail-safe N test**‡ | ***P* from Egger’s test for  funnel plot asymetry**§ |
| **Parameter** | **No of missing *n*** | **funnel plot side** |  |  |  |
| Fat | 0 | left | 199 | 22 | 0.079 |
| Intramuscular fat | 0 | right | 3 | 7 | 0.186 |
| SFA | 0 | right | 204 | 26 | 0.371 |
| 12:0 (lauric acid) | 2 | right | 0 | 11 | 0.874 |
| 14:0 (myristic acid) | 0 | right | 224 | 23 | <0.001 |
| 16:0 (palmitic acid) | 0 | right | 174 | 24 | <0.001 |
| MUFA | 0 | right | 1279 | 24 | 0.003 |
| OA (cis-9-18:1) | 0 | right | 164 | 22 | 0.103 |
| PUFA | 0 | left | 1364 | 23 | <0.001 |
| n-3 FA | 0 | left | 638 | 21 | <0.001 |
| ALA (cis-9,12,15-18:3) | 0 | left | 105 | 23 | <0.001 |
| EPA (cis-5,8,11,14,17-20:5)\|\| | 0 | left | 0 | 13 | 0.038 |
| DPA (cis-7,10,13,16,19-22:5) | 0 | left | 26 | 11 | 0.089 |
| DHA (cis-4,7,10,13,16,19-22:6) | 1 | right | 6 | 14 | 0.124 |
| VLC n-3 PUFA (EPA+DPA+DHA)¶ | - | - | - | - | - |
| n-6 FA | 0 | left | 519 | 19 | <0.001 |
| LA (cis-9,12-18:2) | 0 | left | 250 | 24 | 0.010 |
| AA (cis-5,8,11,14-20:4)\|\| | 0 | left | 42 | 13 | 0.064 |
| LA/ALA ratio¶ | - | - | - | - | - |
| n-6/n-3 ratio | 0 | right | 185 | 17 | 0.092 |
| SFA, saturated fatty acids; MUFA, monounsaturated fatty acids; OA, oleic acid; PUFA, polyunsaturated fatty acids; FA, fatty acids; ALA, α-linolenic acid; EPA, eicosapentaenoic acid; DPA, docosapentaenoic acid; DHA, docosahexaenoic acid; VLC n-3 PUFA, very long chain n-3 PUFA; LA, linoleic acid; AA, arachidonic acid. *The method used to estimate the number of data points missing from a meta-analysis due to the suppression of the most extreme results on one side of the funnel plot; †Number of missing data points that need to be retrieved and incorporate in the meta-analysis before the results become nonsignificant; ‡Number of missing data point that need to be retrieved and incorporate in the meta-analysis before the estimated value of the standardised mean (SMD) difference reaches a specified level (here SMD/2); §*P* value <0.05 indicates funnel plot asymmetry; \|\|Outlying data pairs (where the MPD between ORG and CONV was over fifty times greater than the mean value including the outliers) were removed; ¶Calculated based on published fatty acids composition data. | | | | | |

# 4. ADDITIONAL DISCUSSION

## **4.1.** The need to identify alternative approaches to increase VLC n-3 PUFA intake

North American and European agencies currently advise consumers to increase fish and especially oily fish consumption (e.g. salmon and herring) to improve VLC n-3 PUFA intake and thereby reduce the risk of cardiovascular disease^(^[^3^](#_ENREF_3)^)^. However, implementing these recommendations widely across the human population is thought to be impossible, since most of the world’s fish stocks are already over-exploited. Also there are concerns about the sustainability/environmental impacts of fish farming, mercury/dioxin contamination levels in n-3 PUFA rich fish in some regions of the world, while recent studies linked very high DHA intakes from oily fish/fish oil supplements with an increased prostate cancer risk^(^[^3-5^](#_ENREF_3)^)^. It is therefore thought essential to develop alternative strategies to increase VLC n-3 PUFA intakes (e.g. increasing VLC n-3 PUFA concentrations in meat, consumption of algae with high VLC n-3 PUFA content).

## **4.2.** The need to carry out additional studies comparing the mineral composition of meat from organic and conventional production

Due to the very limited evidence base it is not currently possible to estimate differences in mineral composition and potential impacts on human health. Additional well designed studies are therefore required to confirm trends identified in this study.

If the trend towards higher iron (Fe) concentration in organic meat (especially liver) were to be confirmed in future studies this could be nutritionally relevant/desirable, since iron deficiency anaemia remains a problem in reproductively active woman worldwide^(^[^6^](#_ENREF_6)^)^. Meat (especially red meat and organs such as liver) is known to be an important dietary source for Fe, and Fe from most animal sources (haem iron) is more readily absorbed than Fe from plant sources of food (non-haem iron)^(^[^7^](#_ENREF_7)^)^.

However, if future studies confirm that there is an approx. 25% lower copper intake with organic meat this is unlikely to have a significant health impact, since (1) estimated dietary Cu intakes in Europe (1.0-2.3 mg/day for males and 0.9-1.8 mg/day for females^(^[^8^](#_ENREF_8)^)^ are slightly above the recommended intakes for adults (0.9 mg/day)^(^[^9^](#_ENREF_9)^)^, (2) most Cu intake is with crop based foods such as cereals, vegetables and potatoes^(^[^10^](#_ENREF_10)^)^ and (3) no significant differences in Cu-concentrations between organic and conventional crops were detected in a previous literature review/meta-analysis^(^[^1^](#_ENREF_1)^)^. However, recent studies suggest that the incidence of chronic diseases (e.g. Alzheimer’s disease, ischemic heart disease and osteoporosis) associated with insufficient Cu-intakes may be underestimated^(^[^11^](#_ENREF_11)^)^.

# 5. ADDITIONAL REFERENCES

1. Barański M, Średnicka-Tober D, Volakakis N *et al.* (2014) Higher antioxidant and lower cadmium concentrations and lower incidence of pesticide residues in organically grown crops: a systematic literature review and meta-analyses. *Br J Nutr* **112**, 794-811.

2. Lipsey MW & Wilson DB (2001) *Practical meta-analysis. Applied social research methods series*. Thousand Oaks, CA, USA: Sage Publications.

3. Raatz SK, Silverstein JT, Jahns L *et al.* (2013) Issues of Fish Consumption for Cardiovascular Disease Risk Reduction. *Nutrients* **5**, 1081-1097.

4. Brasky TM, Till C, White E *et al.* (2011) Serum Phospholipid Fatty Acids and Prostate Cancer Risk: Results From the Prostate Cancer Prevention Trial. *Am J Epidemiol* **173**, 1429-1439.

5. Brasky TM, Darke AK, Song X *et al.* (2013) Plasma Phospholipid Fatty Acids and Prostate Cancer Risk in the SELECT Trial. *J Natl Cancer Inst* **105**, 1132-1141.

6. Miller EM (2014) Iron Status and Reproduction in US Women: National Health and Nutrition Examination Survey, 1999-2006. *PLoS ONE* **9**, e112216.

7. European Food Safety Authority (2006) Tolerable upper intake levels for vitamins and minerals. [www.efsa.europa.eu/fr/ndatopics/docs/ndatolerableuil.pdf](http://www.efsa.europa.eu/fr/ndatopics/docs/ndatolerableuil.pdf) (accessed 25th April 2013)

8. Van Dokkum W (1995) The Intake of Selected Minerals and Trace Elements in European Countries. *Nutr Res Rev* **8**, 271-302.

9. Food and Nutrition Board (2001) *Dietary Reference Intakes for Vitamin A, Vitamin K, Arsenic, Boron, Chromium, Copper, Iodine, Iron, Manganese, Molybdenum, Nickel, Silicon, Vanadium, and Zinc*. *A Report of the Panel on Micronutrients, Subcommittees on Upper Reference Levels of Nutrients and of Interpretation and Uses of Dietary Reference Intakes, and the Standing Committee on the Scientific Evaluation of Dietary Reference Intakes*. Washington, D.C., USA: Food and Nutrition Board, Institute of Medicine.

10. European Commission (2003) *Opinion of the Scientific Committee on Food on the Tolerable Upper Intake Level of Copper*. Brussels, Belgium: Health & Consumer Protection Diroctorate-General.

11. Klevay LM (2011) Is the Western diet adequate in copper? *J Trace Elem Med Biol* **25**, 204-212.
